# Physical, mechanical, and biological properties of collagen membranes for guided bone regeneration: a comparative in vitro study

**DOI:** 10.1186/s12903-023-03223-4

**Published:** 2023-07-22

**Authors:** Xiaolu Shi, Xianjing Li, Ye Tian, Xinyao Qu, Shaobo Zhai, Yang Liu, Wei Jia, Yan Cui, Shunli Chu

**Affiliations:** 1 Department of Implantology, Hospital of Stomatology, Jilin University, Changchun, China; 2 Department of Drug Clinical Trial, the Affiliated Hospital to Changchun University of Chinese Medicine, Changchun, China; 3 Yongchang Community Health Service Center of Chaoyang District, Changchun, China; 4 Department of Dermatology and Venereology, First Hospital of Jilin University, Jilin University, Changchun, China

**Keywords:** Guided bone regeneration, Barrier membrane, Collagen membrane, Degradation products, Bioactivity

## Abstract

**Background:**

To provide a reference for clinical selection of collagen membranes by analyzing the properties of three kinds of collagen membranes widely used in clinics: Bio-Gide membrane from porcine dermis (PD), Heal-All membrane from bovine dermis (BD), and Lyoplant membrane from bovine pericardium (BP).

**Methods:**

The barrier function of three kinds of collagen membranes were evaluated by testing the surface morphology, mechanical properties, hydrophilicity, and degradation rate of collagen membranes in collagenase and artificial saliva. In addition, the bioactivity of each collagen membrane as well as the proliferation and osteogenesis of MC3T3-E1 cells were evaluated. Mass spectrometry was also used to analyze the degradation products.

**Results:**

The BP membrane had the highest tensile strength and Young’s modulus as well as the largest water contact angle. The PD membrane exhibited the highest elongation at break, the smallest water contact angle, and the lowest degradation weight loss. The BD membrane had the highest degradation weight loss, the highest number of proteins in its degradation product, the strongest effect on the proliferation of MC3T3-E1 cells, and the highest expression level of osteogenic genes.

**Conclusions:**

The PD membrane is the best choice for shaping and maintenance time, while the BD membrane is good for osteogenesis, and the BP membrane is suitable for spatial maintenance. To meet the clinical requirements of guided bone regeneration, using two different kinds of collagen membranes concurrently to exert their respective advantages is an option worth considering.

**Supplementary Information:**

The online version contains supplementary material available at 10.1186/s12903-023-03223-4.

## Introduction

Bone defects and insufficient bone remain important challenges in dental implant restoration [1, 2] because they prevent dental implants from meeting the required bone width and height, thus hindering the application of dental implants. Guided bone regeneration (GBR) uses bone grafts or osteoinduction materials to promote bone reconstruction and stable blood clot formation [3]. In GBR, a barrier membrane is used to form a secluded space in the bone defect area to prevent fast-growing connective-tissue cells and epithelial cells from entering the defect area, thus allowing slow-growing osteoblasts to proliferate, differentiate, and mineralize in the defect area for bone tissue regeneration. Therefore, barrier membranes need to exhibit biocompatibility, maneuverability, tissue integrity, and bioactivity to meet the requirements of the clinical application [4, 5].

At present, commercial barrier membranes can be divided into two types: non-resorbable membranes, such as expanded-polytetrafluoroethylene (e-PTFE) membrane, and resorbable membranes, such as collagen membranes (Bio-Gide, Heal-All, etc.) and synthetic membranes (PLGA, PLDLA, etc.) [4]. Non-resorbable membranes may damage the regenerated tissue and burden the patient owing to unavoidable exposure to the oral enviroment and the inconvenience of a subsequent operation [6, 7]. Therefore, resorbable membranes are currently the most popular material because of their excellent biocompatibility and tissue integration, and, importantly, subsequent operations are unnecessary because they are biodegradable [2].

Commercial resorbable membranes, such as Bio-Gide membrane from porcine dermis (PD), Heal-All membrane from bovine dermis (BD), and Lyoplant membrane from bovine pericardium (BP), are predominantly based on collagen. The biological properties of collagen membranes vary significantly depending on the source and structure of the collagen. The microstructure of collagen membranes, such as fiber shape and direction, in addition to the source of the collagen and hydrophilicity of the membrane can affect the migration, proliferation, and differentiation of osteoblasts [8]. Moreover, GBR osteogenesis largely depends on the patient’s potential for bone healing (such as age and general nutritional status) and local conditions (such as blood vessels and embryological origin of bone) [9]. Therefore, to achieve desirable GBR outcomes, the biological properties of the collagen membrane and the patient’s bone healing ability need to be considered when selecting the collagen membrane. A large number of clinical data show that it takes at least three months to completely regenerate the bone in the jaw, which depends on jaw-intramembranous ossification, active angiogenesis of the jaw and its surrounding soft tissue, mechanical condition of the jaw, and effective stress shielding of the barrier membrane. Ideally, the degradation rate of the collagen membrane should match the rate of bone formation [4], and the collagen membrane should be effectively shielded from the rapidly proliferating epithelium during wound healing after surgery [3]. There are many kinds of commercially available collagen membranes, which makes it difficult to choose suitable membranes for individuals with different bone healing abilities and bone defects, especially in the absence of an index. For example, to support space maintenance for patients presenting with bone defects below Class 3, selecting collagen membranes with superior mechanical properties is preferred [10]. Therefore, it is necessary to fully characterize and compare the physical, mechanical, and biological properties of commonly used collagen membranes to provide a reference for the selection of collagen membranes.

In this study, the physical properties (surface morphology and hydrophilicity), mechanical properties, and degradation rate of three common collagen membranes were characterized. Additionally, the effects of degradation products (DPs) on the proliferation and differentiation of MC3T3-E1 cells were determined using assays. Furthermore, the composition of the DPs of the collagen membranes was also identified.

## Materials and methods

### Collagen membrane materials

The collagen membranes selected in this study meet the following criteria: The osteogenic ability of the membrane has been verified in animal experiments and clinical studies; the membrane has been widely used in clinical practice; and the collagen source or manufacturing process of the collagen membranes is different. On this basis, three kinds of collagen membranes were selected for this study: porcine dermis membrane (PD), bovine pericardium membrane (BP), and bovine dermis membrane (BD) (Table 1).

**Table 1:** Collagen membranes used for the study

Collagen Source	Brand	Manufacturer	LOT	Constituent	Abbreviations
Porcine dermis	Bio-Gide	Geistlich Pharma AG, Wolhusen LU, Switzerland	82101099	Heterologous collagen	PD
Bovine pericardium	Lyoplant	B.Braun Biotech International, Melsungen, Germany	220382	Heterologous collagen	BP
Bovine dermis	Heal-All	Zhenghai Biotechnology Co., LTD, Yantai, China	SS200803	Heterologous collagen	BD

### Surface morphology

The surface morphology of the three membranes was analyzed using scanning electron microscopy (SEM, Hitachi S-4800, Hitachi Co., Ltd., JPN). The membranes were cut to a size of 1 mm × 1 mm. After vacuum gold plating, the micro-morphology of the smooth and rough surfaces were observed and recorded with a scanning electron microscope operating at an acceleration voltage of 1 kV [11].

### Mechanical property

Each collagen membrane was divided into two groups: dry and wet. Collagen membranes in the wet group were soaked in artificial saliva (Solarbio, Beijing, China) for 2 min before testing. The thickness of the sample was measured with a leather thickness measuring instrument (Kunshan Xiangke Testing Instrument Co., Ltd., Kunshan, China), and six test points were randomly selected on each membrane to calculate the average value. The tensile strength, elongation, and Young’s modulus of the collagen membranes were evaluated using a universal testing machine (ZwickRoell GmbH & Co.KG, Ulm, Germany). Each membrane was cut into 20 mm × 3.0 mm specimens and mounted on the gripping unit of the tester. A tensile force was applied at a crosshead speed of 5 mm/min until the specimen broke (*n* = 5). All measurements were performed at room temperature. The Young’s modulus of the membrane is the slope of the linear elastic part of the stress–strain curve. The tensile strength and elongation at break were calculated using the following equations: Tensile strength (MPa) = Maximum load at break (N)/Cross-sectional area (m^2^) [11] and Elongation (%) = (Break length − Initial length)/Initial length × 100 [12].

### Hydrophilic property

The hydrophilicity of the front and back sides of the collagen membranes was evaluated by measuring the contact angles. In summary, 3 μL of distilled H_2_O was dropped on the surface of the collagen membrane, and then the contact angle was measured with an optical contact angle measuring instrument (Theta Flex, Biolin Technology, Gothenburg, Sweden) at room temperature. Measurements were repeated for a total of 5 times, and the results were averaged.

### Degradation ratio assay

The three types of collagen membranes were sheared into circle (diameter 13 mm) and transferred to a 15-mL centrifuge tube. Then, type I collagen solution (final concentration of 10 U/mL, Sigma-Aldrich, Saint Louis, USA) and artificial saliva (Solarbio, Beijing, China) were added, with a sterilized filter, giving a final volume of 10 mL (10 specimens in each group). The reaction mixture was incubated at 37 °C, 130 rpm. The residual specimens were taken out on days 7, 14, and 21, then freeze-dried, and finally weighed [13]. The degradation ratio was expressed as the percentage of weight loss, calculated using the following equation: Weight loss (%) = (W_0_ − W_d_)/W_0_, where W_0_ is the actual weight before degradation and W_d_ is the dry weight after degradation.

### Preparation of Degradation Products (DPs)

For subsequent assays, the DPs of the collagen membranes were prepared as follows. Briefly, the collagen membranes (5 mm in diameter) were enzymatically hydrolyzed with or without 20 U/mL collagenase solution, and PBS controls were established (triplicate samples in each group). After incubation at 37 °C and 130 rpm for 24 h, the supernatant of each sample was collected with a centrifuge (4230 rpm for 5 min). The protein concentrations were determined with a bicinchoninic acid (BCA) assay kit (Beyotime, Shanghai, China).

After desalting with a dialysis bag (3 kDa MWCO, Genview, Beijing, China), DPs were characterized with 15.5% tricine-sodium dodecyl sulfate (SDS)-polyacrylamide gel electrophoresis (PAGE). The tricine-SDS-PAGE gel preparation kit, tris-tricine-SDS buffer solution, and protein standard samples were all obtained from Sangon Biotech (Shanghai, China). Sample treatment, gel preparation, and electrophoresis were performed as previously described by Schagger [14]. The gel was stained with Coomassie brilliant blue and imaged using a gel imaging system (Clinx., Shanghai, China).

### Cell culture

MC3T3-E1 cells were purchased from the Cell Bank of the Chinese Academy of Sciences (Shanghai, China) and cultured in high-glucose Dulbecco’s modified Eagle’s medium (DMEM, Hyclone, Logan, USA) supplemented with 10% fetal bovine serum (FBS, Biologic Industries, Kibbutz Beit Haemek, Israel) and 1% penicillin-streptomycin (Hyclone, Logan, USA). Cells were maintained at 37 °C, 5% CO_2_, and 95% humidity. The cells were passaged at 75%–90% confluence using trypsin (Hyclone, Logan, USA). For all experiments, the cells were seeded on 96-well plates, 24-well plates, or 6-well plates, at a density of 2 × 10^3^, 2 × 10^4^, or 8 × 10^4^ cells/well, respectively [15].

### Cell proliferation

Proliferation of MC3T3-E1 cells was evaluated using the cell counting kit 8 (CCK8) method. After 1 day of incubation, MC3T3-E1 cell medium was replaced with 100 μL of complete medium containing 50 μL of DPs, acquired as described above, to serve as the treatment group. Cells cultured with complete medium only served as the control group, and medium only (without cells) served as the blank group. Triplicate samples were prepared for each group. At days 1, 3, and 5, 10 μL of CCK8 (Beyotime, Shanghai, China) was added to the well, and the absorbance (OD) at 450 nm was recorded with a microplate reader (Bio-Tek, Winooski, USA). Cell viability was calculated using the following equation: Cell viability = (Treatment Group OD−Blank Group OD)/(Control Group OD−Blank Group OD) × 100% [16].

### Alkaline Phosphatase (ALP) activity

After 1 day of incubation, MC3T3-E1 cell medium was replaced with 500 μL of osteoinduction differentiation medium (ODM; 50 μg/mL ascorbic acid, 100 nM DEX, and 5 mM β-glycerolphosphate) containing 250 μL of DPs, serving as the experiment group. Cells cultured with ODM only served as the control group, and normal medium only (without cells) served as the blank group. Triplicate samples were prepared for each group. After 1, 7, and 14 days, cells were lysed with deionized water and homogenized via ultrasound at 4 °C. The cell lysates were transferred to a 96-well plate. ALP activity and total protein concentration were measured and quantified using an ALP activity kit (Jiancheng Bioengineering Institute, Nanjing, China) and a BCA assay kit. ALP activity was normalized by the corresponding total protein concentration (U/mg) following the kit instructions [15].

### Real-time quantitative polymerase chain reaction (qPCR)

Total RNA was extracted with TRIzol (Invitrogen, Carlsbad, USA) and then converted to cDNA with Hifair II 1st Strand cDNA Synthesis Kit (Yeasen, Shanghai, China) following the kit instructions. SYBR green PCR Master Mix (Takara, Dalian, China) was used for real-time qPCR on an MX 3000 platform (Agilent, Boeblingen, Germany). The primers for real-time qPCR were as follows: Runx2, 5’-ATAGCAAAGGCCCTCACTAA-3’ (forward) and 5’-AACTGGCTCTTCTGCTGATT-3’ (reverse); Col 1, 5’-GAGGCATAAAGGGTCATCGTGG-3’ (forward) and 5’-CATTAGGCGCAGGAAGGTCAGC-3’ (reverse); OC, 5’-TGACCTCACAGATGCCAAGC-3’ (forward) and 5’- CGCCGGAGTCTGTTCACTAC-3’ (reverse); and GAPDH, 5’- ACCACAGTCCATGCCATCAC-3’ (forward) and 5’-TCCACCACCCTGTTGCTGTA-3’ (reverse).

The expression levels of each target gene were normalized to the corresponding GAPDH threshold cycle (CT) values using the 2−▵▵CT comparative method [16].

### High-performance Liquid Chromatography (HPLC) and mass spectrometry

The three types of collagen membranes were subjected to enzymatic hydrolysis for 21 days. DPs were collected by filtration–centrifugation using a Millipore centrifugal device (10 kDa MWCO, Millipore, Milford, MA). The resulting fractions were desalted using a C18 column (Thermo Scientific, San Jose, USA), then lyophilized, and finally stored at −20 °C until analyzed. Mass spectrometric analyses were performed as previously described [17]. The lyophilized peptides were separated using an Ultimate 3000 RSLCnano system coupled to a Q Exactive (Thermo Scientific, USA). Samples were loaded into a trap column (C18, 3 μm, 120 Å, 100 μm × 2 cm) and separated with a reversed-phase analytical column (C18, 2μm, 100 Å, 75 μm × 150 mm, Thermo Scientific, USA). Peptides were separated using a gradient of mobile phase A (3% dimethyl sulfoxide, 0.1% formic acid, and 97% H2O) and B (3% dimethyl sulfoxide, 0.1% formic acid, and 97% acetonitrile). The flow rate was set to 300 nL/min. The mass spectrometer was operated in data-dependent acquisition (DDA) mode with the following settings: Full MS scan (R = 70 K, AGC = 3e6, max IT = 20 ms, scan range = 350–1800 m/z) followed by up to 15 MS/MS scans (R = 17.5 K, AGC = 2e5, max IT = 100 ms). The isolation window was set to 1.6 m/z, and 28% normalized collision energy was used for higher-energy collisional dissociation (HCD). The dynamic exclusion time of repeated ion acquisition was set to 35 s [17].

The acquired raw mass spectrometric data were processed in MaxQuant (v1.6.2.10) [18] and searched against UniProt for taxonomy: “Sus scrofa” (TaxID 9823) for PD and “Bos taurus”(TaxID 9913) for the other two collagen membranes. All data and search results have been deposited to the iProX database ( http://www.iprox.org) with the iProX accession: IPX0006335000.

### Statistical analysis

Data analysis and figure construction was performed using GraphPad Prism 6 (GraphPad, San Diego, USA). Values represent mean ± standard deviation (SD). Comparison among different groups was made by two-way analysis of variance (ANOVA). *P* values <0.05 were considered statistically significant.

## Results

### Physical characteristics of collagen membranes

**Fig. 1:**
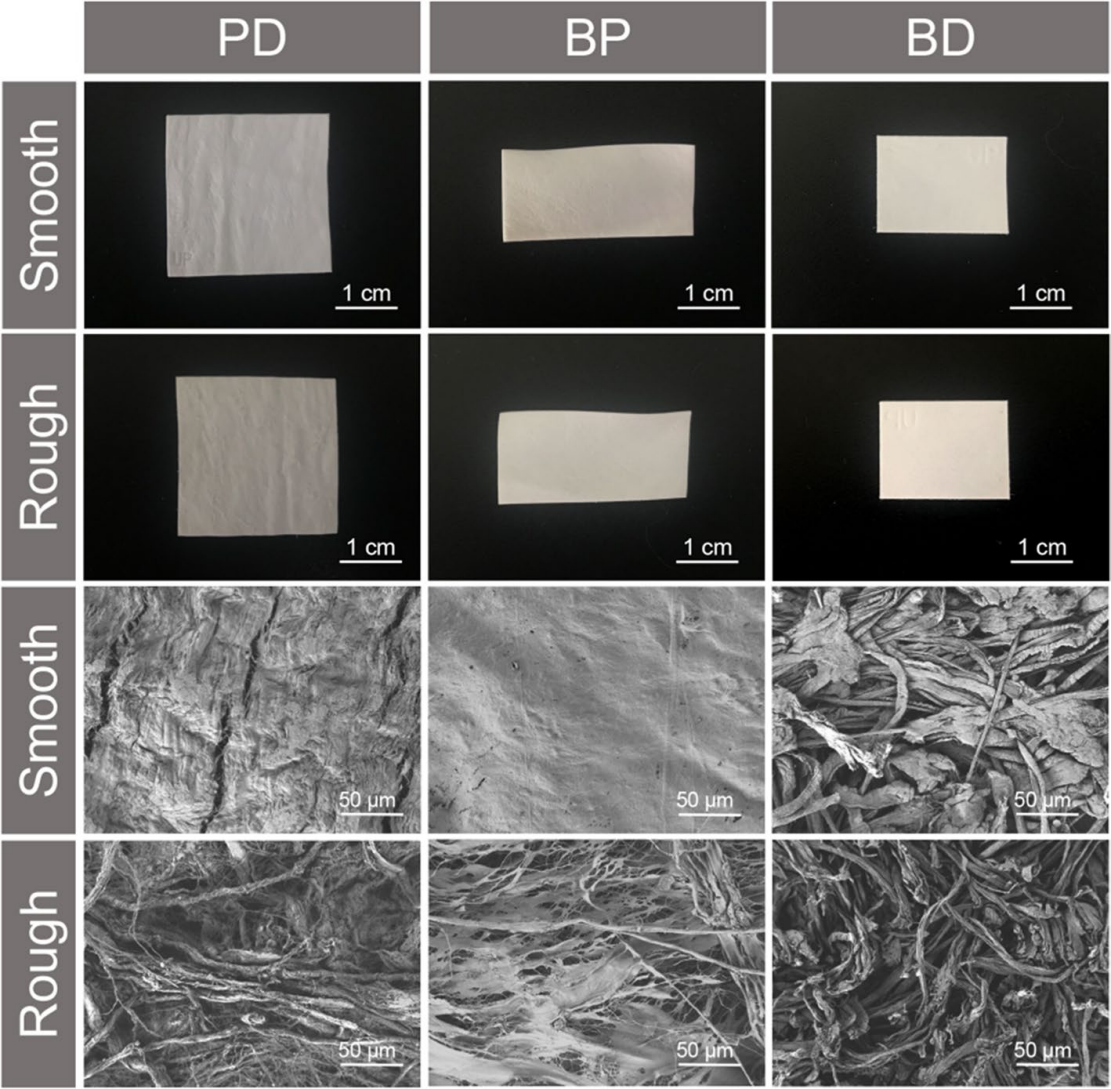
Gross appearance (scale bar: 1 cm) and SEM (scale bar: 50 μm) images of collagen membranes

Gross appearance was determined: The smooth surface of all collagen membranes was soft and elastic, while the texture of the rough surface was irregular (upper part, Fig. 1). SEM images revealed marked differences in the microstructures of the collagen membranes (lower part, Fig. 1). On the smooth side, collagen bundles of the PD and BP membranes were regular and arranged closely, while collagen bundles of the BD membrane were slightly irregular and arranged loosely. On the rough side, collagen bundles of BP interweaved with each other to form a three-dimensional (3D) grid with regular pores, while collagen bundles of PD and BD were arranged loosely.

**Fig. 2:**
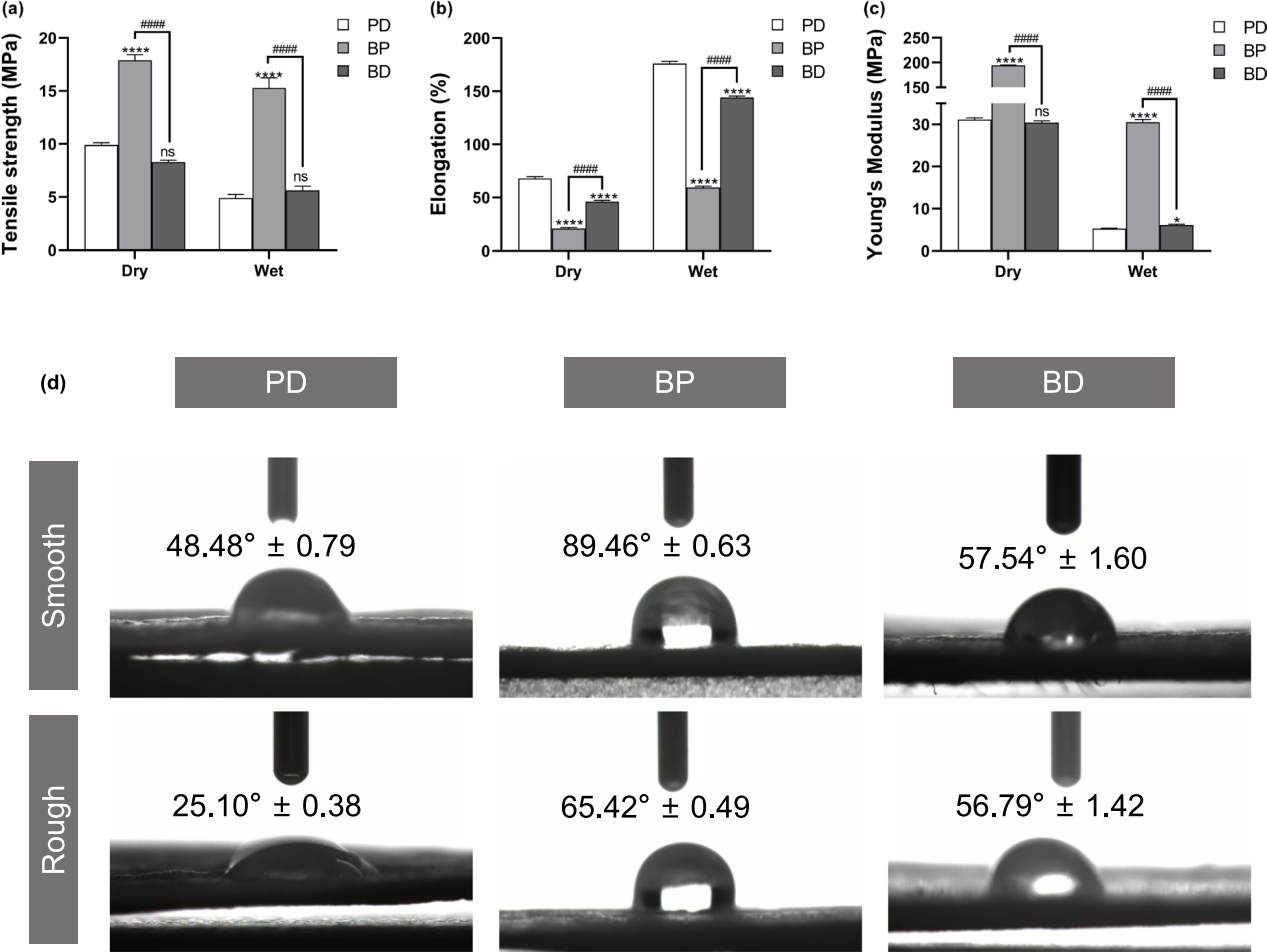
Comparison of the physical characteristics of different collagen membranes. **a** Tensile strength, **b** elongation at break, and **c** Young’s modulus of collagen membranes in the dry and wet state. The error bars represent ± SD. Two-way analysis of variance analysis. ns: not significant; ****P < 0.0001 vs PD; #### *P* < 0.0001 vs BP. *n* = 3 for all samples. **d** Water contact angle of collagen membranes. Mean ± SD shown. *n* = 5 for all samples

The mechanical properties (tensile strength, elongation, and Young’s modulus) of the three kinds of collagen membranes in both the dry and wet states were compared (Fig. 2a–c). The results indicated that the tensile strength and Young’s modulus of the BP membrane in both the dry and wet states were significantly higher than those in other groups (*P* < 0.0001), although no significant difference was found between the BD and PD membranes (*P* > 0.05) except the Young’s modulus in the wet state (*P* = 0.034). Elongation was the highest for the PD membrane, irrespective of the state, intermediate for the BD membrane, and lowest for the BP membrane. Moreover, for all collagen membranes, the tensile strength and Young’s modulus were lower in the wet group than in the dry group, while elongation showed the opposite trend.

To assess the hydrophilicity of the three kinds of collagen membranes, static water-contact angles were measured. As shown in Fig. 2d, the water contact angle was significantly higher on the smooth surface than on the rough surface of the PD and BP membranes. However, the water contact angle was not significantly different between the smooth and rough surfaces of the BD membrane. On both smooth and rough surfaces, the water contact angle was the smallest for the PD membrane and the largest for the BP membrane.

### Degradation ratio of collagen membranes

**Fig. 3:**
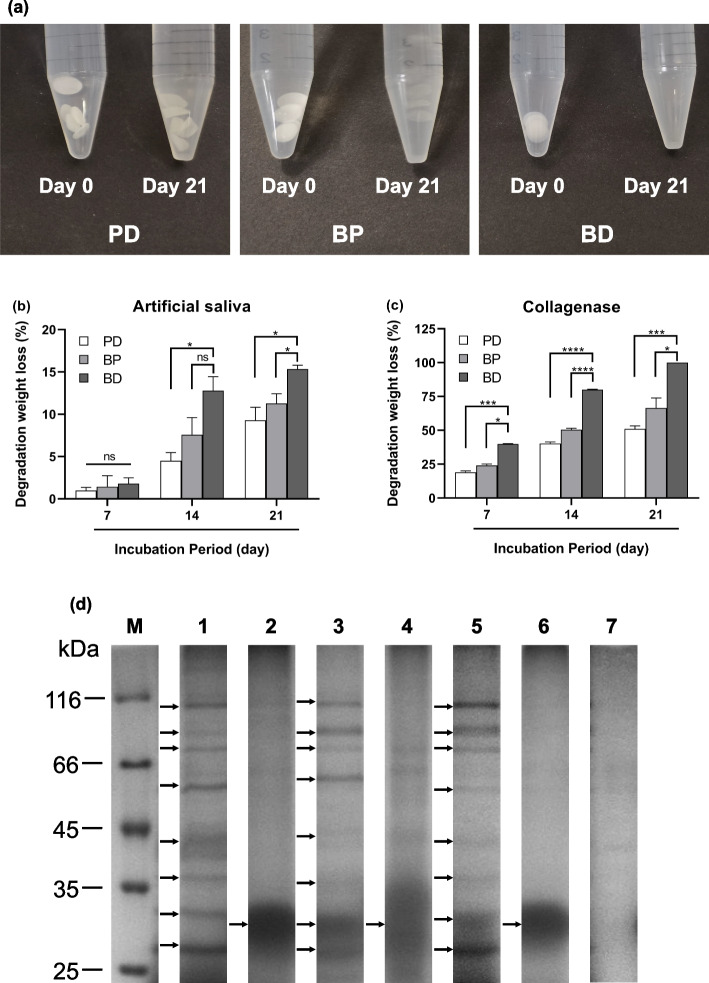
Comparison of the degradation of different collagen membranes. **a** Photographs of collagen membranes before and 21 days after degradation using collagenase. Degradation weight loss (%) of the collagen membranes incubated with artificial saliva (**b**) and collagenase (**c**) after 7, 14, and 21 days. The error bars represent ± SD. Two-way analysis of variance analysis. ns: not significant, *P < 0.05, ***P <0.001, ****P < 0.0001 vs PD. *n* = 3 for all samples. **d** SDS-PAGE-Tricine gel electrophoresis. M: low molecular weight marker. Lane 1 and 2: PD incubated with or without collagenase; Lane 3 and 4: BP incubated with or without collagenase; Lane 5 and 6: BD incubated with or without collagenase; Lane 7: Collagenase solution

To determine the degradation ratio of the collagen membranes, the oral environment was mimicked using artificial saliva and collagenase. As shown in Fig. 3a, the PD and BP membranes became translucent within 21 days of treatment with collagenase, and, unexpectedly, the BD membrane was completely degraded. The degradation weight loss also confirmed the above findings. For the artificial saliva group, there was no significant difference among the three types of membranes on day 7. Moreover, the degradation weight loss increased gradually with treatment time up to day 21. The BD membrane suffered the highest degradation weight loss, followed by the BP membrane and then the PD membrane. By contrast, the degradation weight loss decreased in the collagenase group on day 7. On day 21, the PD membrane suffered the lowest degradation weight loss. The degradation weight loss of the BD membrane was up to 100%, indicating complete degradation of the BD membrane (Fig. 3b, c). The results demonstrate that the BD membrane is the most susceptible to degradation among the three membranes, irrespective of the treatment: artificial saliva or collagenase.

On the basis of the above results, collagenase was used to degrade the collagen membranes in subsequent experiments.

After incubating for 24 h, DPs were analyzed to determine the protein concentration, as shown in Table 2. The protein concentration of degraded collagen membranes in the collagenase group was significantly higher than that in the PBS group (*P* < 0.0001). As expected, the BD membrane had the highest protein concentration of DPs, specifically it was 1.4 times than those of the other membranes, followed by the BP membrane and then the PD membrane. However, DPs from the three collagen membranes treated with collagenase had a similar composition. Eight protein bands were observed in the electrophoretic gel with molecular weights ranging from 25 to 116 kDa (Fig. 3d).

**Table 2:** Total protein concentration of the degradation products of collagen membranes was measured by BCA method (*n*=5, mean ± SD)

Collagen Source	Total Protein Concentration (μg/mL)	
	Collagenase Group	PBS Group
Porcine dermis	823.34 ± 7.05	131.06 ± 4.49
Bovine pericardium	720.66 ± 10.62	17.46 ± 3.01
Bovine dermis	1279.78 ± 6.5	231.23 ± 4.52

### Effects of DPs on MC3T3-E1 cells

**Fig. 4:**
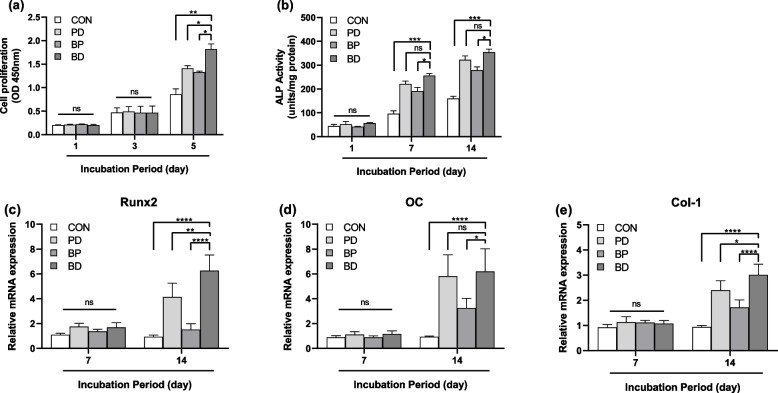
Effects of collagen membranes degradation products on the proliferation and differentiation of MC3T3-E1 cells. CCK-8 proliferation assay performed with and without collagen membranes degradation products after 1, 3, and 5 days. **b** Alkaline phosphatase (ALP) activity of MC3T3-E1 cells incubated with or without collagen membranes degradation products for 1, 7, and 14 days. ALP activity was normalized by total cellular protein amounts. qPCR analysis of osteoblastic marker (**c**) runt-related transcription factor 2 (Runx2), (**d**) osteocalcin (OC), and (**e**) collagen type I (Col-1) relative expression in MC3T3-E1 cells incubated with or without collagen membranes degradation products for 7 and 14 days. The error bars represent ± SD. Two-way analysis of variance analysis. ns: not significant, *P < 0.05, **P < 0.01, ***P < 0.05, ****P < 0.0001. *n* = 3 for all samples

The proliferation of MC3T3-E1 cells was measured after MC3T3-E1 cells were cultured with collagen membrane DPs for 1, 3, and 5 days. The effect of DPs on the proliferation of MC3T3-E1 cells was correlated with the incubation time. In general, differences in cell proliferation were not significant between the control and groups with added DPs until day 5 (*P* < 0.05, Fig. 4a). Cell proliferation was the highest in the presence of BD-DPs, intermediate in the presence of PD-DPs, and lowest in the presence of BP-DPs.

Similar to the cell proliferation results, ALP activity of cells treated with DPs increased with the incubation time and reached the peak on day 14, compared with the control group. Moreover, ALP activity of cells was the highest in the BD-DPs group, intermediate in the PD-DPs group, and lowest in the BP-DPs group (Fig. 4b).

To determine the effect of DPs on mineralization, the gene expressions of Runx2, OC, and Col 1, well-known osteoblast markers, were evaluated by real-time qPCR. As shown in Fig. 4c–e, after 7 days of treatment, when compared with the control group, there are no statistical differences in the OC, Runx2, and Col 1 expressions. However, when we prolonged the treatment to 14 days, all gene expressions noticeably increased. In addition, BD-DPs induced a higher level of Runx2, OC, and Col 1 gene expressions than PD-DPs and BP-DPs.

### Analysis of the DPs of collagen membranes

**Fig. 5:**
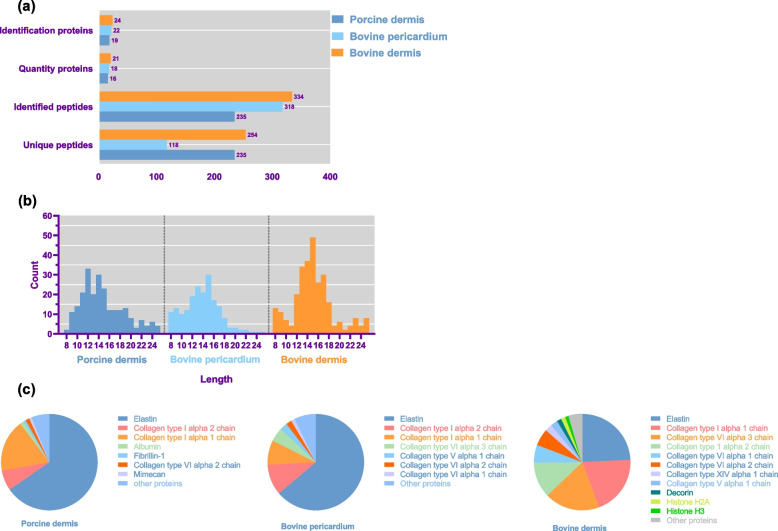
Analysis of peptides identified by LC–MS after in vitro degradation of three collagen membranes. **a** Identified proteins and peptides. **b** Length distribution of peptides (amino acids). X-axis represents the length of the peptides, and y-axis represents count of peptides. **c** Protein origin distribution

Excluding common contaminating peptides and low abundance peptides, a total of 887 peptides were identified in the degradation products of the three collagen membranes by liquid chromatography-mass spectrometry (LC–MS). Among them, 334 peptides, derived from 24 proteins, were identified in the BD membrane. Fewer peptides were identified in the PD membrane, and the lowest number of peptides was identified in the BP membrane (Fig. 5a). The peptides ranged from 8 to 25 amino acids in length (Fig. 5b). Despite the differences in peptide sequences, the identified proteins, including collagen and elastin, were similar (Fig. 5c and Supplementary Table S1 and S2).

## Discussion

The three kinds of collagen membranes display unique features in clinical applications. The PD membrane can resist degradation for a long time, thus achieving significant bone growth effect in clinical applications by virtue of its excellent histocompatibility and reliable biosafety [19, 20]. The potential of the BD membrane in promoting tissue healing has been widely explored, and it is often used to guide tissue regeneration in the clinic [21, 22]. The BP membrane is known for its excellent tensile strength and mechanical properties [23, 24]. These membranes with different characteristics have shown consistent osteogenic efficacy in animal models and clinical trials [21, 25, 26]. However, these data primarily arise from clinical observations and studies of a membrane in isolation of other membranes, not from a comparison between different membranes.

First, the tensile strength and Young’s modulus of the three collagen membranes, important indicators of the resistance of solid materials to deformation, were obtained. Elongation, a vital index to characterize the softness and elasticity of fibers, was also evaluated. The experimental results showed that the tensile strength and Young’s modulus of the BP membrane were significantly higher than those of the other membranes, reflecting its excellent spatial maintenance ability. Among the three membranes, the PD membrane had the highest elongation, indicating excellent plasticity. In addition, as common occurrences in clinical treatment, insufficient soft tissue at the bone defect and high wound suture tension are risk factors leading to partial leakage of the membrane, thus exposing the membrane to the oral cavity. Previous research showed that a moist environment has a significant impact on the mechanical properties of the membrane [27]. That’s why, to demonstrate the effect of oral saliva on the mechanical properties of collagen membranes, both the dry and wet groups were evaluated. The results showed that the tensile strength and Young’s modulus of the wet group were lower than those of the dry group, consistent with the results of previous studies [28]. It is well established that the mechanical properties of resorbable membranes are poorer than those of non-resorbable membranes [29]. Therefore, if collagen membranes are selected as the barrier membrane, clinicians should reduce the extra pressure, avoid overstretching the collagen membrane when fixing it with membrane tacks or sutures, and avoid unnecessary stretching when pre-trimming the collagen membrane before implantation. When additional stress is inevitable or more support space is needed, the BP membrane is the most suitable candidate among the three membranes because it exhibits high tensile strength, which minimally decreases after wetting, and it has the largest Young’s modulus, reflecting its greater stiffness. Furthermore, extra support, such as bone graft materials, membrane tacks, and suture retention, can be given to compensate for the mechanical strength of the membrane.

The hydrophilicity of the collagen membranes was evaluated by measuring the water contact angles. Generally, surfaces with low contact angles exhibit strong biocompatibility and tissue integration. Conversely, surfaces with high contact angles can reduce cell adhesion and tissue regeneration [30], which is related to positioning the smooth surface to face the suture surface of the wound and the rough surface to face the bone defect area in clinical applications. The contact angle on both the smooth and rough surfaces of the PD membrane was the lowest, while that of the BP membrane was the highest. The hydrophilicity of the collagen membrane may be related to the arrangement of collagen fibers on the surface, specifically a looser arrangement tends to correlate with a lower contact angle and higher hydrophilicity [31]. This is consistent with the results of this study, specifically the hydrophilicity of the PD and BP membranes is higher on the rough surface than on the smooth surface, and the rough surface has a looser arrangement of the microstructure than the smooth surface.

In contrast to non-resorbable membranes, collagen membranes are biodegradable, which is a distinct advantage because a subsequent operation is unnecessary. In vivo studies of the collagen membranes show that these membranes significantly degrade within 8 to 12 weeks [32, 33]. In addition to cellular and enzymatic microenvironments under microbe-free conditions, spontaneous exposure to the oral enviroment is essential for the degradation of resorbable membranes [34–37]. Upon exposure to the oral environment, resorbable membranes degrade rapidly in the presence of saliva and collagenase derived from periodontal pathogens, such as Porphyromonas gingivalis and Bacteroides melaninogenicus [38]. Considering the requirements of clinical applications, degradation of membrane materials benefits the healing of damaged tissues. Even in the case of exposure to the oral environment, the exposed membrane should be kept in situ, thus continuing to function during the regenerative process [39, 40]. Therefore, artificial saliva and collagenase were introduced to evaluate the effects of the degradation of collagen membranes. In the present study, among the three membranes, the BD membrane degrades with the highest rate, while the PD membrane degrades with the lowest rate. Although in vitro studies have limitations in simulating the oral environment, the results of this experiment are consistent with those of Neto et al., that is, the PD membrane is less degraded than the BP membrane during bone regeneration in a rabbit bone defect model [41]. The PD membrane derived from porcine dermis is denser and degrades with a lower rate than the BD membrane, which is derived from bovine dermis, indicating that the degradation rate is related to the structure and source of the collagen [42].

It is well established that collagen peptides can promote osteogenesis by enhancing osteoblast (MC3T3-E1 cells) proliferation and differentiation [43, 44]. Although collagen membranes are known to provide a conducive environment for osteoblasts to adhere, survive, and grow, they are not considered a key player in promoting osteogenesis [45–47]. Because the three kinds of collagen membranes were found to have different degradation rates, this experiment explored the effect of DPs on bone defects during GBR by evaluating the proliferation of MC3T3-E1 cells, the activity of ALP (an osteoblast differentiation marker), and the messenger ribonucleic acid (mRNA) expression level of bone formation-related genes (Runx2, Col1, and OC). The results showed that the DPs of the three kinds of collagen membranes enhanced cell proliferation and ALP activity, in addition to up-regulating the mRNA expression level of osteoblast-related genes. This is the first demonstration, to our knowledge, showing that degradation products of collagen membrane can promote osteogenesis. Among the three membranes, the BD membrane is the most effective, followed by the PD membrane, in promoting osteogenesis. This high performance is likely due to the high total collagen in the BD membrane compared with that in the other membranes. Another reason is related to the source of the collagen.

Collagen membranes are composed of numerous proteins, including fibrillar collagens, non-fibrillar collagens, and leucine-rich repeat proteoglycans, as well as a small number of structural proteins, such as vimentin, actin-based microfilaments, annexins, tubulins, and histones [48]. Consistent with other studies, of the 887 peptides identified in the degradation products of the three collagen membranes, a large number is derived from different collagen chains and elastin. Interestingly, collagen peptides from different sources exhibit a wide range of biological functions, such as anti-inflammation, wound-healing, and anti-oxidative stress [49–51]. Two bioactive peptides, C2 and E1, derived from bovine tendon collagen can support cell adhesion and counter stress [52]. Liu et al. [53] also found that bovine collagen peptides with different molecular weights have different effects on the differentiation and mineralization of osteoblasts. In the present study, by identifying the DPs of the three collagen membranes, a pool of collagen peptides is available for the screening of peptides capable of promoting osteogenesis. A common strategy to extend the function of collagen membranes is to load or modify the membrane with bioactive components [46, 54]. Yu et al. modified a collagen membrane with stromal cell-derived factor-1 alpha (SDF-1α), a pro-osteogenic protein, by chemical conjugation, and reported that the modified membrane significantly promotes new bone and micro vessel formation [46]. Chen et al. used sonication to coat a collagen membrane with silver nanoparticles, thus conferring excellent anti-bacterial activity against Staphylococcus aureus and Pseudomonas aeruginosa [55]. These modified membranes not only require redesign of the bioactive component but also lack evaluation of biocompatibility. In contrast, collagen peptides derived from the degradation products of collagen membranes are potentially safer than the above bioactive components. Therefore, in future studies, identifying peptides with high bioactivity among the DPs of collagen membranes can be attempted to develop functional collagen membranes.

## Conclusion

In this study, the physical, mechanical, and in vitro biological properties of three kinds of collagen membranes were determined. The findings indicate that the PD membrane is the best choice for shaping and maintenance time, the BD membrane is good for osteogenesis, and the BP membrane is suitable for spatial maintenance. More importantly, two kinds of collagen membranes can be used concurrently to exert their respective advantages for clinical needs during GBR, for example, placing the PD membrane as the outer layer to prolong maintenance time and using the BD membrane as the inner layer for osteogenesis. Further studies are necessary to determine the osteogenic effect of single and combined membranes on GBR. Because of the limitations of in vitro experiments, in vivo experimental studies are needed to provide clinicians with reliable indicators.

## Supplementary Information

**Additional file 1:** **Supplementary Table S1.** Identified proteins.

**Additional file 2:** **Supplementary Table S2.** Identified peptides.

**Additional file 3:** **Figure 3.** Original figure of Figure 3d in the manuscript. (A) Original figure. (B) The picture after exposure. The red boxes represent the regions of the original blots used in main figures. The label (M,1,2,3,4,5,7,6) matched to the cropped versions in the manuscript figures.

## Data Availability

The data that support the findings of this study are available from the corresponding author upon reasonable request.
