# Supplementary material for: Physical, mechanical, and biological properties of collagen membranes for guided bone regeneration: a comparative in vitro study
Source: BMC Oral Health. 2023 Jul 22;23:510. doi: 10.1186/s12903-023-03223-4 (PMC10362553; doi:10.1186/s12903-023-03223-4)
Supplement: Supplementary file 1 — Additional file 1: Supplementary Table S1. Identified proteins. [file 12903_2023_3223_MOESM1_ESM.pdf]

Porcine dermis

Accession	Gene	Description	Mw(kDa)	Length	Protein group	Peptides	Unique peptides	Sequence coverage [%]	Unique sequence coverage [%]	Intensity	iBAQ	iBAQ [%]	MS/MS count	SAF	Protein group ID
A0A287AXU0	ELN	Elastin OS=Sus scrofa OX=9823 GN=ELN PE=1 SV=2	58.277	674	A0A5G2R7W7;I3LP72;A0A5G2Q717;A0A5G2R699;A0A5G2R338;A0A5G2QQ49	153	153	50	50	70976000000	NA	NA	216	0.320474777	12
F1SFA7	COL1A2	Fibrillar collagen NC1 domain-containing protein OS=Sus scrofa OX=9823 GN=COL1A2 PE=1 SV=2	104.442	1135		16	16	3.7	3.7	5911200000	NA	NA	89	0.078414097	15
A0A287BLD2	COL1A1	Uncharacterized protein OS=Sus scrofa OX=9823 GN=COL1A1 PE=1 SV=1	138.053	1451	A0A287A1S6;A0A5G2QQE9	41	41	4.7	4.7	5241000000	NA	NA	68	0.046864232	10
A0A287AFS2	ALB	Albumin OS=Sus scrofa OX=9823 GN=ALB PE=1 SV=2	56.708	494	A0A287AMK0;A0A286ZT13;P08835;F1RUN2;A0A287BAY9	3	3	5.5	5.5	218210000	NA	NA	6	0.012145749	1
A0A5G2QMD8	FBN1	Fibrillin-1 OS=Sus scrofa OX=9823 GN=FBN1 PE=1 SV=1	307.891	2829	F1SN67;Q9TV36	2	2	0.9	0.9	49819000	NA	NA	5	0.001767409	3
I3LQ84	COL6A2	Collagen type VI alpha 2 chain OS=Sus scrofa OX=9823 GN=COL6A2 PE=1 SV=3	101.38	943		3	3	2.1	2.1	31657000	NA	NA	3	0.003181336	18
A0A0H5ANC0	OGN	Mimecan OS=Sus scrofa OX=9823 GN=OGN PE=1 SV=1	33.986	298		2	2	8.4	8.4	10329000	NA	NA	2	0.006711409	5
A0A287B5M9	COL6A1	VWFA domain-containing protein OS=Sus scrofa OX=9823 GN=COL6A1 PE=1 SV=2	27.825	249	I3LS72;A0A5G2Q7R0;A0A5G2QW87	1	1	4	4	13505000	NA	NA	2	0.008032129	13
A0A287BLM4	COL6A3	Uncharacterized protein OS=Sus scrofa OX=9823 GN=COL6A3 PE=1 SV=1	181.699	1673	A0A287BL81;A0A286ZLV2;A0A286ZMC0;A0A287BPF4;A0A286ZPQ1;A0A5G2R8G7;A0A287B163;A0A286ZVG7;I3LUR7	2	2	1.5	1.5	34744000	NA	NA	2	0.001195457	6
A0A5G2QY90	ACTA2	Uncharacterized protein OS=Sus scrofa OX=9823 GN=ACTA2 PE=3 SV=1	34.74	313	A0A5G2QIZ5;A0A287BGY0;A0A5S6G831;A0A286ZWJ1;F1SLG5;F6Q364;A0A287A4R1;A0A287AA77;A0A287A5G1;C7AI81;B6VNT8;P68137;A0A480K2S5;I3LVD5;Q6QAQ1;A0A287AXB6;A0A5G2QKZ4;A0A5G2R0X5;A0A5G2QZX1;A0A287AAR4;A0A287AVS8;A0A287A6R7;A0A5S6I3N7;A0A287BF33;A0A5G2QZ37;A0A287B0H6;A0A5G2QX85;A0A5G2QID5;A0A5S6IE01	2	2	7.7	7.7	12919000	NA	NA	2	0.006389776	2
A0A287AZY1	GGA3	Uncharacterized protein OS=Sus scrofa OX=9823 GN=GGA3 PE=1 SV=2	69.298	647	A0A286ZN77;F1RVX3;A0A287BF21	1	1	2.2	2.2	0	NA	NA	1	0.001545595	7
A0A287BB68	MYH11	Myosin-11 OS=Sus scrofa OX=9823 GN=MYH11 PE=1 SV=1	197.323	1706	A0A287ASK1	1	1	0.8	0.8	0	NA	NA	1	0.000586166	11
F1RYI8	COL3A1	Collagen alpha-1(III) chain preproprotein OS=Sus scrofa OX=9823 GN=COL3A1 PE=1 SV=2	138.619	1466	A0A286ZQ85	1	1	0.8	0.8	19996000	NA	NA	1	0.000682128	8

F1SQ09	LUM	Lumican OS=Sus scrofa OX=9823 GN=LUM PE=1 SV=1	38.779	341		2	2	7.6	7.6	27386000	NA	NA	1	0.002932551	16
F1SR80	OC1001580	Tubulin alpha chain OS=Sus scrofa OX=9823 GN=LOC100158003 PE=3 SV=3	48.743	437	A0A5G2RB77;A0A5G2QX54;F1SHC1;F2Z5T5;Q2XVP4;P02550;F1RYV9	1	1	3.2	3.2	46110000	NA	NA	1	0.00228833	0
F2Z5L5	H2AC20	Histone H2A OS=Sus scrofa OX=9823 GN=H2AC20 PE=1 SV=1	13.988	129	F2Z5L6	1	1	8.5	8.5	3011300	NA	NA	1	0.007751938	17
A0A286ZY60	MBNL1	Uncharacterized protein OS=Sus scrofa OX=9823 GN=MBNL1 PE=4 SV=1	36.921	339	A0A286ZTG2;A0A287BJI1;F1SJM7;A0A480ZSM5;A0A286ZV49;A0A287AVV5;A0A287AT92;A0A287AH18	1	1	4.1	4.1	48777000	NA	NA	0	0	9
A0A287BCL3	HES5	Uncharacterized protein OS=Sus scrofa OX=9823 GN=HES5 PE=4 SV=2	24.717	226		1	1	6.2	6.2	1374400000	NA	NA	0	0	14
A0A5G2QKZ7	DCN	Decorin OS=Sus scrofa OX=9823 GN=DCN PE=3 SV=1	35.644	321	A0A5G2RB02;F1SQ10;Q9XSD9	1	1	5.9	5.9	0	NA	NA	0	0	4

Bovine pericardium

Accession	Gene	Description	Mw(kDa)	Length	Protein group	Peptides	Unique peptides	Sequence coverage [%]	Intensity	iBAQ	iBAQ [%]	LFQ intensity	MS/MS count	SAF	Protein group ID
A0A3Q1NA44	COL1A2	Collagen alpha-2(I) chain OS=Bos taurus OX=9913 GN=COL1A2 PE=1 SV=1	118.063	1248	A0A3Q1LZN8;P02465	33	33	5	23518000000	NA	NA	12738000000	303	0.242788462	5
F1N0H9	ELN	Elastin OS=Bos taurus OX=9913 GN=ELN PE=3 SV=3	60.533	711	A0A3Q1N0T9	73	0	51.2	11491000000	NA	NA	14880000000	87	0.122362869	21
P02453	COL1A1	Collagen alpha-1(I) chain OS=Bos taurus OX=9913 GN=COL1A1 PE=1 SV=3	138.938	1463	Q28083	26	25	6.8	9720700000	NA	NA	9510000000	44	0.030075188	4
A0A3Q1MFJ9	ELN	Elastin OS=Bos taurus OX=9913 GN=ELN PE=3 SV=1	63.455	740	A3Q1M7F0;A0A3Q1ME	71	4	50.8	348230000	NA	NA	521290000	5	0.006756757	20
E1BB91	COL6A3	Collagen type VI alpha 3 chain OS=Bos taurus OX=9913 GN=COL6A3 PE=1 SV=2	339.587	3132		15	15	4.9	239520000	NA	NA	307000000	13	0.004150702	26
A6QQQ3	PRELP	Prolargin OS=Bos taurus OX=9913 GN=PRELP PE=2 SV=1	43.701	381	Q9GKN8	3	3	5.5	136310000	NA	NA	134630000	3	0.007874016	17
E1BI98	COL6A1	Collagen type VI alpha 1 chain OS=Bos taurus OX=9913 GN=COL6A1 PE=1 SV=1	108.671	1027		4	4	4.9	134420000	NA	NA	117540000	5	0.004868549	28
A7E303	COL8A1	COL8A1 protein OS=Bos taurus OX=9913 GN=COL8A1 PE=2 SV=1	73.254	745		3	3	4.7	109830000	NA	NA	131030000	5	0.006711409	24
F1N401	COL12A1	Collagen alpha-1(XII) chain OS=Bos taurus OX=9913 GN=COL12A1 PE=4 SV=3	333.001	3065		2	2	0.4	98165000	NA	NA	141810000	2	0.000652529	30
A4IFU5	H2AW	Histone H2A OS=Bos taurus OX=9913 GN=H2AW PE=2 SV=1	14.121	130	N7K2;A0A3Q1MF62;F	1	1	6.2	87025000	NA	NA	0	0	0	18
P13605	FMOD	Fibromodulin OS=Bos taurus OX=9913 GN=FMOD PE=1 SV=2	43.038	376		3	3	7.7	76850000	NA	NA	111020000	2	0.005319149	7
G3MZI7	COL5A1	Collagen type V alpha 1 chain OS=Bos taurus OX=9913 GN=COL5A1 PE=4 SV=2	184.867	1847	1N0K0;F1MRP6;Q32S	8	7	2.8	72301000	NA	NA	136460000	3	0.001624256	31
P04985	ELN	Elastin OS=Bos taurus OX=9913 GN=ELN PE=1 SV=1	64.229	747		59	1	35.6	67750000	NA	NA	97876000	3	0.004016064	6
E1BGN3	HIST2H3D	Histone H3 OS=Bos taurus OX=9913 GN=HIST2H3D PE=3 SV=1	15.418	136	P68432;G3MWX6;A0A	2	2	8.8	59250000	NA	NA	97153000	1	0.007352941	13
F1MKG2	COL6A2	Collagen type VI alpha 2 chain OS=Bos taurus OX=9913 GN=COL6A2 PE=1 SV=3	105.178	979	Q1JQB0	6	6	5.1	44492000	NA	NA	0	5	0.005107252	29

P21793	DCN	Decorin OS=Bos taurus OX=9913 GN=DCN PE=1 SV=2	39.879	360		2	2	5	9522900	NA	NA	0	1	0.002777778	9
A0A3Q1MNL9	TGFBI	Transforming growth factor-beta-induced protein ig-h3 OS=Bos taurus OX=9913 GN=TGFBI PE=4 SV=1	70.2	642	F1MBS3;P55906	2	2	2.2	8763900	NA	NA	11007000	2	0.003115265	10
E1BA17	COL14A1	Collagen type XIV alpha 1 chain OS=Bos taurus OX=9913 GN=COL14A1 PE=1 SV=2	193.775	1800	A0A3Q1N775	1	1	1.1	952780	NA	NA	1490800	0	0	25
G5E5Y4		Uncharacterized protein OS=Bos taurus OX=9913 PE=4 SV=2	164.998	1475	A0A3Q1NFN9	1	1	1.1	0	NA	NA	0	0	0	22
G5E6E9	LOC789812	Olfactory receptor OS=Bos taurus OX=9913 GN=LOC789812 PE=3 SV=2	36.128	317		1	1	4.1	0	NA	NA	0	0	0	32
A0A3Q1LT99	ANO3	Anoctamin OS=Bos taurus OX=9913 GN=ANO3 PE=3 SV=1	105.051	892	N7W6;F1MHG6;A0A3(	1	1	0.9	0	NA	NA	0	0	0	19
A0A452DJE4	ACTA2	Actin, aortic smooth muscle OS=Bos taurus OX=9913 GN=ACTA2 PE=3 SV=1	37.628	340	;;Q3ZC07;P68138;P62	1	1	2.9	0	NA	NA	0	1	0.002941176	11

Bovine dermis

Accession	Gene	Description	Mw(kDa)	Length	Protein group	Peptides	Unique peptides	Sequence coverage [%]	Intensity	iBAQ	iBAQ [%]	LFQ intensity	MS/MS count	SAF	Protein group ID
A0A3Q1NA44	COL1A2	Collagen alpha-2(I) chain OS=Bos taurus OX=9913 GN=COL1A2 PE=1 SV=1	118.063	1248	A0A3Q1LZN8;P02465	40	40	13.2	26446000000	NA	NA	13263000000	156	0.125	5
P02453	COL1A1	Collagen alpha-1(I) chain OS=Bos taurus OX=9913 GN=COL1A1 PE=1 SV=3	138.938	1463	Q28083	67	66	11.8	23730000000	NA	NA	19628000000	100	0.0683527	4
F1N0H9	ELN	Elastin OS=Bos taurus OX=9913 GN=ELN PE=3 SV=3	60.533	711	A0A3Q1N0T9	28	0	30.4	1152600000	NA	NA	1946200000	24	0.033755274	21
E1BB91	COL6A3	Collagen type VI alpha 3 chain OS=Bos taurus OX=9913 GN=COL6A3 PE=1 SV=2	339.587	3132		62	62	18.4	1971200000	NA	NA	1845700000	62	0.019795658	26
E1BI98	COL6A1	Collagen type VI alpha 1 chain OS=Bos taurus OX=9913 GN=COL6A1 PE=1 SV=1	108.671	1027		20	20	19.4	1380900000	NA	NA	1385500000	20	0.019474197	28
F1MKG2	COL6A2	Collagen type VI alpha 2 chain OS=Bos taurus OX=9913 GN=COL6A2 PE=1 SV=3	105.178	979	Q1JQB0	19	19	21.7	520080000	NA	NA	485860000	16	0.016343207	29
G3MZI7	COL5A1	Collagen type V alpha 1 chain OS=Bos taurus OX=9913 GN=COL5A1 PE=4 SV=2	184.867	1847	F1N0K0;F1MRP6;Q32S24	8	7	2.9	356780000	NA	NA	191350000	15	0.008121278	31
E1BA17	COL14A1	Collagen type XIV alpha 1 chain OS=Bos taurus OX=9913 GN=COL14A1 PE=1 SV=2	193.775	1800	A0A3Q1N775	8	8	5.9	89257000	NA	NA	89142000	7	0.003888889	25
E1BGN3	HIST2H3D	Histone H3 OS=Bos taurus OX=9913 GN=HIST2H3D PE=3 SV=1	15.418	136	A0A3Q1MPB8;A0A3Q1M588;Q5E9F8;P84227;P68432;G3MWX6;A0A3Q1LMR9;G3N2P2;G3MYD7;Q3SZB8;A5PK61	4	4	25.7	829720000	NA	NA	771520000	6	0.044117647	13
A0A3Q1MFJ9	ELN	Elastin OS=Bos taurus OX=9913 GN=ELN PE=3 SV=1	63.455	740	A0A3Q1M7F0;A0A3Q1MEV3	29	2	30.8	54904000	NA	NA	36673000	1	0.001351351	20
A4IFU5	H2AW	Histone H2A OS=Bos taurus OX=9913 GN=H2AW PE=2 SV=1	14.121	130	F1MLQ1;F2Z4G5;F2Z4J1;A0A0A0MP90;P0C0S9;A0A0A0MP93;E1BH22;A0A096LNF2;A0A3Q1N7K2;A0A3Q1MF62;F1MRN2;F2Z4I6;A0A3Q1MDK7;Q3ZBX9;A1A4R1;A0A3Q1N0B0;P0C0S4;Q32LA7;A0A3Q1M0K4	5	5	39.2	308960000	NA	NA	308960000	4	0.030769231	18
A5D9E8	OGN	Mimecan OS=Bos taurus OX=9913 GN=OGN PE=2 SV=1	34.196	299	P19879	3	3	4.7	97850000	NA	NA	80713000	4	0.013377926	8
A6QQQ3	PRELP	Prolargin OS=Bos taurus OX=9913 GN=PRELP PE=2 SV=1	43.701	381	Q9GKN8	1	1	2.9	35977000	NA	NA	54038000	1	0.002624672	17
P04985	ELN	Elastin OS=Bos taurus OX=9913 GN=ELN PE=1 SV=1	64.229	747		24	1	22.2	24656000	NA	NA	24656000	1	0.001338688	6
P21793	DCN	Decorin OS=Bos taurus OX=9913 GN=DCN PE=1 SV=2	39.879	360		5	5	15	290000000	NA	NA	290000000	3	0.008333333	9

A0A3Q1MNL9	TGFBI	Transforming growth factor-beta-induced protein ig-h3 OS=Bos taurus OX=9913 GN=TGFBI PE=4 SV=1	70.2	642	F1MBS3;P55906	1	1	2	3889900	NA	NA	3889900	1	0.001557632	10
A0A452DJE4	ACTA2	Actin, aortic smooth muscle OS=Bos taurus OX=9913 GN=ACTA2 PE=3 SV=1	37.628	340	G8JKX4;P63258;P60712;Q5E9B5;Q3ZC07;P68138;P62739;A0A3Q1NKP5;A0A3Q1M558	2	2	8.2	82708000	NA	NA	82708000	2	0.005882353	11
F1N401	COL12A1	Collagen alpha-1(XII) chain OS=Bos taurus OX=9913 GN=COL12A1 PE=4 SV=3	333.001	3065		1	1	0.5	10282000	NA	NA	0	1	0.000326264	30
Q3ZBN5	ASPN	Asporin OS=Bos taurus OX=9913 GN=ASPN PE=2 SV=1	42.119	370		2	2	6.8	180130000	NA	NA	180130000	3	0.008108108	16
E1B9F6		Elongation factor 1-alpha OS=Bos taurus OX=9913 PE=3 SV=3	48.049	440	P68103	1	1	3	16853000	NA	NA	16853000	1	0.002272727	12
F1MNF8	.OC10014126€	Tubulin alpha chain OS=Bos taurus OX=9913 GN=LOC100141266 PE=3 SV=1	49.902	449	A0A452DJ66;A0A3Q1LQR2;Q3ZCJ7;F2Z4C1;P81947;Q2HJ86	1	1	3.1	15910000	NA	NA	15910000	1	0.002227171	14
A0A3Q1LT99	ANO3	Anoctamin OS=Bos taurus OX=9913 GN=ANO3 PE=3 SV=1	105.051	892	A0A3Q1N7W6;F1MHG6;A0A3Q1MUE0	1	1	0.9	0	NA	NA	0	0	0	19
A6QR41	C8H9orf72	Chromosome 8 C9orf72 homolog OS=Bos taurus OX=9913 GN=C8H9orf72 PE=2 SV=1	54.296	481		1	1	2.7	0	NA	NA	0	0	0	23
E1BGY1	FAM111B	Family with sequence similarity 111 member B OS=Bos taurus OX=9913 GN=FAM111B PE=4 SV=2	100.011	874		1	1	1.6	0	NA	NA	0	0	0	27
