# Supplementary material for: Physical, mechanical, and biological properties of collagen membranes for guided bone regeneration: a comparative in vitro study
Source: BMC Oral Health. 2023 Jul 22;23:510. doi: 10.1186/s12903-023-03223-4 (PMC10362553; doi:10.1186/s12903-023-03223-4)
Supplement: Supplementary file 2 — Additional file 2: Supplementary Table S2. Identified peptides. [file 12903_2023_3223_MOESM2_ESM.pdf]

Porcine dermis

Accession	Unique pr	Sequence	Length	Count	Modification	Modified sequence	Missed cleavage	Type	Charge	m/z	Mass	ppm	Retention	Retention l	Retention time	PEP	MS/MS co	Score	Intensity	Protein group ID	Peptide ID	Evidence ID
F1SFA7	yes	GYEGDFYR	8	2	Unmodified	_GYEGDFYR_		MULTI-MSMS	2	503.716882	1005.41921	-0.14591	22.852	0.69591	0	3.4065E-07	1	138.62	30489000	15	136	262
A0A287AXU0	no	VLPGTGAR	8	2	Unmodified	_VLPGTGAR_		MULTI-MSMS	2	385.729595	769.444637	-0.37448	8.5094	0.68308	0	0.0053072	1	124.34	200530000	12	219	370
F1SFA7	yes	FGYEGDFYR	9	11	Unmodified	_FGYEGDFYR_		MULTI-MSMS	2	577.251089	1152.48762	1.8436	36.68	0.69669	0	1.7131E-08	1	169.06	22316000	15	18	28
A0A287AXU0	no	GGVGDLGGA	9	11	Unmodified	_GGVGDLGGA_		MULTI-MSMS	1	702.341694	701.334418	-3.8237	18.649	0.70847	0	0.00036083	1	128.38	4203300	12	42	70
A0A287AXU0	no	GGVLPGTGA	9	11	Unmodified	_GGVLPGTGA_		MULTI-MSMS	1	728.39373	727.386454	1.8208	23.735	0.74017	0	0.00033974	1	128.75	78268000	12	49	78
A0A287BLD2	no	GPPSGGFDF	9	11	Unmodified	_GPPSGGFDF_		MULTI-MSMS	2	440.695418	879.376283	-0.83343	47.354	1.7296	0	7.9813E-07	2	159.19	24354000	10	66	103
A0A287AXU0	no	GVLPGTGAR	9	11	Unmodified	_GVLPGTGAR_		MULTI-MSMS	2	414.240327	826.466101	-0.76781	12.509	1.4961	0	0.0089144	1	122.13	1085900000	12	112	227
F1SFA7	yes	GYEGDFYRA	9	11	Unmodified	_GYEGDFYRA_		MULTI-MSMS	2	539.235439	1076.45632	0.34047	28.554	0.6853	0	0.013496	1	120.49	27149000	15	137	263
A0A287AXU0	no	IPTFGVGAG	9	11	Unmodified	_IPTFGVGAG_		MULTI-MSMS	1	818.44068	817.433404	0.80635	45.488	0.4596	0	1.1135E-06	1	143.02	8715700	12	141	269
A0A287AXU0	no	STGAVVPQL	9	11	Unmodified	_STGAVVPQL_		MULTI-MSMS	2	436.247818	870.481082	0.54288	40.467	0.84734	0	0.016099	1	119.56	13841000	12	178	323
A0A287AXU0	no	VGGLGVGGL	9	11	Unmodified	_VGGLGVGGL_		MULTI-MSMS	1	728.430116	727.422839	0.75214	44.694	0.46075	0	0.025697	1	116.12	10972000	12	191	339
A0A287AXU0	no	VLGVTRPFP	9	11	Unmodified	_VLGVTRPFP_		MULTI-MSMS	2	493.295102	984.575651	0.28454	39.92	3.3074	0	0.010338	1	121.62	103810000	12	216	367
A0A5G2QY90	no	VLSLYASGR	9	11	Unmodified	_VLSLYASGR_		MULTI-MSMS	2	483.274367	964.53418	1.5413	21.79	0.53459	0	0.025338	1	116.25	6372200	2	222	373
F1SFA7	yes	DFGYEGDFYR	10	14	Unmodified	_DFGYEGDFYR_		MULTI-MSMS	2	634.76456	1267.51457	0.29004	46.198	1.2896	0	2.3717E-18	2	196.95	170620000	15	9	9
A0A287BLD2	no	DFSFLPQPPQ	10	14	Unmodified	_DFSFLPQPPQ_		MULTI-MSMS	2	588.290214	1174.56587	0.60596	74.328	1.2072	0	0.00049357	1	124.83	12930000	10	10	15
A0A287AXU0	no	FPLGGVAPRP	10	14	Unmodified	_FPLGGVAPRP_		MULTI-MSMS	2	505.792727	1009.5709	-0.0066653	34.95	1.5667	-7.1054E-15	0.026113	1	100.45	127930000	12	20	31
A0A287BLD2	no	GPPSGGFDFS	10	14	Unmodified	_GPPSGGFDFS_		MULTI-MSMS	2	484.211432	966.408311	-0.38772	42.985	2.211	-7.1054E-15	2.36E-18	5	196.98	553990000	10	67	108
A0A287AXU0	no	GVAPRPGFGL	10	14	Unmodified	_GVAPRPGFGL_		MULTI-MSMS	2	485.777077	969.5396	-0.014573	31.999	0.71821	-3.5527E-15	0.016129	1	107.32	54729000	12	89	203
A0A287AXU0	no	GVLGVTRPFP	10	14	Unmodified	_GVLGVTRPFP_		MULTI-MSMS	2	521.805834	1041.59712	-1.3132	42.968	3.6407	0	0.000070562	5	131.06	111940000	12	110	225
A0A287AXU0	no	GVLPGVPTGT	10	14	Unmodified	_GVLPGVPTGT_		MULTI-MSMS	2	449.255643	896.496732	-1.9192	47.546	1.0661	0	0.016688	1	106.42	40548000	12	114	229
A0A287AXU0	no	GVSTGAVVPQ	10	14	Unmodified	_GVSTGAVVPQ_		MULTI-MSMS	2	457.750724	913.486896	0.73984	20.932	0.8569	3.5527E-15	0.024715	1	101.28	96660000	12	130	248
A0A287AXU0	no	GVTRPFPLGG	10	14	Unmodified	_GVTRPFPLGG_		MULTI-MSMS	2	500.782359	999.550165	-0.21493	30.978	1.5566	0	0.019425	1	104.44	232700000	12	132	252
A0A287B5M9	no	HYSDEVEIIR	10	14	Unmodified	_HYSDEVEIIR_		MULTI-MSMS	2	630.814584	1259.61462	0.77601	21.96	0.42376	0	0.000029916	1	133.57	8634600	13	138	265
A0A287AXU0	no	VAPGIGIGPG	10	14	Unmodified	_VAPGIGIGPG_		MULTI-MSMS	2	419.245078	836.475603	-0.38105	36.124	2.523	0	0.027691	1	99.511	3054300000	12	182	330
A0A287AXU0	no	VLGVTRPFPL	10	14	Unmodified	_VLGVTRPFPL_		MULTI-MSMS	2	549.837134	1097.65972	0.83715	55.014	0.57924	0	0.020891	1	103.56	8740100	12	217	368
A0A287AXU0	no	VLPGVPTGTG	10	14	Unmodified	_VLPGVPTGTG_		MULTI-MSMS	2	449.255643	896.496732	-0.06565	35.3	1.1572	0	0.01668	1	106.43	269310000	12	221	372
A0A287AXU0	no	YPGGVLPGTG	10	14	Unmodified	_YPGGVLPGTG_		MULTI-MSMS	2	459.239993	916.465432	0.48794	38.384	1.5511	0	0.024837	1	101.21	316810000	12	230	386
A0A287BLD2	no	DFSFLPQPPQE	11	21	Unmodified	_DFSFLPQPPQE_		MULTI-MSMS	2	652.81151	1303.60847	-0.20776	75.072	1.5891	0	5.3774E-06	1	169.65	41379000	10	11	16
A0A287AXU0	no	FPLGGVAPRPG	11	21	Unmodified	_FPLGGVAPRPG_		MULTI-MSMS	2	534.303458	1066.59236	-0.63814	32.744	0.75302	0	0.024944	2	98.523	16263000	12	21	32
A0A287AXU0	no	GAGLGGLGGGA	11	21	Unmodified	_GAGLGGLGGGA_		MULTI-MSMS	1	786.410443	785.403166	0.2491	26.446	0.53933	3.5527E-15	0.0081314	1	111.69	10126000	12	26	39
A0A287AXU0	no	GGVLGVTRPFP	11	21	Unmodified	_GGVLGVTRPFP_		MULTI-MSMS	2	550.316566	1098.61858	-0.3801	43.121	3.0931	0	0.00013837	5	132.76	380130000	12	46	74
A0A287AXU0	no	GPFGGQQPGVP	11	21	Unmodified	_GPFGGQQPGVP_		MULTI-MSMS	2	520.761623	1039.50869	-0.35456	38.048	1.8871	0	0.00054223	2	124.37	320930000	12	57	91
F1SQ09	yes	GPLPKSLVDLQ	11	21	Unmodified	_GPLPKSLVDLQ_		MULTI-MSMS	2	583.842613	1165.67067	-0.95647	45.151	0.62829	0	0.022992	1	99.815	27386000	16	65	102
A0A287BLD2	no	GPPSGGFDFSF	11	21	Unmodified	_GPPSGGFDFSF_		MULTI-MSMS	2	557.745639	1113.47673	0.32872	73.02	1.8504	0	5.617E-15	3	199.37	20771000	10	68	113
A0A287AXU0	no	GVLPGVPTGTG	11	21	Unmodified	_GVLPGVPTGTG_		MULTI-MSMS	2	477.766374	953.518196	-0.14382	45.802	1.1084	0	0.00017302	2	129.39	318850000	12	115	230
A0A287AXU0	no	GVPGVGVPGVG	11	21	Unmodified	_GVPGVGVPGVG_		MULTI-MSMS	2	447.75581	893.497067	-1.2165	45.579	1.9877	0	0.012968	1	106.76	307930000	12	116	232
A0A287AXU0	no	GVPGVGVPGVP	11	21	Unmodified	_GVPGVGVPGVP_		MULTI-MSMS	2	467.77146	933.528367	1.3508	50.906	1.5005	0	0.012968	1	106.76	29441000	12	128	246
A0A287BLD2	no	LDGAKGDAGPA	11	21	Unmodified	_LDGAKGDAGPA_		MULTI-MSMS	2	486.243264	970.471974	-0.94168	7.702	0.19231	0	0.0029983	1	118.71	14596000	10	143	271
A0A287AXU0	no	LGGVAPRPGFG	11	21	Unmodified	_LGGVAPRPGFG_		MULTI-MSMS	2	514.287808	1026.56106	-0.17214	24.237	0.43711	0	0.02203	1	100.45	41965000	12	144	272
A0A0H5ANC0	yes	LLEELILADNQ	11	21	Unmodified	_LLEELILADNQ_		MULTI-MSMS	2	635.848093	1269.68163	-0.57288	75.35	0.58575	0	0.0035374	1	146.58	10329000	5	147	275
A0A287AXU0	no	PFPLGGVAPRP	11	21	Unmodified	_PFPLGGVAPRP_		MULTI-MSMS	2	554.319109	1106.62366	1.1945	40.295	0.85259	7.1054E-15	0.00012773	2	133.79	38409000	12	153	282
A0A287AXU0	no	PGGVLPGTGAR	11	21	Unmodified	_PGGVLPGTGAR_		MULTI-MSMS	2	491.277441	980.540328	-1.4501	13.423	1.4946	0	0.00060435	2	147.51	1005600000	12	154	284
A0A287AXU0	no	VAPGIGIGPGG	11	21	Unmodified	_VAPGIGIGPGG_		MULTI-MSMS	2	447.75581	893.497067	0.66371	36.405	0.7861	0	0.012968	1	106.76	62637000	12	183	331
A0A287AXU0	no	VGVLPGVPTGT	11	21	Unmodified	_VGVLPGVPTGT_		MULTI-MSMS	2	498.78985	995.565146	0.0096143	52.736	0.8914	-7.1054E-15	0.016807	1	103.91	58160000	12	206	356
F2Z5L5	no	VLLPKKTESHK	11	21	Unmodified	_VLLPKKTESHK_		MULTI-MSMS	3	427.2626	1278.76597	1.11	7.0469	0.248	0	0.0040734	1	116.23	3011300	17	218	369
A0A287AXU0	no	VYPGGVLPGTG	11	21	Unmodified	_VYPGGVLPGTG_		MULTI-MSMS	2	508.7742	1015.53385	1.1787	43.771	1.114	0	0.012422	1	107.32	40220000	12	227	378
F1SFA7	yes	YDFGYEGDFYR	11	21	Unmodified	_YDFGYEGDFYR_		MULTI-MSMS	2	716.296224	1430.5779	0.98593	47.85	0.75752	-7.1054E-15	0.00014179	2	162.88	160740000	15	229	381
A0A287AXU0	no	YPGGVLPGTGA	11	21	Unmodified	_YPGGVLPGTGA_		MULTI-MSMS	2	494.758549	987.502546	-1.2502	39.726	1.9723	0	0.0080925	2	111.73	1452700000	12	231	388
A0A287AFS2	no	ADFTEISKIVTD	12	33	Unmodified	_ADFTEISKIVTD_		MULTI-MSMS	2	669.843007	1337.67146	1.5408	58.069	0.73058	0	3.3871E-08	1	176.6	45523000	1	0	0
A0A287BLD2	no	FDFSFLPQPPQE	12	33	Unmodified	_FDFSFLPQPPQE_		MULTI-MSMS	2	726.345717	1450.67688	-3.5661	80.025	0.69742	0	0.016201	1	116.52	33602000	10	15	23
A0A287AFS2	no	GEYGFQNALIVR	12	33	Unmodified	_GEYGFQNALIVR_		MULTI-MSMS	2	683.859326	1365.7041	0.4591	46.829	0.60005	-7.1054E-15	0.016115	1	141.48	25670000	1	31	45
A0A287AXU0	no	GGQQPGVPLGYP	12	33	Unmodified	_GGQQPGVPLGYP_		MULTI-MSMS	2	585.301113	1168.58767	1.2439	44.559	1.6835	0	0.020522	1	110.81	215600000	12	41	69
A0A287AXU0	no	GGVLGVTRPFPL	12	33	Unmodified	_GGVLGVTRPFPL_		MULTI-MSMS	2	606.858598	1211.70264	1.1182	58.042	0.85744	7.1054E-15	0.0033288	2	123.86	70441000	12	47	76
F1SFA7	yes	GLAGHHGDQGAP	12	33	Unmodified	_GLAGHHGDQGAP_		MULTI-MSMS	2	558.762686	1115.51082	-0.60272	7.2491	0.21405	0	0.0042005	1	136.27	18082000	15	52	85
A0A287BLD2	no	GLDGAKGDAGPA	12	33	Unmodified	_GLDGAKGDAGPA_		MULTI-MSMS	2	514.753995	1027.49344	0.4228	8.8823	0.2991	0	0.0035242	1	123.75	11361000	10	54	87
A0A287AXU0	no	GLGVSTGAVVPQ	12	33	Unmodified	_GLGVSTGAVVPQ_		MULTI-MSMS	2	542.803488	1083.59242	0.2665	41.255	1.0924	0	0.019865	1	143.12	36968000	12	55	89
A0A287AXU0	no	GPFGGQQPGVPL	12	33	Unmodified	_GPFGGQQPGVPL_		MULTI-MSMS	2	577.303655	1152.59276	-0.42582	54.634	1.2849	0	0.015074	1	117.16	143620000	12	58	93
F1RYI8	no	GPGGISGITGAR	12	33	Unmodified	_GPGGISGITGAR_		MULTI-MSMS	2	521.78563	1041.55671	-2.1621	16.216	0.48445	0	0.021139	1	109.66	19996000	8	60	96
A0A287BLD2	no	GPPSGGFDFSFL	12	33	Unmodified	_GPPSGGFDFSFL_		MULTI-MSMS	2	614.287671	1226.56079	0.1544	80.203	0.68686	1.4211E-14	0.00080049	2	155.75	80708000	10	69	114
F1SFA7	yes	GPVGRTGETGAS	12	33	Unmodified	_GPVGRTGETGAS_		MULTI-SECPEP	2	544.770177	1087.5258	-3.0748	7.3435	0.44854	8.8818E-16	5.3355E-69	1	198.25	128330000	15	86	200
A0A287AXU0	no	GVGVLPGVPTGT	12	33	Unmodified	_GVGVLPGVPTGT_		MULTI-MSMS	2	527.300581	1052.58661	-0.86645	54.769	0.78972	0	0.021032	1	109.86	48504000	12	99	214
A0A287AXU0	no	GVGVPGVGVPGV	12	33	Unmodified	_GVGVPGVGVPGV_		MULTI-MSMS	2	497.290017	992.565481	-0.227	58.475	1.8672	7.1054E-15	0.020268	2	111.28	538840000	12	103	218
A0A287AXU0	no	GVPGVGVPGVGV	12	33	Unmodified	_GVPGVGVPGVGV_		MULTI-MSMS	2	497.290017	992.565481	-0.16884	56.106	0.60215	0	0.018788	1	114.02	12524000	12	117	233
A0A287AXU0	no	GVPGVGVPGVPG	12	33	Unmodified	_GVPGVGVPGVPG_		MULTI-SECPEP	1	991.557107	990.549831	1.2786	78.727	0.35005	0	0.000025068	1	125.86	1258100	12	129	247
F1SFA7	yes	GYDFGYEGDFYR	12	33	Unmodified	_GYDFGYEGDFYR_		MULTI-MSMS	2	744.806956	1487.59936	1.2483	48.709	0.98722	0	0.0010717	1	126.2	20223000	15	135	255
A0A287AXU0	no	PGGVPGGVFFPG	12	33	Unmodified	_PGGVPGGVFFPG_		MULTI-MSMS	1	1087.55711	1086.54983	0.9726	66.856	0.50238	0	0.024805	1	104.11	3262300	12	156	288
A0A287AXU0	no	PLGGVAPRPGFG	12	33	Unmodified	_PLGGVAPRPGFG_		MULTI-MSMS	2	562.81419	1123.61383	-0.78932	29.288	0.889	0	0.0029446	1	124.08	104670000	12	164	296
A0A287BLD2	no	PPSGGFDFSFLP	12	33	Unmodified	_PPSGGFDFSFLP_		MULTI-MSMS	2	634.303321	1266.59209	-0.79882	78.849	0.51632	0	0.00088108	1	131.06	23982000	10	165	297
A0A287AXU0	no	RPFPLGGVAPRP	12	33	Unmodified	_RPFPLGGVAPRP_		MULTI-MSMS	3	421.915535	1262.72478	-0.17588	27.46	0.89532	0	0.0029022	1	124.1	16525000	12	171	309
A0A5G2QMD8	no	SPPLGPIPPVHP	12	33	Unmodified	_SPPLGPIPPVHP_		MULTI-MSMS	2	604.345323	1206.67609	-0.73131	44.878	1.72	-7.1054E-15	3.3594E-08	4	176.65	22009000	3	177	322
A0A287AXU0	no	STGAVVPQLGAG	12	33	Unmodified	_STGAVVPQLGAG_		MULTI-MSMS	2	528.787838	1055.56112	-0.15541	37.641	1.4352	0	0.0031128	2	123.98	576680000	12	179	324
A0A287AFS2	no	TEISKIVTDLAK	12	33	Unmodified	_TEISKIVTDLAK_		MULTI-MSMS	2	659.384842	1316.75513	0.79389	40.757	0.71376	0	9.4224E-14	2	196.11	81690000	1	181	329
A0A287BLM4	no	VDSLDVGFDTSR	12	33	Unmodified	_VDSLDVGFDTSR_		MULTI-MSMS	2	655.814781	1309.61501	2.448	39.034	0.43384	0	0.0067674	1	121.9	8755200	6	188	336
A0A287AXU0	no	VGGIPTFGVGAG	12	33	Unmodified	_VGGIPTFGVGAG_		MULTI-MSMS	2	516.279649	1030.54475	0.54348	59.576	0.60708	7.1054E-15	0.0010991	1	125.5	9351500	12	190	338
A0A287AXU0	no	VGGVPGAALSPA	12	33	Unmodified	_VGGVPGAALSPA_		MULTI-MSMS	2	498.279649	994.544745	1.2908	40.82	0.86864	0	0.026094	1	102.71	46754000	12	193	341
A0A287AXU0	no	VGPFGGQQPGVP	12	33	Unmodified	_VGPFGGQQPGVP_		MULTI-MSMS	2	570.29583	1138.57711	-0.12722	47.448	5.8029	7.1054E-15	0.00092121	6	130.04	7800500000	12	197	345
A0A287AXU0	no	VGVLPGVPTGTG	12	33	Unmodified	_VGVLPGVPTGTG_		MULTI-MSMS	2	527.300581	1052.58661	0.53503	51.046	0.80499	0	0.013512	2	118.05	393300000	12	207	357

A0A287AXU0	no	VGVPGVGVPGVG	12	33	Unmodified	_VGVPGVGVPGVG_	MULTI-MSMS	2	497.290017	992.565481	-1.2416	54.563	2.0333	0	0.024805	2	104.11	3359800000	12	208	358
A0A287AXU0	no	VPGAGAFGGVSP	12	33	Unmodified	_VPGAGAFGGVSP_	MULTI-MSMS	1	1015.52072	1014.51345	0.62369	45.795	0.58172	0	0.02149	1	109.01	7014400	12	223	374
A0A287AXU0	no	VYPGGVLPGTGA	12	33	Unmodified	_VYPGGVLPGTGA_	MULTI-MSMS	1	1087.57824	1086.57096	1.2084	44.971	0.79288	0	0.0052888	1	122.74	25643000	12	228	379
A0A287AXU0	no	YPGGVLPGTGAR	12	33	Unmodified	_YPGGVLPGTGAR_	MULTI-MSMS	2	572.809105	1143.60366	-1.7695	24.423	1.9608	0	0.0007998	3	133.13	7657100000	12	232	390
A0A287BLD2	no	FDFSFLPQPPQEK	13	20	Unmodified	_FDFSFLPQPPQEK_	MULTI-MSMS	2	790.393199	1578.77184	0.079108	66.108	1.4025	0	0.00279	1	133.89	28944000	10	16	25
A0A287AXU0	no	FPLGGVAPRPGFG	13	20	Unmodified	_FPLGGVAPRPGFG_	MULTI-MSMS	2	636.348397	1270.68224	0.42964	49.315	1.4449	-7.1054E-15	0.013879	2	151.04	186690000	12	22	33
A0A287BLD2	no	GFDFSFLPQPPQE	13	20	Unmodified	_GFDFSFLPQPPQE_	MULTI-MSMS	2	754.856449	1507.69835	0.64643	79.869	1.0089	0	0.00458	1	131.45	23246000	10	32	46
F1SFA7	yes	GGGYDFGYEGDFY	13	20	Unmodified	_GGGYDFGYEGDFY_	MULTI-SECPEP	2	723.777864	1445.54118	-0.11443	67.615	1.5415	0	0.025009	1	81.548	54819000	15	39	56
F1SFA7	yes	GGYDFGYEGDFYR	13	20	Unmodified	_GGYDFGYEGDFYR_	MULTI-MSMS	2	773.317688	1544.62082	-0.082956	53.054	0.92349	0	1.0101E-07	1	188.87	37626000	15	51	81
A0A287AXU0	no	GLGVSTGAVVPQL	13	20	Unmodified	_GLGVSTGAVVPQL_	MULTI-MSMS	2	599.34552	1196.67649	0.64395	64.741	0.50877	0	0.015937	1	139.97	14536000	12	56	90
A0A287BLD2	no	GPPSGGFDFSFLP	13	20	Unmodified	_GPPSGGFDFSFLP_	MULTI-MSMS	2	662.814053	1323.61355	0.30099	80.632	1.6329	1.4211E-14	3.6282E-11	4	194.2	1909400000	10	70	118
A0A287AXU0	no	GVAPGVGVAPGVG	13	20	Unmodified	_GVAPGVGVAPGVG_	MULTI-MSMS	2	518.792924	1035.57129	-0.27939	38.881	0.76076	0	0.027399	1	114.72	107040000	12	87	201
A0A287AXU0	no	GVGVGGIPTFGVG	13	20	Unmodified	_GVGVGGIPTFGVG_	MULTI-SECPEP	1	1116.60479	1115.59751	0.38102	69.168	0.32405	0	0.023549	1	69.825	5141100	12	96	211
A0A287AXU0	no	GVGVLPGVPTGTG	13	20	Unmodified	_GVGVLPGVPTGTG_	MULTI-MSMS	2	555.811313	1109.60807	0.25853	53.294	1.2967	-7.1054E-15	0.0082268	1	153.78	286430000	12	100	215
A0A287AXU0	no	GVGVPGVGVPGVG	13	20	Unmodified	_GVGVPGVGVPGVG_	MULTI-SECPEP	2	525.800749	1049.58694	-0.98011	56.595	2.5859	-7.1054E-15	0.026705	1	82.305	1631500000	12	104	219
A0A287AXU0	no	GVLGVTRPFPLGG	13	20	Unmodified	_GVLGVTRPFPLGG_	MULTI-MSMS	2	635.36933	1268.72411	-0.81553	56.02	0.66752	0	0.00458	1	131.45	43318000	12	111	226
A0A287AXU0	no	GVPGVGVPGVGVP	13	20	Unmodified	_GVPGVGVPGVGVP_	MULTI-MSMS	2	545.816399	1089.61824	-0.33351	60.641	2.6956	0	0.0037368	3	135.35	1240300000	12	118	234
A0A287AXU0	no	GVYPGGVLPGTGA	13	20	Unmodified	_GVYPGGVLPGTGA_	MULTI-MSMS	2	572.803488	1143.59242	0.048375	50.062	0.91128	0	0.014722	1	122.18	96907000	12	133	253
A0A287AXU0	no	PGGVLPGTGARFP	13	20	Unmodified	_PGGVLPGTGARFP_	MULTI-SECPEP	2	613.33803	1224.66151	-0.77119	39.639	0.84097	0	0.019341	1	72.643	8796400	12	155	286
A0A287BLD2	no	PQPPQEKAHDGGR	13	20	Deamidation (	_PQ(de)PPQEKAHDGGR_	MULTI-MSMS	2	709.344572	1416.67459	-3.6375	56.473	0.57093	0	0.018907	1	141.1	6338900	10	168	306
A0A287BLD2	no	VGPAGKDGEAGAQ	13	20	Unmodified	_VGPAGKDGEAGAQ_	MSMS	2	578.783284	1155.55202	NaN	7.4487	1	0	0.0068759	1	103.31		10	196	344
A0A287AXU0	no	VGPFGGQQPGVPL	13	20	Unmodified	_VGPFGGQQPGVPL_	MULTI-MSMS	2	626.837862	1251.66117	1.0592	63.215	1.7024	7.1054E-15	0.0037633	2	132.57	843750000	12	198	348
A0A287BLM4	no	VIDELDVKPDGTR	13	20	Unmodified	_VIDELDVKPDGTR_	MULTI-MSMS	3	486.259584	1455.75692	0.42445	23.032	0.65772	0	0.0022088	1	134.68	25989000	6	215	366
A0A287AXU0	no	VSTGAVVPQLGAG	13	20	Unmodified	_VSTGAVVPQLGAG_	MULTI-MSMS	2	578.322045	1154.62954	-0.80515	42.972	0.53856	0	0.0277	1	113.66	49639000	12	226	377
A0A287BB68	no	ALEEALEAKEELER	14	30	Unmodified	_ALEEALEAKEELER_	MSMS	3	543.949187	1628.82573	NaN	34.056	1	0	0.012939	1	93.823		11	4	4
A0A287BCL3	yes	ATPAKEPKAPGTAP	14	30	Unmodified	_ATPAKEPKAPGTAP_	MULTI-SECPEP	2	668.366984	1334.71942	-0.13335	57.688	1.0915	7.1054E-15	0.027182	1	74.962	1374400000	14	8	8
A0A287BLD2	no	DFSFLPQPPQEKAH	14	30	Unmodified	_DFSFLPQPPQEKAH_	MSMS	2	820.907005	1639.79946	NaN	44.495	1	0	0.020193	1	58.37		10	12	18
A0A287BLD2	no	FSGLDGAKGDAGPA	14	30	Unmodified	_FSGLDGAKGDAGPA_	MULTI-MSMS	2	631.804217	1261.59388	1.6335	21.25	0.91195	0	0.000028472	1	179.94	41706000	10	23	34
F1SFA7	yes	GGGYDFGYEGDFYR	14	30	Unmodified	_GGGYDFGYEGDFYR_	MULTI-MSMS	2	801.82842	1601.64229	0.10542	52.317	0.97417	0	3.352E-44	2	235.32	252270000	15	40	61
A0A287AXU0	no	GGVGVGGIPTFGVG	14	30	Unmodified	_GGVGVGGIPTFGVG_	MULTI-MSMS	2	587.316763	1172.61897	0.4747	70.121	1.3735	1.4211E-14	0.025065	2	93.162	330010000	12	44	72
A0A287AXU0	no	GGVLGVTRPFPLGG	14	30	Unmodified	_GGVLGVTRPFPLGG_	MULTI-MSMS	2	663.880062	1325.74557	-0.42711	56.218	1.0646	0	0.0046363	1	134.57	132940000	12	48	77
A0A287AZY1	no	GLAPTAAPKAEPTA	14	30	Unmodified	_GLAPTAAPKAEPTA_	MSMS	2	647.853709	1293.69287	NaN	56.565	1	0	0.019368	1	54.982		7	53	86
A0A287BLD2	no	GPPSGGFDFSFLPQ	14	30	Unmodified	_GPPSGGFDFSFLPQ_	MULTI-MSMS	2	726.843342	1451.67213	0.024049	78.78	1.0683	0	0.003333	2	156.35	150950000	10	71	123
A0A5G2QMD8	no	GPQIPVPRPPVEYP	14	30	Unmodified	_GPQIPVPRPPVEYP_	MULTI-MSMS	2	773.424833	1544.83511	0.91451	54.88	1.3769	0	0.024723	1	123.79	27810000	3	82	176
A0A287AXU0	no	GVGVPGVGVPGVGV	14	30	Unmodified	_GVGVPGVGVPGVGV_	MULTI-MSMS	2	575.334956	1148.65536	1.9293	65.616	0.64056	0	0.012211	1	130.77	22688000	12	105	220
A0A287AXU0	no	GVPGVGVPGVGVPG	14	30	Unmodified	_GVPGVGVPGVGVPG_	MSMS	2	574.327131	1146.63971	NaN	55.344	1	0	0.026364	1	74.127		12	119	237
A0A287AXU0	no	GVSTGAVVPQLGAG	14	30	Unmodified	_GVSTGAVVPQLGAG_	MULTI-MSMS	2	606.832777	1211.651	-0.62307	45.882	0.74234	0	0.0018551	1	159.79	101940000	12	131	250
A0A287AXU0	no	GVYPGGVLPGTGAR	14	30	Unmodified	_GVYPGGVLPGTGAR_	MULTI-MSMS	2	650.854044	1299.69353	-1.9647	33.659	0.82421	0	0.0203	1	126.71	482010000	12	134	254
A0A287AXU0	no	IPGIGGIAGAGAPA	14	30	Unmodified	_IPGIGGIAGAGAPA_	MULTI-MSMS	1	1121.63133	1120.62406	0.44692	52.887	0.46839	-7.1054E-15	0.025797	1	100.19	16053000	12	140	268
A0A0H5ANC0	yes	LIEDIEDGTFSKLS	14	30	Unmodified	_LIEDIEDGTFSKLS_	MSMS	2	783.898511	1565.78247	NaN	54.265	1	0	0.020432	1	79.693		5	146	274
A0A287AXU0	no	LPGVGVGGVGVPGG	14	30	Unmodified	_LPGVGVGGVGVPGG_	MULTI-MSMS	1	1121.63133	1120.62406	2.2677	54.258	0.40639	0	0.025797	1	100.19	16846000	12	148	276
A0A287BLD2	no	LPQPPQEKAHDGGR	14	30	Deamidation (	_LPQPPQ(de)EKAHDGGR_	MULTI-MSMS	4	383.44694	1529.75865	-0.10768	8.6079	0.60145	0	0.023935	1	93.823	33319000	10	150	278
A0A286ZY60	no	LVTGNPGVPVPAAA	14	30	Unmodified	_LVTGNPGVPVPAAA_	MULTI-SECPEP	2	631.858795	1261.70304	-0.30075	61.414	0.57209	-7.1054E-15	0.025928	1	68.657	48777000	9	152	281
A0A287AXU0	no	PGGVPGGVFFPGAG	14	30	Unmodified	_PGGVPGGVFFPGAG_	MULTI-MSMS	2	608.31148	1214.60841	-0.88877	67.396	2.6738	0	0.012211	4	130.77	1925000000	12	157	289
A0A287AXU0	no	PGVGVLPGVPTGTG	14	30	Unmodified	_PGVGVLPGVPTGTG_	MULTI-MSMS	2	604.337695	1206.66084	-2.2251	55.973	0.847	0	4.8893E-07	2	186.3	147370000	12	159	291
F1SFA7	yes	SGGGYDFGYEGDFY	14	30	Unmodified	_SGGGYDFGYEGDFY_	MULTI-MSMS	2	767.293879	1532.5732	-0.12711	71.227	1.643	0	0.014968	1	129.38	60285000	15	175	315
A0A287BLD2	no	SYGYDEKSAGISVP	14	30	Unmodified	_SYGYDEKSAGISVP_	MULTI-MSMS	2	736.848821	1471.68309	-0.67092	32.89	1.5376	0	0.02301	1	94.363	17814000	10	180	326
F1SR80	no	VFVDLEPTVIDEVR	14	30	Unmodified	_VFVDLEPTVIDEVR_	MULTI-MSMS	2	815.93797	1629.86139	0.5743	68.606	0.6753	0	0.0055044	1	134.13	46110000	0	189	337
A0A287AXU0	no	VGGVPGAALSPAAQ	14	30	Unmodified	_VGGVPGAALSPAAQ_	MULTI-MSMS	2	597.827495	1193.64044	-0.47696	41.207	1.1059	0	0.013191	1	139.98	34564000	12	194	342
A0A287AXU0	no	VGPFGGQQPGVPLG	14	30	Unmodified	_VGPFGGQQPGVPLG_	MULTI-MSMS	2	655.348594	1308.68264	-0.11981	59.964	1.1646	0	0.025065	1	93.162	81219000	12	199	349
I3LQ84	yes	VGSDVDADVLSKIS	14	30	Unmodified	_VGSDVDADVLSKIS_	MULTI-MSMS	2	702.864471	1403.71439	-0.38271	47.706	0.42373	0	0.02601	1	92.611	12926000	18	202	352
A0A287AXU0	no	VGVAPGVGVAPGVG	14	30	Unmodified	_VGVAPGVGVAPGVG_	MULTI-MSMS	2	568.327131	1134.63971	-0.42717	45.806	0.71899	0	0.018035	1	127.84	106200000	12	204	354
A0A287AXU0	no	VGVPGVGVPGVGVP	14	30	Unmodified	_VGVPGVGVPGVGVP_	MULTI-MSMS	2	595.350606	1188.68666	0.35018	67.733	4.7655	0	0.024564	3	99.375	2928900000	12	209	359
A0A287AXU0	no	YPGGVLPGTGARFP	14	30	Unmodified	_YPGGVLPGTGARFP_	MSMS	2	694.869694	1387.72483	NaN	48.331	1	0	0.015705	1	112.71		12	233	393
A0A287AXU0	no	AGVLPGVGVGGVGVP	15	23	Unmodified	_AGVLPGVGVGGVGVP_	MULTI-MSMS	2	617.861338	1233.70812	0.10516	69.928	1.5751	0	0.016148	1	96.492	98149000	12	2	2
A0A287AXU0	no	APGVGVAPGIGIGPG	15	23	Unmodified	_APGVGVAPGIGIGPG_	MULTI-MSMS	2	609.845687	1217.67682	0.2625	55.448	0.74839	0	0.021026	1	130.34	62253000	12	5	5
A0A287AXU0	no	APGVGVAPGVGVAPG	15	23	Unmodified	_APGVGVAPGVGVAPG_	MULTI-MSMS	2	602.837862	1203.66117	-0.51163	42.788	0.39857	0	0.015668	1	96.253	5076100	12	6	6
A0A5G2QY90	no	APSTMKIKIIAPPER	15	23	Oxidation (M)	_APSTM(ox)KIKIIAPPER_	MULTI-MSMS	3	556.65528	1666.94401	0.56107	17.312	0.47924	0	0.018009	1	97.417	6547300	2	7	7
A0A287BLD2	no	FLPQPPQEKAHDGGR	15	23	Deamidation (	_FLPQ(de)PPQEKAHDGGR_	MULTI-SECPEP	3	559.949632	1676.82707	-0.66652	14.051	1.2988	0	0.027795	1	71.344	22770000	10	19	30
A0A287AXU0	no	GAGAIPGIGGIAGAG	15	23	Unmodified	_GAGAIPGIGGIAGAG_	MULTI-MSMS	2	569.814387	1137.61422	0.086296	55.704	1.602	7.1054E-15	0.017358	1	97.093	828250000	12	24	36
A0A287AXU0	no	GAIPGIGGIAGAGAP	15	23	Unmodified	_GAIPGIGGIAGAGAP_	MULTI-SECPEP	1	1178.6528	1177.64552	2.613	60.017	0.72377	0	0.027798	1	71.501	30615000	12	29	43
A0A287BLD2	no	GFSGLDGAKGDAGPA	15	23	Unmodified	_GFSGLDGAKGDAGPA_	MULTI-MSMS	2	660.314948	1318.61534	1.8801	23.702	0.38783	3.5527E-15	0.012986	1	136.5	18295000	10	33	47
A0A287AXU0	no	GGAGAIPGIGGIAGA	15	23	Unmodified	_GGAGAIPGIGGIAGA_	MULTI-MSMS	2	569.814387	1137.61422	0.23596	57.459	1.614	0	0.016148	1	96.492	2206300000	12	34	48
A0A287AXU0	no	GGVPGGVFFPGAGLG	15	23	Unmodified	_GGVPGGVFFPGAGLG_	MULTI-MSMS	2	644.837862	1287.66117	-0.23667	76.663	0.84404	0	0.027259	1	89.752	39035000	12	50	79
A0A287AXU0	no	GPFGGQQPGVPLGYP	15	23	Unmodified	_GPFGGQQPGVPLGYP_	MULTI-MSMS	2	735.872434	1469.73031	-1.3311	67.972	1.1964	0	0.011185	2	151.97	48722000	12	59	95
A0A287BLD2	no	GPPSGGFDFSFLPQP	15	23	Deamidation (	_GPPSGGFDFSFLPQ(de)P_	MULTI-MSMS	2	775.861731	1549.70891	-0.18422	80.557	0.64874	0	0.01682	1	95.573	8112500	10	72	125
A0A287AXU0	no	GVGDLGGAGIPGGVA	15	23	Unmodified	_GVGDLGGAGIPGGVA_	MULTI-SECPEP	2	598.817127	1195.6197	-0.021709	55.326	0.96709	0	0.027796	1	71.425	201870000	12	91	205
A0A287AXU0	no	GVGVPGVGVPGVGVP	15	23	Unmodified	_GVGVPGVGVPGVGVP_	MULTI-MSMS	2	623.861338	1245.70812	-0.85155	69.179	3.5756	0	0.0069585	4	166.17	2164900000	12	106	221
A0A287AXU0	no	GVLPGVGVGGVGVPG	15	23	Unmodified	_GVLPGVGVGGVGVPG_	MULTI-MSMS	2	610.853512	1219.69247	-0.13124	66.059	0.71584	0	0.020233	1	98.523	44284000	12	113	228
F1SQ09	yes	ILDHNLLENSKIKGR	15	23	Unmodified	_ILDHNLLENSKIKGR_	MULTI-MSMS	4	438.254706	1748.98972	-0.35959	16.82	0.73905	0	0.00716	1	155.28	6809300	16	139	266
A0A287BLD2	no	LSYGYDEKSAGISVP	15	23	Unmodified	_LSYGYDEKSAGISVP_	MULTI-MSMS	2	793.390853	1584.76715	-0.88926	39.351	2.0616	0	0.016195	1	132.25	73601000	10	151	280
A0A287AXU0	no	RPFPLGGVAPRPGFG	15	23	Unmodified	_RPFPLGGVAPRPGFG_	MULTI-SECPEP	3	508.952649	1523.83612	1.4256	40.144	0.90145	0	0.027751	1	68.83	41475000	12	172	310
A0A287BLD2	no	SGGFDFSFLPQPPQE	15	23	Unmodified	_SGGFDFSFLPQPPQE_	MULTI-MSMS	2	826.883195	1651.75184	-0.14588	79.71	0.82809	0	0.019677	1	147.91	73367000	10	173	311
F1SFA7	yes	SGGGYDFGYEGDFYR	15	23	Unmodified	_SGGGYDFGYEGDFYR_	MULTI-MSMS	2	845.344434	1688.67432	1.2102	51.953	0.8816	7.1054E-15	4.6968E-11	2	201.46	90906000	15	176	317
A0A287AXU0	no	VGGVPGAALSPAAQA	15	23	Unmodified	_VGGVPGAALSPAAQA_	MULTI-MSMS	2	633.346052	1264.67755	-1.3309	45.435	0.79544	0	0.020729	1	147.4	26016000	12	195	343
A0A287AXU0	no	VGPFGGQQPGVPLGY	15	23	Unmodified	_VGPFGGQQPGVPLGY_	MULTI-MSMS	2	736.880259	1471.74596	-0.54698	68.867	0.63061	0	0.023238	1	100.02	12052000	12	200	350
A0A287AXU0	no	YPGGVLPGTGARFPG	15	23	Unmodified	_YPGGVLPGTGARFPG_	MULTI-MSMS	2	723.380426	1444.7463	-0.95385	45.063	1.041	0	0.0045805	2	169.65	52834000	12	234	395
A0A287AXU0	no	GAGVLPGVGVGGVGVP	16	12	Unmodified	_GAGVLPGVGVGGVGVP_	MULTI-MSMS	2	646.372069	1290.72959	-0.22517	69.492	3.4161	0	0.018572	2	93.429	585670000	12	28	41
A0A287AXU0	no	GGAGAIPGIGGIAGAG	16	12	Unmodified	_GGAGAIPGIGGIAGAG_	MULTI-MSMS	2	598.325119	1194.63569	-0.30937	55.846	2.5119	0	0.019482	1	98.904	3245300000	12	35	49
F1SFA7	yes	GPSGGGYDFGYEGDFY	16	12	Unmodified	_GPSGGGYDFGYEGDFY_	MULTI-MSMS	2	844.330992	1686.64743	0.91334	71.232	1.0429	0	0.0037203	2	175.88	181730000	15	83	180
A0A287AXU0	no	GVGDLGGAGIPGGVAG	16	12	Unmodified	_GVGDLGGAGIPGGVAG_	MULTI-MSMS	2	627.327859	1252.64116	0.055234	53.748	0.94809	0	0.021734	1	100.02	142890000	12	92	206

A0A287AXU0	no	GVGVPGGAGAIPGIGG	16	12	Unmodified	_GVGVPGGAGAIPGIGG_	MULTI-MSMS	2	618.340769	1234.66699	1.6633	58.228	0.8427	0	0.014874	2	95.741	17286000	12	101	216
A0A287AXU0	no	GVGVPGVGVPGAVSPA	16	12	Unmodified	_GVGVPGVGVPGAVSPA_	MULTI-MSMS	2	660.369527	1318.7245	0.20874	62.033	0.51489	-7.1054E-15	0.025941	1	88.819	724630000	12	102	217
A0A287AXU0	no	GVPGVGVPGVGVPGAV	16	12	Unmodified	_GVPGVGVPGVGVPGAV_	MULTI-MSMS	2	659.379894	1316.74524	-0.078279	67.47	1.7647	0	0.021946	1	91.318	1332600000	12	120	238
A0A287AXU0	no	PGVGVPGVGVPGVGVP	16	12	Unmodified	_PGVGVPGVGVPGVGVP_	MULTI-MSMS	2	672.387719	1342.76089	0.2914	71.381	1.0376	0	0.0046202	1	174.17	54485000	12	160	292
A0A287BLD2	no	PSGGFDFSFLPQPPQE	16	12	Unmodified	_PSGGFDFSFLPQPPQE_	MULTI-MSMS	2	875.409577	1748.8046	-0.63511	79.515	0.90198	0	0.0097065	1	170.52	20524000	10	169	307
A0A287AXU0	no	VAPGVGVAPGIGIGPG	16	12	Unmodified	_VAPGVGVAPGIGIGPG_	MULTI-MSMS	2	659.379894	1316.74524	3.3649	59.467	0.48321	0	0.024263	1	132.08	16743000	12	184	332
A0A287AXU0	no	VGPFGGQQPGVPLGYP	16	12	Unmodified	_VGPFGGQQPGVPLGYP_	MULTI-MSMS	2	785.40664	1568.79873	-0.78329	72.102	2.142	0	0.014658	3	156.35	676240000	12	201	351
A0A287AXU0	no	VPGGVPGGVFFPGAGL	16	12	Unmodified	_VPGGVPGGVFFPGAGL_	MULTI-MSMS	2	714.387719	1426.76089	0.080882	79.554	0.5627	0	0.027736	1	87.696	44134000	12	224	375
I3LQ84	yes	DVDADVLSKISLGDEAA	17	12	Unmodified	_DVDADVLSKISLGDEAA_	MULTI-MSMS	2	859.428164	1716.84177	-0.66757	77.109	0.27935	0	0.017987	1	100.19	9742900	18	14	21
A0A287AXU0	no	GALGGVGDLGGAGIPGG	17	12	Unmodified	_GALGGVGDLGGAGIPGG_	MULTI-MSMS	2	662.846416	1323.67828	-0.41039	60.404	0.86141	0	0.018881	1	91.584	114430000	12	30	44
A0A287BLD2	no	GPPSGGFDFSFLPQPPQ	17	12	Unmodified	_GPPSGGFDFSFLPQPPQ_	MULTI-MSMS	2	887.925394	1773.83624	-1.161	78.791	0.85603	0	0.000024798	2	185.96	73647000	10	73	126
F1SFA7	yes	GPSGGGYDFGYEGDFYR	17	12	Unmodified	_GPSGGGYDFGYEGDFYR_	MULTI-MSMS	2	922.381548	1842.74854	-0.19333	53.66	1.0461	0	3.0504E-302	3	354.38	202810000	15	84	187
A0A287AXU0	no	GVGAGVPGFGVGAGVPG	17	12	Unmodified	_GVGAGVPGFGVGAGVPG_	MULTI-MSMS	2	677.859326	1353.7041	-1.1679	60.182	0.9818	0	0.017692	1	100.02	223300000	12	90	204
A0A287AXU0	no	GVPGVGVPGVGVPGVGV	17	12	Unmodified	_GVPGVGVPGVGVPGVGV_	MULTI-MSMS	2	701.906276	1401.798	0.27789	74.871	0.94926	0	0.021715	1	152.59	23251000	12	122	240
A0A287AXU0	no	GVPGVGVPGVGVPGVPG	17	12	Unmodified	_GVPGVGVPGVGVPGVPG_	MULTI-MSMS	2	700.898451	1399.78235	0.81764	71.121	1.3921	1.4211E-14	0.020519	1	101.64	168700000	12	125	243
A0A287AXU0	no	PGVGVPGVPGVGVPGVG	17	12	Unmodified	_PGVGVPGVPGVGVPGVG_	MULTI-MSMS	2	700.898451	1399.78235	2.3556	83.337	0.38032	0	0.016692	1	99.442	12368000	12	161	293
A0A287AXU0	no	PGVVGVPGVGVPGVGVP	17	12	Unmodified	_PGVVGVPGVGVPGVGVP_	MULTI-MSMS	2	721.921926	1441.8293	0.28262	75.294	1.6954	0	0.015599	3	157.86	892230000	12	162	294
A0A287BLD2	no	PPSGGFDFSFLPQPPQE	17	12	Deamidation (	_PPSGGFDFSFLPQ(de)PPQE_	MULTI-MSMS	2	924.427967	1846.84138	1.2857	80.088	0.60562	0	0.016088	1	93.258	9089700	10	166	304
A0A287AXU0	no	VGVPGVGVPGVGVPGVG	17	12	Unmodified	_VGVPGVGVPGVGVPGVG_	MULTI-MSMS	2	701.906276	1401.798	0.41426	73.72	1.3971	0	0.015599	1	93.551	2742600000	12	210	360
A0A287AXU0	no	VPGGVPGGVFFPGAGLG	17	12	Unmodified	_VPGGVPGGVFFPGAGLG_	MULTI-MSMS	2	742.898451	1483.78235	0.30775	78.539	1.0503	0	0.023279	1	88.948	171050000	12	225	376
A0A287AXU0	no	AGLGGVGGVGGLGVSTGA	18	12	Unmodified	_AGLGGVGGVGGLGVSTGA_	MULTI-MSMS	2	693.372798	1384.73104	0.062469	56.511	1.0258	7.1054E-15	0.0175	1	105.52	99842000	12	1	1
A0A287BLD2	no	DFSFLPQPPQEKAHDGGR	18	12	Unmodified	_DFSFLPQPPQEKAHDGGR_	MULTI-MSMS	3	675.997422	2024.97044	-0.43508	38.342	1.5928	0	0.014593	2	107.75	31507000	10	13	19
A0A287AXU0	no	GAGAIPGIGGIAGAGAPA	18	12	Unmodified	_GAGAIPGIGGIAGAGAPA_	MULTI-MSMS	2	689.377883	1376.74121	0.69334	59.776	1.2926	7.1054E-15	0.0095104	2	107.57	387130000	12	25	38
A0A287AXU0	no	GGAGAIPGIGGIAGAGAP	18	12	Unmodified	_GGAGAIPGIGGIAGAGAP_	MULTI-MSMS	2	682.370058	1362.72556	0.42945	61.197	2.187	0	0.012241	1	100.98	1602700000	12	36	50
A0A287BLD2	no	GPPSGGFDFSFLPQPPQE	18	12	Unmodified	_GPPSGGFDFSFLPQPPQE_	MULTI-MSMS	3	635.300219	1902.87883	-0.046347	79.564	1.0174	0	0.013663	2	106.38	83491000	10	74	132
F1SFA7	yes	GPSGGGYDFGYEGDFYRA	18	12	Unmodified	_GPSGGGYDFGYEGDFYRA_	MULTI-MSMS	2	957.900105	1913.78566	0.50299	55.329	1.0774	0	0.0094706	1	98.592	11105000	15	85	196
A0A287AXU0	no	GVGGVGGLGVSTGAVVPQ	18	12	Unmodified	_GVGGVGGLGVSTGAVVPQ_	MULTI-MSMS	2	755.91483	1509.81511	-0.55177	59.713	0.41606	0	0.015498	1	91.855	11616000	12	93	208
A0A287AXU0	no	GVGVGGVPGAALSPAAQA	18	12	Unmodified	_GVGVGGVPGAALSPAAQA_	MULTI-MSMS	2	739.901722	1477.78889	0.41772	56.503	0.81957	7.1054E-15	0.0098905	1	94.66	24308000	12	98	213
A0A287AXU0	no	GVPGVGVPGVGVPGVGVP	18	12	Unmodified	_GVPGVGVPGVGVPGVGVP_	MULTI-MSMS	2	750.432658	1498.85076	-0.07155	76.227	2.2991	0	4.6484E-10	3	196.55	914870000	12	123	241
A0A287AXU0	no	VGGVGVPGGAGAIPGIGG	18	12	Unmodified	_VGGVGVPGGAGAIPGIGG_	MULTI-MSMS	2	696.385708	1390.75686	1.3056	62.382	1.14	7.1054E-15	0.0094585	2	98.582	31499000	12	192	340
A0A287AXU0	no	VGVPGVGVPGVGVPGVPG	18	12	Unmodified	_VGVPGVGVPGVGVPGVPG_	MULTI-SECPEP	2	750.432658	1498.85076	0.64134	73.718	0.19134	0	6.109E-22	1	158.07	7620200	12	214	365
A0A287AXU0	no	VLPGVGVGGVGVPGGAGA	18	12	Unmodified	_VLPGVGVGGVGVPGGAGA_	MULTI-MSMS	2	710.401358	1418.78816	3.0813	59.524	0.50708	0	0.015507	1	103.8	20018000	12	220	371
A0A287BLD2	no	FDFSFLPQPPQEKAHDGGR	19	13	Unmodified	_FDFSFLPQPPQEKAHDGGR_	MULTI-MSMS	4	544.016989	2172.03885	-0.86394	49.789	2.0046	0	0.0048433	3	99.891	40581000	10	17	27
A0A287AXU0	no	GAGLGGVGGVGGLGVSTGA	19	13	Unmodified	_GAGLGGVGGVGGLGVSTGA_	MULTI-MSMS	2	721.88353	1441.75251	-0.11124	56.637	0.7121	0	0.0035683	1	95.822	183960000	12	27	40
A0A287AXU0	no	GGAGAIPGIGGIAGAGAPA	19	13	Unmodified	_GGAGAIPGIGGIAGAGAPA_	MULTI-MSMS	2	717.888615	1433.76268	0.047198	59.96	1.8871	-7.1054E-15	0.0060661	2	102.07	2482100000	12	37	52
A0A287AXU0	no	GGVGGVGGLGVSTGAVVPQ	19	13	Unmodified	_GGVGGVGGLGVSTGAVVPQ_	MULTI-MSMS	2	784.425562	1566.83657	1.0888	57.035	0.80782	-7.1054E-15	0.026421	1	153.12	381670000	12	43	71
A0A287BLD2	no	GPPSGGFDFSFLPQPPQEK	19	13	Deamidation (	_GPPSGGFDFSFLPQ(de)PPQEK_	MULTI-MSMS	2	1016.98618	2031.95781	1.7678	71.073	3.2758	-1.4211E-14	2.3722E-218	6	317.77	182120000	10	75	141
A0A287AXU0	no	GVAPGVGVAPGVGVAPGVG	19	13	Unmodified	_GVAPGVGVAPGVGVAPGVG_	MULTI-MSMS	2	758.92774	1515.84093	2.0396	58.296	0.45593	7.1054E-15	0.018862	1	108.3	16504000	12	88	202
A0A287AXU0	no	GVGGVGGLGVSTGAVVPQL	19	13	Unmodified	_GVGGVGGLGVSTGAVVPQL_	MULTI-MSMS	2	812.456862	1622.89917	-0.026716	75.511	0.31836	0	0.012732	1	91.855	13691000	12	94	209
A0A287AXU0	no	GVGGVGVPGGAGAIPGIGG	19	13	Unmodified	_GVGGVGVPGGAGAIPGIGG_	MULTI-MSMS	2	724.89644	1447.77833	-0.21591	64.267	0.84153	0	0.0079201	1	93.938	54671000	12	95	210
A0A287AXU0	no	GVGVGGIPTFGVGAGGFPG	19	13	Unmodified	_GVGVGGIPTFGVGAGGFPG_	MULTI-MSMS	2	801.917372	1601.82019	0.040613	78.938	0.36552	-1.4211E-14	0.0082006	1	105.4	38483000	12	97	212
A0A287AXU0	no	GVPGVGVPGVGVPGAVSPA	19	13	Unmodified	_GVPGVGVPGVGVPGAVSPA_	MULTI-MSMS	2	786.940847	1571.86714	-0.023887	70.677	1.0404	0	0.0236	1	87.149	663900000	12	121	239
A0A287AXU0	no	GVPGVGVPGVGVPGVGVPG	19	13	Unmodified	_GVPGVGVPGVGVPGVGVPG_	MULTI-MSMS	2	778.94339	1555.87223	-1.4634	73.864	0.24363	0	0.014924	1	90.906	2931200	12	124	242
A0A287AXU0	no	GVPGVGVPGVGVPGVPGVG	19	13	Unmodified	_GVPGVGVPGVGVPGVPGVG_	MULTI-MSMS	2	778.94339	1555.87223	1.0407	75.67	0.62349	0	0.004815	1	176.79	27037000	12	126	244
A0A5G2QKZ7	no	IVVELGTNPLKSSGIENGA	19	13	Deamidation (	_IVVELGTNPLKSSGIEN(de)GA_	MULTI-MSMS	2	950.007113	1897.99967	-0.16381	60.481	0.33591	0	0.0066613	1	102.87	10501000	4	142	270
A0A287AXU0	no	GGVGVGGIPTFGVGAGGFPG	20	8	Unmodified	_GGVGVGGIPTFGVGAGGFPG_	MULTI-MSMS	2	830.428104	1658.84166	0.48724	78.843	0.54146	0	0.010844	1	94.616	50550000	12	45	73
A0A287BLD2	no	GPPSGGFDFSFLPQPPQEKA	20	8	Unmodified	_GPPSGGFDFSFLPQPPQEKA_	MULTI-MSMS	2	1052.01273	2102.01091	0.63082	69.885	1.2803	0	0.0056491	2	173.06	16690000	10	76	144
A0A287AXU0	no	GVGVPGVGVPGVGVPGVPGV	20	8	Unmodified	_GVGVPGVGVPGVGVPGVPGV_	MULTI-MSMS	2	828.477597	1654.94064	0.090244	78.215	1.3021	0	0.022392	1	156.92	1069800000	12	107	222
A0A287AXU0	no	LGGVGGVGGLGVSTGAVVPQ	20	8	Unmodified	_LGGVGGVGGLGVSTGAVVPQ_	MULTI-MSMS	2	840.967593	1679.92063	0.14045	64.862	0.54452	0	9.4272E-11	1	195.27	44052000	12	145	273
A0A287AXU0	no	LPGVGVGGVGVPGGAGAIPG	20	8	Unmodified	_LPGVGVGGVGVPGGAGAIPG_	MULTI-MSMS	2	794.446297	1586.87804	-1.8742	68.135	1.1232	0	0.0082524	1	103.7	48935000	12	149	277
I3LQ84	yes	VGSDVDADVLSKISLGDEAA	20	8	Unmodified	_VGSDVDADVLSKISLGDEAA_	MULTI-MSMS	2	980.989117	1959.96368	2.6475	60.636	0.077019	0	0.013971	1	91.858	8988400	18	203	353
A0A287AXU0	no	VGVGGVGVPGGAGAIPGIGG	20	8	Unmodified	_VGVGGVGVPGGAGAIPGIGG_	MULTI-MSMS	2	774.430647	1546.84674	1.653	69.855	1.7333	0	0.015288	1	90.696	110970000	12	205	355
A0A287AXU0	no	VGVPGVGVPGVGVPGVGVPG	20	8	Unmodified	_VGVPGVGVPGVGVPGVGVPG_	MULTI-MSMS	2	828.477597	1654.94064	0.017985	77.131	0.99183	0	0.0088045	1	98.249	3284500000	12	211	361
A0A287AXU0	no	GGAGAIPGIGGIAGAGAPAAA	21	3	Unmodified	_GGAGAIPGIGGIAGAGAPAAA_	MULTI-MSMS	2	788.925729	1575.8369	0.41778	63.557	0.85999	0	0.015117	1	94.409	82317000	12	38	54
A0A287AXU0	no	GPGVVGVPGVGVPGVGVPGVG	21	3	Unmodified	_GPGVVGVPGVGVPGVGVPGVG_	MULTI-MSMS	2	856.988329	1711.9621	-0.49455	77.714	1.1458	1.4211E-14	4.0218E-11	1	195.7	327010000	12	62	99
A0A287BLD2	no	GPPSGGFDFSFLPQPPQEKAH	21	3	Unmodified	_GPPSGGFDFSFLPQPPQEKAH_	MULTI-MSMS	3	747.363882	2239.06982	-0.84022	60.243	3.5586	0	0.013892	3	115.57	463870000	10	77	147
A0A287AXU0	no	AKAGAGLGGVGGVGGLGVSTGA	22	7	Unmodified	_AKAGAGLGGVGGVGGLGVSTGA_	MULTI-MSMS	2	856.968125	1711.9217	1.0234	41.884	0.42841	0	0.017491	1	97.69	21365000	12	3	3
A0A287BLD2	no	GPPSGGFDFSFLPQPPQEKAHD	22	7	Deamidation (	_GPPSGGFDFSFLPQ(de)PPQEKAHD_	MULTI-MSMS	3	786.034202	2355.08078	0.9541	62.662	1.8893	0	0.0082541	3	104.84	38835000	10	78	151
A0A287AXU0	no	GVPGVGVPGVGVPGVPGVGVPG	22	7	Unmodified	_GVPGVGVPGVGVPGVPGVGVPG_	MULTI-MSMS	2	905.514711	1809.01487	0.23466	77.884	0.2858	-1.4211E-14	3.8901E-09	1	192.32	25461000	12	127	245
A0A287AXU0	no	PGGVPGGVFFPGAGLGGLGGGA	22	7	Unmodified	_PGGVPGGVFFPGAGLGGLGGGA_	MULTI-MSMS	2	899.467761	1796.92097	-0.87835	80.051	0.63028	0	0.013771	1	89.827	35614000	12	158	290
A0A287AXU0	no	PGVVGVPGVGVPGVGVPGVGVP	22	7	Unmodified	_PGVVGVPGVGVPGVGVPGVGVP_	MULTI-MSMS	2	926.538186	1851.06182	0.13927	79.47	0.70252	0	0.021095	1	147.12	147400000	12	163	295
A0A287BLD2	no	SGGFDFSFLPQPPQEKAHDGGR	22	7	Unmodified	_SGGFDFSFLPQPPQEKAHDGGR_	MULTI-MSMS	4	594.285728	2373.11381	0.33594	51.81	1.6208	0	0.014559	2	97.69	46378000	10	174	314
A0A287AXU0	no	VAPGVGVAPGVGVAPGIGIGPG	22	7	Unmodified	_VAPGVGVAPGVGVAPGIGIGPG_	MULTI-MSMS	2	899.514711	1797.01487	0.49492	73.389	0.495	0	0.012721	1	101.38	32961000	12	185	333
A0A287AXU0	no	GPGIGVAPGVGVAPGVGVAPGVG	23	4	Unmodified	_GPGIGVAPGVGVAPGVGVAPGVG_	MULTI-MSMS	2	921.017618	1840.02068	2.7629	66.73	0.37883	0	0.021374	1	118.4	11756000	12	61	97
A0A287AXU0	no	GPGVVGVPGVGVPGVGVPGVGVP	23	4	Unmodified	_GPGVVGVPGVGVPGVGVPGVGVP_	MULTI-MSMS	2	955.048918	1908.08328	-0.11913	79.758	0.57554	0	0.023961	1	143.89	111080000	12	63	100
A0A287BLD2	no	GPPSGGFDFSFLPQPPQEKAHDG	23	4	Unmodified	_GPPSGGFDFSFLPQPPQEKAHDG_	MULTI-MSMS	3	804.713351	2411.11822	-1.0569	62.698	4.1847	0	0.0078038	3	145.44	354690000	10	79	154
A0A287BLD2	no	PSGGFDFSFLPQPPQEKAHDGGR	23	4	Unmodified	_PSGGFDFSFLPQPPQEKAHDGGR_	MULTI-MSMS	4	618.548919	2470.16657	0.066528	52.035	2.1562	7.1054E-15	0.010681	1	90.308	51543000	10	170	308
A0A287AXU0	no	GPGVVGVPGVGVPGVGVPGVGVPG	24	6	Unmodified	_GPGVVGVPGVGVPGVGVPGVGVPG_	MULTI-MSMS	2	983.55965	1965.10475	1.1346	79.119	0.57568	0	5.7551E-08	1	183.64	216300000	12	64	101
A0A287BLD2	no	GPPSGGFDFSFLPQPPQEKAHDGG	24	6	Unmodified	_GPPSGGFDFSFLPQPPQEKAHDGG_	MULTI-MSMS	3	823.720506	2468.13969	0.062038	59.159	1.5091	0	0.0055007	1	87.319	19801000	10	80	158
A0A287BLD2	no	PPSGGFDFSFLPQPPQEKAHDGGR	24	6	Unmodified	_PPSGGFDFSFLPQPPQEKAHDGGR_	MULTI-MSMS	4	642.81211	2567.21934	-0.39411	53.124	2.1106	7.1054E-15	0.0064236	1	88.836	28472000	10	167	305
A0A287AXU0	no	VAPGVGVAPGVGVAPGVGVAPGIG	24	6	Unmodified	_VAPGVGVAPGVGVAPGVGVAPGIG_	MULTI-MSMS	2	977.55965	1953.10475	1.3124	73.938	0.27271	0	0.0061834	1	88.441	4045200	12	186	334
A0A287AXU0	no	VAPGVGVAPGVGVAPGVGVAPGVG	24	6	Unmodified	_VAPGVGVAPGVGVAPGVGVAPGVG_	MULTI-MSMS	2	970.551825	1939.0891	-0.46431	69.246	0.59777	0	0.0077586	2	115.57	78512000	12	187	335
A0A287AXU0	no	VGVPGVGVPGVGVPGVGVPGVPGV	24	6	Unmodified	_VGVPGVGVPGVGVPGVGVPGVPGV_	MULTI-MSMS	2	1004.58312	2007.1517	0.52311	80.712	0.71503	0	0.0068165	1	113.47	136380000	12	212	362
A0A287BLD2	no	GPPSGGFDFSFLPQPPQEKAHDGGR	25	4	Unmodified	_GPPSGGFDFSFLPQPPQEKAHDGGR_	MULTI-MSMS	3	875.754209	2624.2408	-1.9307	50.673	3.685	-7.1054E-15	6.2E-21	8	209.41	168120000	10	81	162
A0A287AXU0	no	GVGVPGVGVPGVGVPGVPGVGVPGV	25	4	Unmodified	_GVGVPGVGVPGVGVPGVPGVGVPGV_	MULTI-MSMS	2	1033.09386	2064.17316	1.7798	80.801	0.80038	-1.4211E-14	0.0028248	1	112.13	74670000	12	108	223
A0A287AXU0	no	GVGVPGVPGVGVPGVGVPGAVSPAA	25	4	Unmodified	_GVGVPGVPGVGVPGVGVPGAVSPAA_	MULTI-MSMS	2	1027.07566	2052.13677	2.3615	78.477	0.32723	0	0.017402	1	65.563	41213000	12	109	224
A0A287AXU0	no	VGVPGVGVPGVGVPGVGVPGVPGVG	25	4	Unmodified	_VGVPGVGVPGVGVPGVGVPGVPGVG_	MULTI-MSMS	2	1033.09386	2064.17316	1.1593	80.128	0.45687	-1.4211E-14	0.0028248	1	112.13	367470000	12	213	363

Bovine pericardium

Accession	Unique pr	Sequence	Length	Count	Modificati	Modified s	Missed cle	Type	Charge	m/z	Mass	ppm	Retention time	Retention l	Retention time calibration	PEP	MS/MS count	Score	Intensity	Protein group ID	Peptide ID	Evidence ID
A0A3Q1N	no	FDGDFYRA	8	11	Unmodified	_FDGDFYRA_		MULTI-MS	2	495.719424	989.424296	0.68751	32.239	0.93579	0	0.028313	2	148.98	211240000	5	32	56
A0A3Q1N	no	FGFDGDFY	8	11	Unmodified	_FGFDGDFY_		MULTI-SEC	1	967.383225	966.375948	-2.9755	77.35	0.26577	0	0.0029111	1	95.793	2894200	5	36	76
P02453	yes	FLPQPPQE	8	11	Deamidation	_FLPQ(de)PPQE_		MULTI-MS	2	478.739825	955.465098	0.47128	38.449	1.3666	0	0.02753	1	120.65	16443000	4	42	85
P02453	yes	FSGLDGAK	8	11	Unmodified	_FSGLDGAK_		MULTI-MS	2	397.705786	793.397018	-0.26392	12.036	1.0625	0	0.027601	1	120.62	67803000	4	50	123
A0A3Q1N	no	GFDGDFYR	8	11	Unmodified	_GFDGDFYR_		MULTI-MS	2	488.711599	975.408646	-0.50944	42.455	1.3481	0	0.000025107	3	168.38	311480000	5	69	178
A4IFU5	no	GLQFPVGR	8	11	Unmodified	_GLQFPVGR_		MULTI-SEC	2	437.250695	872.486836	0.37643	31.046	0.77938	0	0.0099224	1	89.673	85063000	18	97	310
P02453	yes	GPPSGGYD	8	11	Unmodified	_GPPSGGYD_		MULTI-SEC	1	749.31006	748.302784	-0.28427	10.103	1.0438	0	0.03467	1	77.374	4314400	4	114	350
P13605	yes	IPSPLPRS	8	11	Unmodified	_IPSPLPRS_		MULTI-MS	2	433.758352	865.502152	0.67531	19.644	1.5516	0	0.011602	1	153.73	45880000	7	207	677
P21793	yes	IVVELGTN	8	11	Unmodified	_IVVELGTN_		MULTI-SEC	1	844.47746	843.470183	-0.4285	38.716	0.34154	0	0.0042064	1	94.662	2257900	9	219	693
A0A3Q1LT	no	KYNKLFNR	8	11	2 Deamida	_KYN(de)KLFN(de)R_		MULTI-MA	3	362.197708	1083.57129	-0.28595	8.007	0.16466	0	NaN	0	NaN	884440	19	223	698
F1N0H9;A	no	VGGIPTFG	8	11	Unmodified	_VGGIPTFG_		MULTI-SEC	1	747.403566	746.39629	-0.48157	47.321	0.69317	0	0.025949	1	81.1	40512000	21;20	339	972
F1MKG2	yes	ALLQFGGPR	9	13	Unmodified	_ALLQFGGPR_		MULTI-MS	2	479.777077	957.5396	0.58569	34.744	0.50318	0	0.015482	1	128.38	12608000	29	9	16
F1N0H9;A	no	APGIGLGPG	9	13	Unmodified	_APGIGLGPG_		MULTI-MA	1	738.414465	737.407189	0.93435	26.76	0.32908	3.5527E-15	NaN	0	NaN	2163300	21;20;6	14	22
A0A3Q1N	no	FGFDGDFYR	9	13	Unmodified	_FGFDGDFYR_		MULTI-MS	2	562.245806	1122.47706	-1.831	51.444	0.85699	0	0.010635	1	136.16	48149000	5	37	77
A0A3Q1N	no	GFDGDFYRA	9	13	Unmodified	_GFDGDFYRA_		MULTI-MS	2	524.230156	1046.44576	0.26437	38.608	0.74632	0	0.010798	2	138.24	86297000	5	70	191
P02453;G3	no	GPAGEKGAP	9	13	Unmodified	_GPAGEKGAP_		MULTI-MA	2	392.20341	782.392267	-1.9484	7.0486	0.38883	0	NaN	0	NaN	5941800	4;31	101	321
P02453	yes	GRTGDAGPA	9	13	Unmodified	_GRTGDAGPA_		MSMS	2	401.196116	800.37768	NaN	0.0079918	1	0	0.01942	1	80.462		4	145	583
G3MZI7	yes	GTMLMLPFR	9	13	2 Oxidation	_GTM(ox)LM(ox)LPFI		MULTI-MS	2	549.277738	1096.54092	-0.5696	44	1.1215	0	0.014833	1	129.4	58504000	31	147	586
P13605	yes	ILLDLSYNH	9	13	Deamidation	_ILLDLSYN(de)H_		MULTI-MS	2	544.784764	1087.55498	1.3216	53.892	0.64588	0	0.023257	1	124.08	11397000	7	201	669
F1MKG2	no	LDQVALSWR	9	13	Unmodified	_LDQVALSWR_		MULTI-MS	2	544.298373	1086.58219	0.87316	36.951	0.53854	0	8.3879E-06	1	164.57	8182300	29	227	704
A6QQQ3	no	LPRNLEQLR	9	13	Unmodified	_LPRNLEQLR_		MULTI-MS	3	380.22789	1137.66184	0.085494	21.049	0.7546	0	0.010775	1	137.95	43647000	17	246	728
F1MKG2	no	SLQSISSFR	9	13	Unmodified	_SLQSISSFR_		MULTI-MS	2	512.774731	1023.53491	0.56788	25.934	0.43271	0	0.010644	1	136.27	6709600	29	304	913
A7E303	yes	VHSSFSGYL	9	13	Unmodified	_VHSSFSGYL_		MULTI-MS	2	498.742899	995.471246	0.61314	30.286	0.80381	0	0.017304	1	126.24	26591000	24	351	991
E1BI98	yes	VPSYQALLR	9	13	Unmodified	_VPSYQALLR_		MULTI-MA	2	523.803291	1045.59203	-0.36666	31.811	0.56101	0	NaN	0	NaN	6432200	28	369	1019
A0A452DJ	no	AGFAGDDAPR	10	10	Unmodified	_AGFAGDDAPR_		MSMS	2	488.727781	975.441008	NaN	11.037	1	0	0.014553	1	75.378		11	5	7
A6QQQ3	no	ALPRNLEQLR	10	10	Deamidation	_ALPRN(de)LEQLR_		MULTI-MS	3	404.234933	1209.68297	-0.27978	28.84	0.97459	0	0.024204	1	120.99	10807000	17	11	18
A0A3Q1N	no	EFGFDGDFYR	10	10	Unmodified	_EFGFDGDFYR_		MULTI-MS	2	626.767103	1251.51965	0.51315	59.313	0.8933	-7.1054E-15	2.8181E-11	1	180.45	24655000	5	27	40
P02453	yes	GPPSGGYDLS	10	10	Unmodified	_GPPSGGYDLS_		MULTI-MS	2	475.216714	948.418876	-0.3343	23.832	2.9752	0	0.0015277	4	147.69	43053000	4	115	352
A0A3Q1N	no	GPSGGGYEFG	10	10	Unmodified	_GPSGGGYEFG_		MULTI-MS	1	927.384288	926.377011	0.48945	23.002	0.68058	0	6.9031E-60	1	224.8	13088000	5	133	471
E1BI98	yes	HYSDEVEIIR	10	10	Unmodified	_HYSDEVEIIR_		MULTI-MS	2	630.814584	1259.61462	1.6565	22.466	0.50605	0	0.0053762	1	133.13	13249000	28	184	650
F1N401	yes	IVEVFEIGPK	10	10	Unmodified	_IVEVFEIGPK_		MULTI-SEC	2	565.826432	1129.63831	0.63033	47.493	0.37054	-7.1054E-15	0.0024034	1	101.64	5151500	30	216	686
P21793	yes	KMPKTLQELR	10	10	Oxidation (	_KM(ox)PKTLQELR_		MSMS	3	420.57619	1258.70674	NaN	7.8187	1	0	0.000048164	1	104.79		9	220	694
F1MKG2	yes	LGEQNFHKVR	10	10	Unmodified	_LGEQNFHKVR_		MULTI-SEC	3	409.891278	1226.652	1.648	7.7201	0.22266	0	0.015917	1	93.649	3183900	29	229	706
F1MKG2	no	VINVVNRLGA	10	10	Unmodified	_VINVVNRLGA_		MSMS	2	527.822015	1053.62948	NaN	36.442	1	0	0.012851	1	75.854		29	355	995
E1BB91	yes	DYLDVGFDTTR	11	12	Unmodified	_DYLDVGFDTTR_		MULTI-MS	2	651.304049	1300.59355	1.43	53.076	0.86081	0	0.00036456	1	142.43	13422000	26	26	35
A0A3Q1N	no	EFGFDGDFYRA	11	12	Unmodified	_EFGFDGDFYRA_		MSMS	2	662.28566	1322.55677	NaN	65.186	1	0	0.024135	1	68.735		5	28	44
F1N0H9;A	no	FPGALVPGGPA	11	12	Unmodified	_FPGALVPGGPA_		MSMS	2	491.77146	981.528367	NaN	53.897	1	0	0.030767	1	65.672		21;20	48	121
P02453	yes	FSGLDGAKGDA	11	12	Unmodified	_FSGLDGAKGDA_		MULTI-MS	2	519.248546	1036.48254	-1.3015	15.336	0.50843	0	0.032982	1	112.11	142210000	4	51	125
G3MZI7	no	GPPGEVIQPLP	11	12	Unmodified	_GPPGEVIQPLP_		MULTI-MS	2	552.308406	1102.60226	0.46267	49.942	1.0646	0	0.0054104	1	130.27	11943000	31	110	340
P02453	yes	GPPSGGYDLSF	11	12	Unmodified	_GPPSGGYDLSF_		MULTI-MS	2	548.750921	1095.48729	-0.16415	54.597	2.0144	0	0.000015333	4	151.29	37664000	4	116	354
A0A3Q1N	no	GPSGGGYEFGF	11	12	Unmodified	_GPSGGGYEFGF_		MULTI-MS	2	537.729989	1073.44543	-0.53717	57.416	1.2899	-7.1054E-15	0.0027055	1	133.81	32071000	5	134	473
A6QQQ3	no	IPPGVFSKLEN	11	12	Unmodified	_IPPGVFSKLEN_		MULTI-MS	2	600.834788	1199.65502	-0.0011987	37.505	1.4407	0	0.024613	1	114.39	30618000	17	206	675
F1N401	yes	IVEVFEIGPKR	11	12	Unmodified	_IVEVFEIGPKR_		MSMS	2	643.876988	1285.73942	NaN	36.05	1	0	2.0109E-08	1	114.84		30	217	688
E1BGN3	no	TIMPKDIQLAR	11	12	Oxidation (	_TIM(ox)PKDIQLAR_		MULTI-MS	3	434.579712	1300.71731	0.14467	20.38	1.3023	0	0.000015898	2	151.15	25067000	13	316	931
F1N0H9;A	no	VLGAGQPFPIG	11	12	Unmodified	_VLGAGQPFPIG_		MSMS	2	528.297842	1054.58113	NaN	61.111	1	0	0.031939	1	64.757		21;20;6	360	1002
E1BB91	yes	VVESLDVGPDR	11	12	Unmodified	_VVESLDVGPDR_		MULTI-MS	2	593.309135	1184.60372	-0.13733	25.024	0.70851	0	8.4774E-06	1	153.05	19310000	26	378	1043
F1MKG2	yes	ALLQFGGPREQQ	12	19	Unmodified	_ALLQFGGPREQQ_		MULTI-MS	2	672.356951	1342.69935	-1.6136	34.572	0.43744	0	0.0015237	1	125.97	13809000	29	10	17
P02453	yes	GFSGLDGAKGDA	12	19	Unmodified	_GFSGLDGAKGDA_		MULTI-MS	2	547.759278	1093.504	-0.53276	18.371	1.3537	0	0.0015455	2	125.71	594200000	4	73	207
A0A3Q1N	no	GGGYEFGFDGDF	12	19	Unmodified	_GGGYEFGFDGDF_		MSMS	2	634.248743	1266.48293	NaN	76.254	1	0	0.010416	1	95.909		5	76	229
F1N0H9;A	no	GGQQPGVPLGYP	12	19	Unmodified	_GGQQPGVPLGYP_		MULTI-MS	2	585.301113	1168.58767	-0.45487	44.831	1.126	-7.1054E-15	0.033177	1	95.909	22100000	21;20	81	284
A0A3Q1N	no	GLQGVQGGKGEQ	12	19	Unmodified	_GLQGVQGGKGEQ_		MULTI-MS	2	579.299101	1156.58365	-0.96632	7.9187	0.24773	0	0.0046997	1	112.75	43618000	5	98	312
P02453	yes	GPAGERGEQGPA	12	19	Unmodified	_GPAGERGEQGPA_		MSMS	2	563.267801	1124.52105	NaN	7.464	1	0	3.1067E-67	1	176.6		4	103	324
P02453	yes	GPPSGGYDLSFL	12	19	Unmodified	_GPPSGGYDLSFL_		MULTI-MS	2	605.292953	1208.57135	0.15098	72.435	2.2289	0	5.7222E-13	3	170.66	28745000	4	117	359
A0A3Q1N	no	GPSGGGYEFGFD	12	19	Unmodified	_GPSGGGYEFGFD_		MULTI-MS	2	595.24346	1188.47237	-1.1477	55.855	1.7611	0	0.0021554	1	129.42	32160000	5	135	477
F1N0H9;A	no	GVLGAGQPFPIG	12	19	Unmodified	_GVLGAGQPFPIG_		MULTI-MS	2	556.808574	1111.60259	0.51098	66.926	1.2194	-1.4211E-14	0.014235	1	104.45	49476000	21;20;6	165	614
A0A3Q1N	no	GYEFGFDGDFYR	12	19	Unmodified	_GYEFGFDGDFYR_		MULTI-MS	2	736.809499	1471.60444	-0.17632	64.332	0.7754	0	0.0019817	2	140.45	15793000	5	180	643
P13605	yes	KVPDGLPSALEQ	12	19	Unmodified	_KVPDGLPSALEQ_		MULTI-MS	2	627.340435	1252.66632	-0.000064733	34.663	1.3718	0	0.0030023	1	115.7	30970000	7	222	696
F1N0H9;A	no	STGAVVPQLGAG	12	19	Unmodified	_STGAVVPQLGAG_		MULTI-MS	2	528.787838	1055.56112	0.15568	38.359	1.2086	0	0.0019576	2	120.87	93463000	21;20;6	307	918
E1BB91	yes	VDYLDVGFDTTR	12	19	Unmodified	_VDYLDVGFDTTR_		MULTI-MS	2	700.838256	1399.66196	-0.30953	55.644	1.2509	0	0.000038565	2	151.15	73845000	26	328	956
A7E303	yes	VFLQMPSEQAAG	12	19	Oxidation (	_VFLQM(ox)PSEQAA		MULTI-SEC	2	647.31082	1292.60709	3.6104	35.444	0.47252	-7.1054E-15	4.4947E-06	1	108.81	19128000	24	331	962
F1N0H9;A	no	VGPFGGQQPGVP	12	19	Unmodified	_VGPFGGQQPGVP_		MULTI-MS	2	570.29583	1138.57711	0.5578	47.274	1.2814	0	0.0017969	2	130.04	517620000	21;20	341	975
F1N0H9;A	no	VLGAGQPFPIGG	12	19	Unmodified	_VLGAGQPFPIGG_		MULTI-MS	2	556.808574	1111.60259	1.2874	59.445	1.2618	0	0.0056848	1	111.04	54522000	21;20;6	361	1003
F1N0H9;A	no	VPGAVGLGGVSP	12	19	Unmodified	_VPGAVGLGGVSP_		MULTI-MS	2	505.287474	1008.5604	-0.49422	45.043	0.88386	0	0.020252	1	101.69	14571000	21;20;6	365	1012
E1BGN3	no	VTIMPKDIQLAR	12	19	Oxidation (	_VTIM(ox)PKDIQLAR		MULTI-MS	3	467.602517	1399.78572	0.28036	27.821	1.3378	0	0.027967	1	98.156	59250000	13	375	1038
F1N0H9;A	no	YPGGVLPGAGAR	12	19	Unmodified	_YPGGVLPGAGAR_		MULTI-MS	2	557.803823	1113.59309	-0.86416	24.443	1.2081	0	0.020186	2	101.72	1058100000	21;20;6	388	1059
A0A3Q1M	no	ADSDVSTAIDLFR	13	24	Unmodified	_ADSDVSTAIDLFR_		MULTI-MS	2	705.348988	1408.68342	2.2389	56.847	0.57918	0	0.0087879	1	125.22	7619100	10	4	5
F1N0H9;A	no	APGAIPGIGGIAG	13	24	Unmodified	_APGAIPGIGGIAG_		MULTI-MS	2	525.800749	1049.58694	0.97563	56.744	0.94969	0	0.030947	1	91.041	65623000	21;20;6	13	20
F1N0H9;A	no	GAVGLGGVSPAAA	13	24	Unmodified	_GAVGLGGVSPAAA		MULTI-SEC	2	513.782556	1025.55056	0.58081	37.308	0.4723	0	0.026411	1	85.212	15052000	21;20;6	64	149
A0A3Q1N	no	GGGYEFGFDGDFY	13	24	Unmodified	_GGGYEFGFDGDFY		MULTI-MS	2	715.780407	1429.54626	-0.3203	78.356	1.1671	0	0.0087708	2	125.31	181720000	5	77	235
A0A3Q1N	no	GGYEFGFDGDFYR	13	24	Unmodified	_GGYEFGFDGDFYR		MULTI-MS	2	765.320231	1528.62591	1.1219	64.798	1.13	0	0.027623	1	93.058	9781200	5	89	298
P02453	yes	GPPSGGYDLSFLP	13	24	Unmodified	_GPPSGGYDLSFLP_		MULTI-MS	2	653.819335	1305.62412	0.50064	76.328	0.95188	0	0.016219	1	101.54	10048000	4	118	368
A0A3Q1N	no	GPSGGGYEFGFDG	13	24	Unmodified	_GPSGGGYEFGFDG		MULTI-MS	2	623.754192	1245.49383	0.45488	55.499	0.54803	0	8.5799E-08	1	156.62	12757000	5	136	482
F1N0H9;A	no	GVAPGVGVVPGVG	13	24	Unmodified	_GVAPGVGVVPGVG		MULTI-MS	2	532.808574	1063.60259	-0.73058	51.605	0.57986	7.1054E-15	0.010536	1	110.63	38661000	21;20;6	148	587
F1N0H9;A	no	GVLGAGQPFPIGG	13	24	Unmodified	_GVLGAGQPFPIGG		MULTI-MS	2	585.319306	1168.62406	-0.21473	65.385	1.5235	0	0.0090561	2	123.69	269380000	21;20;6	166	615
F1N0H9;A	no	GVPGVGVPGVGVP	13	24	Unmodified	_GVPGVGVPGVGVP		MSMS	2	545.816399	1089.61824	NaN	60.633	1	0	0.000080485	1	105.12		21;20;6	171	622
F1N0H9;A	no	GVVPGVGVVPGVG	13	24	Unmodified	_GVVPGVGVVPGVG		MULTI-MS	2	546.824224	1091.63389	-0.13314	63.662	1.7547	-7.1054E-15	0.008936	2	124.37	131100000	21;20;6	176	635
F1N0H9;A	no	IGGVGGLGVSTGA	13	24	Unmodified	_IGGVGGLGVSTGA_		MULTI-SEC	1	1044.5684	1043.56112	-0.50655	41.753	0.55088	0	0.028376	1	82.452	4380200	21;20;6	196	664
E1BB91	yes	ITPLFLTSQEDRQ	13	24	Unmodified	_ITPLFLTSQEDRQ_		MULTI-MS	2	774.406837	1546.79912	0.25372	47.304	0.74032	0	0.031158	1	90.913	8761500	26	213	683
E1BB91	yes	LVDYLDVGFDTTR	13	24	Unmodified	_LVDYLDVGFDTTR_		MULTI-MS	2	757.380288	1512.74602	0.81359	62.83	0.48864	0	0.025581	1	94.297	3464500	26	254	752
F1N0H9;A	no	LVPGAPGAIPGVP	13	24	Unmodified	_LVPGAPGAIPGVP_		MULTI-SEC	2	572.839874	1143.66519	0.014102	62.713	0.88417	7.1054E-15	0.029167	1	73.616	64377000	21;20	258	758
G5E6E9	yes	NKALCIQMAAVSW	13	24	Oxidation (	_N(de)KALCIQM(ox)A		MULTI-MS	2	726.354705	1450.69486	-0.84589	24.919	1.3248	3.5527E-15	0.032852	1	89.886	1600300000	32	264	770
E1BB91	yes	PLGSRIEEGVPQF	13	24	Unmodified	_PLGSRIEEGVPQF_		MULTI-MS	2	714.877716	1427.74088	-2.4097	47.825	0.72245	0	0.010434	1	112.83	33217000	26	272	780
A0A3Q1N	no	SGGGYEFGFDGDF	13	24	Unmodified	_SGGGYEFGFDGDF		MULTI-MS	2	677.764757	1353.51496	-0.17387	75.722	1.4558	0	0.00007032	1	152.34	14642000	5	297	863
E1BB91	yes	TYKSQASVLDAIR	13	24	Unmodified	_TYKSQASVLDAIR_		MULTI-MA	3	484.599941	1450.77799	-0.21216	22.482	0.65043	0	NaN	0	NaN	4119300	26	318	935
F1N0H9;A	no	VGPFGGQQPGVPL	13	24	Unmodified	_VGPFGGQQPGVPL		MULTI-MS	2	626.837862	1251.66117	-0.23752	63.6	0.91189	-7.1054E-15	0.01955	2	98.009	83118000	21;20	342	978
E1BB91	yes	VIDELDVKPEGTR	13	24	Unmodified	_VIDELDVKPEGTR_		MULTI-MS	2	735.893563	1469.77257	1.0924	23.031	0.37236	0	0.027294	1	93.258	7295300	26	352	992
F1N0H9;A	no	VPGAVGLGGVSPA	13	24	Unmodified	_VPGAVGLGGVSPA_		MULTI-MS	2	540.806031	1079.59751	0.0037998	44.166	0.98485	0	0.029531	1	91.9	63340000	21;20;6	366	1015
E1BB91	yes	VVTYNNEVTTEIR	13	24	Unmodified	_VVTYNNEVTTEIR_		MULTI-MS	2	769.39647	1536.77839	-0.47954	28.943	0.43805	0	0.022587	1	96.113	8439700	26	382	1049
F1N0H9;A	no	VYPGGVLPGAGAR	13	24	Unmodified	_VYPGGVLPGAGAR		MULTI-MS	2	607.33803	1212.66151	-1.1266	29.488	1.9548	0	0.010075	3	117.89	2870600000	21;20;6	384	1053
F1N0H9;A	no	AAAAKAAAKAAQFG	14	21	Unmodified	_AAAAKAAAKAAQFG		MULTI-MS	2	623.848761	1245.68297	-1.7893	9.9231	0.30756	0	0.025093	1	89.029	10054000	21;20;6	1	1
A0A3Q1M	no	AADSDVSTAIDLFR	14	21	Unmodified	_AADSDVSTAIDLFR		MSMS	2	740.867545	1479.72054	NaN	60.379	1	0	4.3571E-06	1	110.08		10	3	3
A7E303	yes	APFPPVGAPVKFDK	14	21	Unmodified	_APFPPVGAPVKFDK		MULTI-MS	3	490.609889	1468.80784	-0.65102	41.663	2.5127	0	0.013745	3	131.79	23375000	24	12	19
E1BI98	yes	FLTASRTDPGQDVR	14	21	Unmodified	_FLTASRTDPGQDVF		MULTI-MS	3	521.602233	1561.78487	1.4667	17.455	0.41097	0	0.014201	1	102.5	33049000	28	47	118
P02453	yes	FSGLDGAKGDAGPA	14	21	Unmodified	_FSGLDGAKGDAGP		MULTI-MS	2	631.804217	1261.59388	-0.18464	21.785	1.1478	0	1.8569E-52	3	205.52	678830000	4	52	131
A0A3Q1N	no	GGGYEFGFDGDFYR	14	21	Unmodified	_GGGYEFGFDGDFY		MULTI-MS	2	793.830963	1585.64737	0.23437	67.045	1.9624	-1.4211E-14	1.351E-149	5	266.03	590960000	5	78	253
F1N0H9;A	no	GGVGDLGGAGIPGG	14	21	Unmodified	_GGVGDLGGAGIPG		MULTI-MS	2	542.275095	1082.53564	-0.22505	44.841	0.83777	0	0.013582	1	100.02	171820000	21;20;6	82	285
F1N0H9;A	no	GGVLGAGQPFPIGG	14	21	Unmodified	_GGVLGAGQPFPIG		MULTI-MS	2	613.830037	1225.64552	0.060654	65.476	1.964	0	0.015966	1	95.741	535960000	21;20;6	83	288
P02453	yes	GPPSGGYDLSFLPQ	14	21	Unmodified	_GPPSGGYDLSFLP		MULTI-MS	2	717.848624	1433.6827	0.10501	70.166	2.7648	0	4.1532E-66	5	215.38	57393000	4	119	374
A0A3Q1N	no	GPSGGGYEFGFDGD	14	21	Unmodified	_GPSGGGYEFGFDG		MULTI-MS	2	681.267664	1360.52077	1.4553	56.663	1.2058	7.1054E-15	3.376E-29	2	186.67	23694000	5	137	484
F1N0H9;P	no	GQPFPIGGGAGGLG	14	21	Unmodified	_GQPFPIGGGAGGL		MSMS	2	592.806562	1183.59857	NaN	54.65	1	0	0.0033686	1	88.181		21;6	144	582
F1N0H9;A	no	GVGVPGLGVGVGVP	14	21	Unmodified	_GVGVPGLGVGVGV		MULTI-MS	2	582.342781	1162.67101	-0.042871	72.09	1.4819	1.4211E-14	0.01369	1	100.45	8553300	21;20	155	597
F1N0H9;A	no	GVPGVGVPGVGVPG	14	21	Unmodified	_GVPGVGVPGVGVP		MULTI-MS	2	574.327131	1146.63971	-1.1029	57.265	0.91838	0	0.013307	1	114.6	51595000	21;20;6	172	624
F1N0H9;A	no	GVSTGAVVPQLGAG	14	21	Unmodified	_GVSTGAVVPQLGA		MULTI-MS	2	606.832777	1211.651	1.3194	46.494	0.44863	0	0.019914	1	92.838	7353300	21;20;6	175	631
F1N0H9;A	no	GVYPGGVLPGAGAR	14	21	Unmodified	_GVYPGGVLPGAGA		MULTI-MS	2	635.848761	1269.68297	0.8632	34.111	0.82722	0	0.013987	1	101.65	93883000	21;20;6	177	638
F1N0H9;A	no	PGGVPGGVFFPGAG	14	21	Unmodified	_PGGVPGGVFFPGA		MULTI-MS	2	608.31148	1214.60841	-0.28311	67.523	1.0677	-1.4211E-14	0.01257	1	117.16	13259000	21;20;6	267	775
A0A3Q1N	no	SGGGYEFGFDGDFY	14	21	Unmodified	_SGGGYEFGFDGDF		MULTI-MS	2	759.296421	1516.57829	0.82619	79.15	2.1574	0	0.011206	2	139.02	94318000	5	298	872
A0A3Q1M	no	SPIFPGGAGGLGVG	14	21	Unmodified	_SPIFPGGAGGLGVG		MULTI-MS	2	593.316763	1184.61897	-0.62715	63.095	0.84414	0	0.01298	1	115.71	61428000	20	306	915
F1N0H9;A	no	VGVAPGVGVVPGVG	14	21	Unmodified	_VGVAPGVGVVPGV		MULTI-MS	2	582.342781	1162.67101	-0.76812	57.766	0.61008	0	0.01257	1	117.16	20314000	21;20;6	346	984
F1N0H9;A	no	VGVPGVGVPGVGVP	14	21	Unmodified	_VGVPGVGVPGVGV		MSMS	2	595.350606	1188.68666	NaN	67.9	1	0	0.00072336	1	95.352		21;20;6	347	986
P02453	yes	YDEKSTGISVPGPM	14	21	Oxidation (	_YDEKSTGISVPGPM		MULTI-SEC	2	748.850506	1495.68646	-1.7409	33.378	1.0033	0	0.035019	1	65.347	20156000	4	385	1056
P02453	yes	FLPQPPQEKAHDGGR	15	30	Unmodified	_FLPQPPQEKAHDG		MULTI-MS	3	559.621627	1675.84305	-3.6849	12.872	1.5561	1.7764E-15	0.013376	3	101.2	65642000	4	44	97
E1BB91	yes	FVSEIVDTVYEGGDS	15	30	Unmodified	_FVSEIVDTVYEGGD		MULTI-MA	2	808.86995	1615.72535	8.4316	63.727	0.46429	0	NaN	0	NaN	5501800	26	56	138
P02453	yes	GAVGPAGKDGEAGAQ	15	30	Unmodified	_GAVGPAGKDGEAG		MULTI-MS	2	642.812573	1283.61059	-0.41685	7.9017	0.41044	0	2.7226E-21	1	181.66	73550000	4	65	151
A0A3Q1N	no	GDPGKAGEKGHAGLA	15	30	Unmodified	_GDPGKAGEKGHAG		MSMS	2	682.84949	1363.68443	NaN	7.2469	1	0	0.0019761	1	81.625		5	66	153
P02453	yes	GFSGLDGAKGDAGPA	15	30	Unmodified	_GFSGLDGAKGDAG		MULTI-MS	2	660.314948	1318.61534	-0.77902	24.639	1.4112	0	1.0171E-29	3	185.99	4282900000	4	74	218
A0A3Q1N	no	GGGYEFGFDGDFYRA	15	30	Unmodified	_GGGYEFGFDGDFY		MULTI-MS	2	829.34952	1656.68449	0.24531	65.78	2.7413	0	9.9774E-21	6	176.48	73598000	5	79	271
A0A3Q1N	no	GLQGVQGGKGEQGPA	15	30	Unmodified	_GLQGVQGGKGEQ		MULTI-MS	2	691.854772	1381.69499	-0.061382	10.555	0.31958	0	0.000082315	1	145.91	36888000	5	99	314
A0A3Q1N	no	GPAGANGDRGEAGPA	15	30	Deamidation	_GPAGAN(de)GDRG		MULTI-SEC	2	649.292005	1296.56946	3.5706	8.0878	0.086039	0	0.0038951	1	97.911	1524800	5	100	319
F1N0H9;A	no	GPFGGQQPGVPLGYP	15	30	Unmodified	_GPFGGQQPGVPLG		MULTI-MS	2	735.872434	1469.73031	0.49961	65.238	1.4808	0	0.0067928	2	131.22	54153000	21;20	105	327
F1N0H9;A	no	GPGVKPAKPGVGGLA	15	30	Unmodified	_GPGVKPAKPGVGG		MULTI-MS	2	652.887887	1303.76122	-1.1338	13.474	0.29453	-1.7764E-15	0.015786	1	93.111	10112000	21;20	107	330
A0A3Q1N	no	GPSGGGYEFGFDGDF	15	30	Unmodified	_GPSGGGYEFGFDG		MULTI-MS	2	754.801871	1507.58919	0.63524	72.754	1.4754	0	1.2644E-08	2	159.79	8219500	5	138	488
F1N0H9;A	no	GVGGLGVSTGAVVPQ	15	30	Unmodified	_GVGGLGVSTGAVV		MULTI-MS	2	649.359159	1296.70376	-0.58817	48.753	0.91497	0	0.019225	1	105.36	19943000	21;20;6	152	593
F1N0H9;A	no	GVGVPGLGVGAGVPG	15	30	Unmodified	_GVGVPGLGVGAGV		MULTI-MS	2	596.837862	1191.66117	0.15885	60.206	1.0058	7.1054E-15	0.012198	1	95.531	149620000	21;20	154	596
F1N0H9;A	no	GVGVPGLGVGVGVPG	15	30	Unmodified	_GVGVPGLGVGVGV		MULTI-MS	2	610.853512	1219.69247	0.056649	68.822	1.2673	1.4211E-14	0.011893	1	100.15	55499000	21;20	156	598
P04985	yes	GVGVPGVGVPGALSP	15	30	Unmodified	_GVGVPGVGVPGAL		MSMS	2	631.858795	1261.70304	NaN	64.603	1	0	0.00072613	1	91.069		6	157	601
F1N0H9;A	no	GVGVPGVGVPGVGVP	15	30	Unmodified	_GVGVPGVGVPGVG		MSMS	2	623.861338	1245.70812	NaN	68.864	1	0	7.6683E-06	1	110.81		21;20;6	159	607
F1N0H9;A	no	GVGVVPGVGVVPGVG	15	30	Unmodified	_GVGVVPGVGVVPG		MULTI-MS	2	624.869163	1247.72377	-0.49304	69.405	2.0759	-1.4211E-14	0.0079831	2	122.13	178380000	21;20;6	164	613
F1N0H9;P	no	GVLGAGQPFPIGGGA	15	30	Unmodified	_GVLGAGQPFPIGG		MULTI-MS	2	649.348594	1296.68264	1.0426	65.114	0.8693	0	0.0066878	1	135	56635000	21;6	167	616
A0A3Q1M	no	GVLGAGQPFPIGGVA	15	30	Unmodified	_GVLGAGQPFPIGG		MULTI-MS	2	670.372069	1338.72959	0.46416	73.177	1.0453	-1.4211E-14	0.0090185	2	117.89	107440000	20	169	618
F1N0H9;A	no	GVPGLGVGVGVPGLG	15	30	Unmodified	_GVPGLGVGVGVPG		MULTI-MS	2	617.861338	1233.70812	-0.15511	76.202	0.65644	0	0.031154	1	84.568	32623000	21;20	170	619
F1N0H9;A	no	GVPGVGVPGVGVPGA	15	30	Unmodified	_GVPGVGVPGVGVP		MSMS	2	609.845687	1217.67682	NaN	60.729	1	0	0.00032301	1	95.531		21;20;6	173	625
E1BB91	yes	IDGSQSAGPEFQYIR	15	30	Unmodified	_IDGSQSAGPEFQYI		MULTI-MA	2	834.404826	1666.7951	6.9733	36.647	0.17115	0	NaN	0	NaN	4415000	26	191	659
E1BB91	yes	IVDTVYEGGDSIQVG	15	30	Unmodified	_IVDTVYEGGDSIQV		MULTI-MS	2	776.380485	1550.74642	3.0601	53.725	0.46685	0	0.014593	1	102.07	8849700	26	215	685
A0A3Q1N	no	PSGGGYEFGFDGDFY	15	30	Unmodified	_PSGGGYEFGFDGD		MULTI-MS	2	807.822803	1613.63105	0.9381	78.886	1.2991	0	2.3986E-09	2	163.65	24217000	5	277	802
G3MZI7	yes	SAQESQAQAILQQAR	15	30	Unmodified	_SAQESQAQAILQQA		MULTI-MS	3	543.616542	1627.8278	-0.53309	31.817	0.43138	0	0.015943	1	111.98	13851000	31	287	843
A0A3Q1N	no	SGGGYEFGFDGDFYR	15	30	Unmodified	_SGGGYEFGFDGDF		MULTI-MS	2	837.346977	1672.6794	0.041015	62.562	1.4601	0	4.9854E-53	3	205.56	157500000	5	299	883
E1BB91	yes	VAQKGLEPKVDTILR	15	30	Unmodified	_VAQKGLEPKVDTILF		MULTI-MS	4	417.501715	1665.97776	-0.64982	24.084	0.56121	0	0.012322	1	100.45	19717000	26	322	941
E1BB91	yes	VAVVTYNNEVTTEIR	15	30	Unmodified	_VAVVTYNNEVTTEIF		MULTI-MS	2	854.449234	1706.88391	-0.0033381	39.436	0.74894	-7.1054E-15	0.006886	2	127.87	10898000	26	326	947
F1N0H9;A	no	VGGIGGVGGLGVSTG	15	30	Unmodified	_VGGIGGVGGLGVST		MULTI-MS	2	593.824952	1185.63535	-1.1669	50.639	0.84699	7.1054E-15	0.011776	1	95.815	30614000	21;20;6	336	967
E1BB91	yes	YPPPAVESDAADIVF	15	30	Unmodified	_YPPPAVESDAADIVF		MULTI-MS	2	795.887946	1589.76134	0.54659	74.26	1.2399	0	0.006806	1	130.75	15024000	26	389	1062
F1N0H9;A	no	AVPGAVGLGGVSPAAA	16	17	Unmodified	_AVPGAVGLGGVSP		MULTI-MS	2	647.361702	1292.70885	1.0115	49.653	1.021	0	0.022207	1	88.37	12917000	21;20;6	20	29
F1N0H9;P	no	GGVLGAGQPFPIGGGA	16	17	Unmodified	_GGVLGAGQPFPIG		MULTI-MS	2	677.859326	1353.7041	-0.30565	65.276	1.0879	0	0.016809	2	105.52	92460000	21;6	84	290
A0A3Q1M	no	GGVLGAGQPFPIGGVA	16	17	Unmodified	_GGVLGAGQPFPIG		MULTI-MS	2	698.882801	1395.75105	0.32367	73.169	1.485	-1.4211E-14	0.0020312	1	136.57	162470000	20	86	292
F1N0H9;A	no	GLGGVLGAGQPFPIGG	16	17	Unmodified	_GLGGVLGAGQPFPI		MULTI-MS	2	698.882801	1395.75105	0.71343	76.602	0.9668	0	0.024988	1	107.69	49162000	21;20;6	96	308
G3MZI7	yes	GPPGEVIQPLPIQASR	16	17	Unmodified	_GPPGEVIQPLPIQA		MULTI-MS	2	829.964854	1657.91515	-1.3464	46.386	0.7362	0	0.017732	1	96.067	6206500	31	111	341
P02453	yes	GPPSGGYDLSFLPQPP	16	17	Unmodified	_GPPSGGYDLSFLP		MULTI-MS	2	814.901388	1627.78822	-0.10339	76.158	1.8634	0	2.1519E-23	2	183	43968000	4	121	385
A0A3Q1N	no	GPSGGGYEFGFDGDFY	16	17	Unmodified	_GPSGGGYEFGFDG		MULTI-MS	1	1671.65979	1670.65252	0.097237	77.359	0.78284	0	0	2	349.91	110400000	5	139	508
F1N0H9;A	no	GVGGIGGVGGLGVSTG	16	17	Unmodified	_GVGGIGGVGGLGV		MULTI-MS	2	622.335684	1242.65681	0.45145	53.057	1.2347	0	0.0098063	1	121.85	110550000	21;20;6	149	588
F1N0H9;A	no	GVGVPGVGVPGVGVPG	16	17	Unmodified	_GVGVPGVGVPGVG		MULTI-MS	2	652.372069	1302.72959	-0.018872	66.127	0.90707	0	0.017358	2	96.711	66966000	21;20;6	160	609
F1N0H9;P	no	GVLGAGQPFPIGGGAG	16	17	Unmodified	_GVLGAGQPFPIGG		MULTI-MS	2	677.859326	1353.7041	-1.0769	63.502	0.58361	-7.1054E-15	0.026792	1	86.744	18023000	21;6	168	617
F1N0H9;A	no	PGVGVVPGVGVVPGVG	16	17	Unmodified	_PGVGVVPGVGVVP		MULTI-MS	2	673.395544	1344.77654	-0.0017491	71.566	0.71415	0	0.0028962	1	133.55	6309700	21;20;6	269	777
A0A3Q1N	no	PSGGGYEFGFDGDFYR	16	17	Unmodified	_PSGGGYEFGFDGD		MULTI-MS	2	885.873359	1769.73216	-0.085006	63.093	0.61727	0	6.3807E-43	2	199.8	59912000	5	278	811
A0A3Q1N	no	SGGGYEFGFDGDFYRA	16	17	Unmodified	_SGGGYEFGFDGDF		MULTI-MS	2	872.865534	1743.71651	-0.26216	69.455	1.9283	0	1.6739E-10	3	164.04	31714000	5	300	902
F1N0H9;A	no	VGGIGGVGGLGVSTGA	16	17	Unmodified	_VGGIGGVGGLGVST		MULTI-MS	2	629.343509	1256.67246	-1.325	51.788	0.95229	-7.1054E-15	0.000015941	2	146.79	264080000	21;20;6	337	969
F1N0H9;A	no	VGPFGGQQPGVPLGYP	16	17	Unmodified	_VGPFGGQQPGVPL		MULTI-MS	2	785.40664	1568.79873	0.3464	72.012	2.1597	0	0.0034784	2	131.62	56726000	21;20	343	980
G3MZI7	yes	VSAQESQAQAILQQAR	16	17	Unmodified	_VSAQESQAQAILQQ		MULTI-MS	2	864.455382	1726.89621	-2.7331	31.371	0.65441	0	0.017399	2	110.66	20999000	31	371	1024
G5E5Y4	no	VSGPNINGLRPNNSPI	16	17	2 Deamida	_VSGPN(de)IN(de)GL		MULTI-MS	2	825.925926	1649.8373	1.1354	59.924	0.76735	0	0.016763	1	97.734	47999000	22	372	1033

F1N0H9;A(	no	APGVGVVPGVGVVPGVG	17	14	Unmodifiec	_APGVGVVPGVGVVI	MULTI-MS	2	708.914101	1415.81365	0.46669	72.126	1.9084	0	0.026008	2	111.95	474450000	21;20;6	15	23
F1N0H9;P(	no	GGVLGAGQPFPIGGGAG	17	14	Unmodifiec	_GGVLGAGQPFPIGC	MULTI-MS	2	706.370058	1410.72556	0.18975	63.739	0.68216	0	0.021644	1	118.67	43242000	21;6	85	291
F1N0H9;A(	no	GPGVGVAPGVGVVPGVG	17	14	Unmodifiec	_GPGVGVAPGVGVV	MULTI-MS	2	687.890626	1373.7667	0.29736	61.902	0.71393	0	0.025684	1	115.49	22797000	21;20;6	106	329
G3MZI7	yes	GPPGEVIQPLPIQASRT	17	14	Deamidatic	_GPPGEVIQPLPIQ(d	MULTI-MS	2	880.980701	1759.94685	-0.088838	49.381	1.3044	-7.1054E-15	5.6249E-70	3	218.62	32459000	31	112	346
P02453	yes	GPPSGGYDLSFLPQPPQ	17	14	Unmodifiec	_GPPSGGYDLSFLPC	MULTI-MS	2	878.930677	1755.8468	-0.93441	74.309	4.0193	0	2.1059E-100	7	232.88	185150000	4	122	390
A0A3Q1N/	no	GPSGGGYEFGFDGDFYR	17	14	Unmodifiec	_GPSGGGYEFGFDG	MULTI-MS	2	914.384091	1826.75363	-0.06675	63.574	2.9599	0	0	7	449.79	1767200000	5	140	540
F1N0H9;A(	no	GVGGIGGVGGLGVSTGA	17	14	Unmodifiec	_GVGGIGGVGGLGV:	MULTI-MS	2	657.854241	1313.69393	-1.2123	53.946	0.5093	-7.1054E-15	0.0020815	1	132.13	439740000	21;20;6	150	591
F1N0H9;A(	no	GVGVGVPGLGVGAGVPG	17	14	Unmodifiec	_GVGVGVPGLGVGA	MULTI-MS	2	674.882801	1347.75105	-0.46369	67.081	1.1942	0	0.026008	1	111.95	55874000	21;20	153	594
F1N0H9;A(	no	IGGVGGLGVSTGAVVPQ	17	14	Unmodifiec	_IGGVGGLGVSTGA\	MULTI-MS	2	734.411923	1466.80929	0.072816	58.563	0.71449	7.1054E-15	0.023831	1	117.7	23847000	21;20;6	197	665
F1N0H9;P(	no	LGGVLGAGQPFPIGGGA	17	14	Unmodifiec	_LGGVLGAGQPFPIG	MULTI-MS	2	734.401358	1466.78816	-0.36353	73.324	1.1641	0	0.016421	1	120.99	44929000	21;6	231	708
P02453	yes	LSFLPQPPQEKAHDGGR	17	14	Unmodifiec	_LSFLPQPPQEKAHD	MULTI-MA	4	469.997063	1875.95914	0.92702	26.47	0.14827	0	NaN	0	NaN	2284300	4	249	740
G3MZI7	yes	MVSAQESQAQAILQQAR	17	14	Oxidation (	_M(ox)VSAQESQAQ/	MULTI-MS	2	937.973081	1873.93161	-1.3187	31.982	0.13783	0	0.026334	1	108.37	1854300	31	262	767
A0A3Q1N/	no	PSGGGYEFGFDGDFYRA	17	14	Unmodifiec	_PSGGGYEFGFDGD	MULTI-MS	2	921.391916	1840.76928	-2.7619	64.701	0.70903	0	0.0024648	2	131.06	8154800	5	279	823
E1BI98	yes	VLVTGKAAEYDVVFGER	17	14	Unmodifiec	_VLVTGKAAEYDVVF	MULTI-MS	3	618.331631	1851.97306	-0.045481	46.084	0.74806	0	0.024711	1	90.223	11409000	28	364	1009
F1N0H9;A(	no	AAAAAAKAAAKLGAGGAG	18	8	Unmodifiec	_AAAAAAKAAAKLGA	MULTI-MS	2	699.396607	1396.77866	-0.16076	12.085	0.22609	0	0.0017308	1	130.94	3424800	21;20;6	0	0
A0A3Q1MI	no	GGVLGAGQPFPIGGVAAR	18	8	Unmodifiec	_GGVLGAGQPFPIGC	MULTI-MS	2	812.451914	1622.88927	-1.1064	55.622	0.45102	0	0.02402	1	118.4	11192000	20	87	294
P02453	yes	GPPSGGYDLSFLPQPPQE	18	8	Unmodifiec	_GPPSGGYDLSFLPC	MULTI-MS	2	943.451973	1884.88939	-0.8309	74.74	3.1576	0	3.3766E-75	7	221.55	245680000	4	123	402
A0A3Q1N/	no	GPSGGGYEFGFDGDFYRA	18	8	Unmodifiec	_GPSGGGYEFGFDG	MULTI-MS	2	949.902648	1897.79074	-0.16811	71.882	3.0825	0	0	5	390.17	49214000	5	141	574
F1N0H9;A(	no	GVGVPGVGVPGVGVPGVG	18	8	Unmodifiec	_GVGVPGVGVPGVG	MULTI-MS	2	730.417008	1458.81946	0.46857	74.361	0.49001	0	0.00095542	1	133.23	6718300	21;20;6	162	611
F1N0H9;A(	no	GVPGVGVPGVGVPGVGVP	18	8	Unmodifiec	_GVPGVGVPGVGVP	MULTI-MS	2	750.432658	1498.85076	0.23656	75.988	0.48041	0	6.6425E-08	1	148.05	6063700	21;20;6	174	628
F1N0H9;A(	no	LGVGGIGGVGGLGVSTGA	18	8	Unmodifiec	_LGVGGIGGVGGLG\	MULTI-MS	2	714.396273	1426.77799	0.33241	66.064	1.5069	0	0.027023	1	107.79	75246000	21;20;6	232	709
F1N0H9;A(	no	VAPGVGVVPGVGVVPGVG	18	8	Unmodifiec	_VAPGVGVVPGVGV	MULTI-MS	2	758.448308	1514.88206	-0.56675	74.73	2.1826	0	0.028223	1	106.42	2280100000	21;20;6	321	939
P02453	yes	GPPSGGYDLSFLPQPPQEK	19	3	Unmodifiec	_GPPSGGYDLSFLPC	MULTI-MS	2	1007.49945	2012.98436	1.5351	58.875	2.0274	-7.1054E-15	1.7379E-60	2	208.27	9073500	4	124	411
E1BA17	no	VADADYSELVSIGSKPSAR	19	3	Unmodifiec	_VADADYSELVSIGSI	MULTI-MA	3	655.668971	1963.98508	-0.32679	34.88	0.06876	0	NaN	0	NaN	952780	25	319	937
F1N0H9;A(	no	VGVPGVGVPGVGVPGVGVP	19	3	Unmodifiec	_VGVPGVGVPGVGV	MULTI-MS	2	799.966865	1597.91918	-0.7073	77.745	0.42175	0	0.023717	1	114.84	9166900	21;20;6	348	987
F1N0H9;A(	no	AAAGLPAGVPGLGVGVGVPG	20	3	Unmodifiec	_AAAGLPAGVPGLG\	MULTI-MS	2	808.461947	1614.90934	2.3643	76.322	0.80033	0	0.031947	1	103.7	12561000	21;20	2	2
F1N0H9;A(	no	VGGIGGVGGLGVSTGAVVPQ	20	3	Unmodifiec	_VGGIGGVGGLGVST	MULTI-MS	2	840.967593	1679.92063	0.81996	65.926	1.3369	0	6.5548E-53	1	201.75	94682000	21;20;6	338	971
F1N0H9;A(	no	VGVPGVGVPGVGVPGVGVPG	20	3	Unmodifiec	_VGVPGVGVPGVGV	MULTI-MS	2	828.477597	1654.94064	0.54583	76.871	0.29607	0	0.020841	1	114.36	16609000	21;20;6	349	988
F1N0H9;A(	no	GAPGAIPGIGGIAGVGAPDAA	21	2	Unmodifiec	_GAPGAIPGIGGIAG\	MULTI-MS	2	844.951943	1687.88933	0.15217	71.421	0.69102	0	1.2057E-06	1	136.49	69496000	21;20;6	60	144
F1N0H9;A(	no	GVGGIGGVGGLGVSTGAVVPQ	21	2	Unmodifiec	_GVGGIGGVGGLGV:	MULTI-MS	2	869.478325	1736.9421	1.0259	67.508	1.745	0	9.5534E-41	1	186.87	82329000	21;20;6	151	592
F1N0H9;A(	no	GAPGAIPGIGGIAGVGAPDAAA	22	2	Unmodifiec	_GAPGAIPGIGGIAG\	MULTI-MS	2	880.4705	1758.92645	0.59476	72.511	1.465	0	0.025957	1	111.5	31095000	21;20;6	61	146
F1N0H9;A(	no	LGVGGIGGVGGLGVSTGAVVPQ	22	2	Unmodifiec	_LGVGGIGGVGGLG\	MULTI-MS	2	926.020357	1850.02616	0.12529	75.365	0.47033	0	1.329E-52	1	194.17	29730000	21;20;6	233	710
P02453	yes	GPPSGGYDLSFLPQPPQEKAHDG	23	1	Unmodifiec	_GPPSGGYDLSFLPC	MULTI-MS	3	798.716873	2393.12879	-2.6162	52.937	1.976	0	0.015412	1	87.496	45280000	4	127	428
P02453	yes	PPSGGYDLSFLPQPPQEKAHDGGR	24	1	Unmodifiec	_PPSGGYDLSFLPQF	MULTI-MA	4	638.314751	2549.2299	-2.732	40.92	0.6375	0	NaN	0	NaN	5862800	4	275	787
P02453	yes	GPPSGGYDLSFLPQPPQEKAHDGGR	25	1	Deamidatic	_GPPSGGYDLSFLPC	MULTI-MS	4	652.816121	2607.23538	1.1599	46.406	5.2487	0	2.7129E-22	8	155.76	110750000	4	129	462

Bovine dermis

Accession	Unique protein	Sequence	Length	Count	Modification	Modified sequence	Missed cleavage	Type	Charge	m/z	Mass	ppm	Retention time	Retention length	Retention time calibration	PEP	MS/MS count	Score	Intensity	Protein group ID	Peptide ID	Evidence ID
A0A3Q1NA44	no	EFGFDGDFYR	8	13	Unmodified	_GFDGDFYR_		MULTI-MS	2	488.711599	975.408646	0.62351	26.112	0.67396	3.5527E-15	0.016438	2	151.51	147440000	5	69	157
A0A3Q1NA44	no	FDGNWNGDRSTYG	8	13	Unmodified	_GFDGDFYR_		MULTI-MS	2	488.711599	975.408646	0.77447	26.691	0.6809	0	0.019411	1	151.51	84822000	5	69	158
A0A3Q1NA44	no	FTDGVDGDLADLQRASEELRQEG	8	13	Unmodified	_GFDGDFYR_		MULTI-SE(	2	488.711599	975.408646	-0.89297	27.799	0.55436	0	0.030592	1	79.116	22437000	5	69	163
A0A3Q1NA44	no	FVITGGKSVEDAQEASMA	8	13	Unmodified	_GFDGDFYR_		MULTI-MS	2	488.711599	975.408646	1.2232	28.467	0.89713	0	0.019411	1	151.51	26979000	5	69	159
A0A3Q1NA44	no	GAPGAIPGVPGVGGVPGVGIPA	8	13	Unmodified	_FDGDFYRA_		MULTI-MS	2	495.719424	989.424296	1.1246	26.294	0.81613	0	0.030631	1	148.33	14177000	5	32	50
A0A3Q1NA44	no	GFDGDFYRA	8	13	Unmodified	_FDGDFYRA_		MULTI-SE(	2	495.719424	989.424296	0.72399	28.27	0.89911	3.5527E-15	0.0015766	1	100.19	46875000	5	32	53
A0A3Q1NA44	no	GGGYEFGFDGDFYRA	8	13	Unmodified	_GFDGDFYR_		MULTI-MA	2	488.711599	975.408646	-0.82364	36.303	0.33623	0	NaN	0	NaN	2733700	5	69	185
E1BB91	yes	LGAIAKDPKSETGTR	8	13	Unmodified	_VVIPTFRQ_		MULTI-MS	2	480.287277	958.560001	-0.85882	30.003	0.62653	0	0.025629	1	131.04	13604000	26	379	1044
E1BB91	yes	MVSAQESQAQAILQQAR	8	13	Unmodified	_SFQEVLRF_		MULTI-SE(	2	513.274367	1024.53418	-0.27076	45.873	0.32803	0	0.030592	1	79.116	6393100	26	296	862
E1BA17	no	STVGALDKIGADGTQ	8	13	Unmodified	_FLENLVTA_		MULTI-SE(	1	906.49311	905.485833	-0.45365	54.444	0.34626	0	0.02227	1	82.671	6953500	25	40	81
A4IFU5	no	VISERVTQLSREELSR	8	13	Unmodified	_GLQFPVGR_		MULTI-SE(	2	437.250695	872.486836	0.14809	26.04	1.2232	-3.5527E-15	0.035905	1	76.847	158440000	18	97	309
P21793	yes	VVIPTFRQ	8	13	Unmodified	_APLVKLER_		MULTI-MS	2	463.295102	924.575651	1.142	9.9031	0.5348	-1.7764E-15	0.023532	1	121.99	139590000	9	16	24
A0A3Q1LT99	no	YPPPAVESDAADIVF	8	13	2 Deamidation (NQ)	_KYN(de)KLFN(de)R_		MULTI-MS	3	362.197708	1083.57129	0.00027718	8.4623	0.50671	-1.7764E-15	0.027601	1	120.62	28815000	19	223	697
A0A3Q1NA44	no	FDQHQNKPEILNLVKR	9	11	Unmodified	_GERGVVGPQ_		MSMS	2	449.740691	897.466829	NaN	6.6371	1	0	2.9148E-07	1	107.01		5	68	155
P02453;G3MZI7	no	GVGVPGVGVPGVGVPGVGVPG	9	11	Unmodified	_GPAGEKGAP_		MULTI-MS	2	392.20341	782.392267	-1.4043	6.3545	0.16181	0	0.01427	1	130.28	137600000	4;31	101	320
P02453	yes	GVPGVGVPGVGVP	9	11	Unmodified	_SFLPQPPQE_		MSMS	2	521.763832	1041.51311	NaN	43.622	1	0	0.032068	1	76.774		4	290	849
F1N0H9;A0A3Q1MFJ9;P04985	no	ILVGLERVANLEQ	9	11	Unmodified	_APGIGLGPG_		MULTI-SE(	1	738.414465	737.407189	0.91217	26.533	0.57232	0	0.043312	1	86.344	8730700	21;20;6	14	21
E1BB91	yes	LSFLPQPPQEKAHDGGR	9	11	Unmodified	_ILVGSAGSR_		MULTI-MS	2	430.253434	858.492316	-0.35351	8.4679	0.28953	0	0.01076	1	135.77	25805000	26	204	672
E1BB91	yes	LVLFLGGKSQDDISR	9	11	Unmodified	_IFKRPLGSR_		MULTI-MS	3	358.557459	1072.65055	-0.32112	6.5377	0.25762	0	0.013769	1	131.06	72878000	26	195	663
F1MKG2	yes	SFLPQPPQEKAHD	9	11	Unmodified	_INQVVQGAR_		MULTI-MS	2	492.78289	983.551228	0.62953	6.8476	0.24062	0	6.9747E-06	1	166.17	26950000	29	205	673
E1BA17	no	STGAVVPQLGAG	9	11	Unmodified	_VIVVITDGR_		MULTI-MS	2	486.297842	970.581131	1.0932	22.514	1.0469	3.5527E-15	0.011215	2	143.61	21134000	25	358	999
A4IFU5	no	VGVVQYSHEGTFEA	9	11	Unmodified	_AGLQFPVGR_		MULTI-MS	2	472.769252	943.52395	1.3768	28.485	0.84723	3.5527E-15	0.013958	1	130.77	64131000	18	6	8
F1N0H9;A0A3Q1MFJ9;P04985	no	VTGKAAEYDVVFGER	9	11	Unmodified	_APGIGLGPG_		MULTI-SE(	1	738.414465	737.407189	0.91217	26.533	0.57232	0	0.043312	1	86.344	8730700	21;20;6	14	21
Q3ZBN5	yes	YDEKSTGISVPGPM	9	11	Unmodified	_VVELEDFKR_		MULTI-MS	3	378.876634	1133.60807	-0.71652	18.081	0.67125	0	0.022841	1	124.23	50205000	16	377	1040
A0A3Q1NA44	no	GFSGLDGAKGDA	10	7	Unmodified	_FGFDGDFYRA_		MULTI-MS	2	597.764363	1193.51417	-0.42533	51.824	0.82937	0	0.03453	1	116.25	9252500	5	38	79
E1BB91	yes	LGGVLGAGQPFPIGG	10	7	Unmodified	_AGPEFQYIRT_		MULTI-MS	2	591.301113	1180.58767	0.075626	29.668	0.70432	0	0.015618	1	125.98	38832000	26	7	9
E1BB91	yes	LVDYLDVGFDTTR	10	7	Unmodified	_ILVGLERVAN_		MULTI-MS	2	542.329673	1082.64479	-0.054623	31.036	0.40502	0	0.0041865	1	133.98	12902000	26	202	670
E1BB91	yes	PGISLVKENYAE	10	7	Unmodified	_RELPSLEQKL_		MULTI-MS	2	606.85097	1211.68739	-0.78313	20.995	0.94306	0	0.0018156	1	145.72	41041000	26	284	837
E1BB91	yes	PPSGGYDLSFLPQPPQEKAHDGGR	10	7	Unmodified	_IIDAINKVVY_		MULTI-MS	2	574.339707	1146.66486	0.37038	38.972	0.61483	0	0.0087436	1	130.75	30941000	26	198	666
F1MKG2	no	RYQKSTELLIR	10	7	Unmodified	_GGLHFSDLVE_		MULTI-MS	2	537.266739	1072.51892	-0.65902	40.583	1.1836	0	0.026098	1	119.89	46249000	29	80	283
F1MKG2	no	SAQESQAQAILQQAR	10	7	Unmodified	_AYNKLIKESR_		MSMS	3	407.903183	1220.68772	NaN	6.5253	1	0	0.033856	1	69.979		29	22	31
P02453	yes	GPSGGGYEFGFDGDFY	11	4	Unmodified	_FSGLDGAKGDA_		MULTI-MS	2	519.248546	1036.48254	-0.90686	12.166	0.95458	-1.7764E-15	0.011392	1	122.28	317400000	4	51	124
E1BB91	yes	PPQEKAHDGGR	11	4	Unmodified	_VTQLSREELSR_		MULTI-MS	3	439.908886	1316.70483	0.19555	9.303	0.36459	1.7764E-15	0.021347	1	115.29	17447000	26	376	1039
E1BGN3	no	TADTLKLYQNK	11	4	Unmodified	_RYQKSTELLIR_		MULTI-MS	3	469.608659	1405.80415	0.61934	8.9299	0.54837	-1.7764E-15	0.00044054	1	141.71	46643000	13	286	839
A6QQQ3	no	VLLPKKTESHHKAKG	11	4	Unmodified	_IPPGVFSKLEN_		MULTI-MS	2	600.834788	1199.65502	-0.22276	34.093	1.6736	0	0.014758	1	117.7	35977000	17	206	674
A0A3Q1NA44	no	FDGSGDQNVFVAQKGLEPKVDTILR	12	20	Unmodified	_GLAGHHGDQGAP_		MULTI-MS	2	558.762686	1115.51082	-0.86408	6.4228	0.94191	0	0.033177	1	95.909	3096700000	5	94	306
A0A3Q1NA44	no	GADGAPGKDGVRGLT	12	20	Deamidation (NQ)	_GIRGHN(de)GLDGLK_		MULTI-MS	3	413.226438	1236.65748	-0.30256	8.704	0.45287	0	0.005914	1	110.64	22086000	5	93	305
A0A3Q1NA44	no	GAVGPAGKDGEAGAQ	12	20	Unmodified	_GGYEFGFDGDFY_		MULTI-MS	2	687.269675	1372.5248	0.37634	76.422	1.2676	0	0.034063	1	95.531	43120000	5	88	295
A0A3Q1NA44	no	GFPGAVGAKGEGGPQ	12	20	Deamidation (NQ)	_GIRGHN(de)GLDGLK_		MULTI-MS	3	413.226438	1236.65748	-0.8087	8.2518	0.70516	0	0.011613	1	105.65	6035700	5	93	304
P02453	yes	GYDEKSTGISVPGPM	12	20	Unmodified	_GFSGLDGAKGDA_		MULTI-MA	2	547.759278	1093.504	0.046815	18.19	0.097076	0	NaN	0	NaN	1059000	4	73	209
F1N0H9;A0A3Q1MFJ9	no	GYDLSFLPQPPQE	12	20	Unmodified	_VGPFGGQQPGVP_		MULTI-MS	2	570.29583	1138.57711	-0.033296	42.819	2.3047	0	0.0040436	3	113.89	130620000	21;20	341	974
F1N0H9;A0A3Q1MFJ9;P04985	no	ILINNKISKISPGA	12	20	Unmodified	_STGAVVPQLGAG_		MULTI-MS	2	528.787838	1055.56112	-0.63072	33.073	0.93124	0	0.0044078	1	113.26	31875000	21;20;6	307	917
F1N0H9;A0A3Q1MFJ9;P04985	no	ISQLQDELYLDQVA	12	20	Unmodified	_VPGAVGLGGVSP_		MULTI-MA	2	505.287474	1008.5604	0.74377	44.953	0.85508	0	NaN	0	NaN	4638900	21;20;6	365	1013
E1BB91	yes	LVGVILDNGGKT	12	20	Unmodified	_ELPSLEQKLLTP_		MULTI-SE(	2	684.392667	1366.77078	-1.4387	53.673	0.8763	0	0.024229	1	92.538	50487000	26	29	45
E1BB91	yes	PGGVPGGVFFPGAG	12	20	Unmodified	_VDYLDVGFDTTR_		MULTI-MS	2	700.838256	1399.66196	1.0587	50.843	0.86335	7.1054E-15	0.004193	1	113.63	54980000	26	328	955
E1BI98	yes	PSGGGYEFGFDGDFYR	12	20	Unmodified	_IAKDFIVKVIDR_		MULTI-MS	4	354.969785	1415.85004	-2.207	31.23	0.47533	3.5527E-15	0.02715	1	98.531	64204000	28	189	656
E1BI98	yes	PSGGGYEFGFDGDFYRA	12	20	Oxidation (M)	_TDVM(ox)DALGYVTR_		MULTI-MS	2	678.826834	1355.63912	-0.68796	42.612	0.66122	0	0.026348	1	98.898	10388000	28	314	928
E1BGN3	no	SYGYDEKSTGISVPGPM	12	20	Unmodified	_YRPGTVALREIR_		MULTI-MS	4	358.461122	1429.81538	0.55209	12.738	0.59688	-1.7764E-15	0.0044258	2	113.22	22526000	13	390	1064
E1BGN3	no	TDVMDALGYVTR	12	20	Oxidation (M)	_VTIM(ox)PKDIQLAR_		MULTI-MS	3	467.602517	1399.78572	-0.37774	23.887	1.3247	0	0.013321	1	104.87	549720000	13	375	1037
E1BGN3	no	TGSALDFVR	12	20	Oxidation (M)	_VTIM(ox)PKDIQLAR_		MULTI-MS	2	700.900137	1399.78572	-0.10587	23.887	0.72548	0	0.001779	1	127.76	119140000	13	375	1036
F1N0H9;A0A3Q1MFJ9	no	TIMPKDIQLAR	12	20	Unmodified	_VGPFGGQQPGVP_		MULTI-MS	2	570.29583	1138.57711	-0.033296	42.819	2.3047	0	0.0040436	3	113.89	130620000	21;20	341	974
A0A3Q1MFJ9	no	VAVVTYNNEVTTEIR	12	20	Unmodified	_SPIFPGGAGGLG_		MULTI-MS	2	515.271824	1028.5291	-0.010741	47.992	0.75482	7.1054E-15	0.026934	1	98.629	46567000	20	305	914
F1N0H9;A0A3Q1MFJ9;P04985	no	VFTTEEVPAQQYLEIDEVTPDSFR	12	20	Unmodified	_STGAVVPQLGAG_		MULTI-MS	2	528.787838	1055.56112	-0.63072	33.073	0.93124	0	0.0044078	1	113.26	31875000	21;20;6	307	917
F1N0H9;A0A3Q1MFJ9;P04985	no	VGVPGVGVPGVGVP	12	20	Unmodified	_VPGAVGLGGVSP_		MULTI-MA	2	505.287474	1008.5604	0.74377	44.953	0.85508	0	NaN	0	NaN	4638900	21;20;6	365	1013
F1N0H9;A0A3Q1MFJ9;P04985	no	VTIMPKDIQLAR	12	20	Unmodified	_VPGAVGLGGVSP_		MULTI-MA	2	505.287474	1008.5604	0.74377	44.953	0.85508	0	NaN	0	NaN	4638900	21;20;6	365	1013
A0A3Q1NA44	no	FGFDGDFYRA	13	34	Unmodified	_GGGYEFGFDGDFY_		MULTI-MS	2	715.780407	1429.54626	0.0785	75.29	0.58308	0	0.0087708	1	125.31	151190000	5	77	230
A0A3Q1NA44	no	FLTASRTDPGQDVR	13	34	Unmodified	_GGYEFGFDGDFYR_		MULTI-MS	2	765.320231	1528.62591	0.92047	59.402	1.0316	0	0.010667	1	107.83	12487000	5	89	296
A0A3Q1NA44	no	FSGLDGAKGDA	13	34	Unmodified	_GGGYEFGFDGDFY_		MULTI-MS	2	715.780407	1429.54626	0.71436	76.047	0.69111	0	0.0089876	1	124.08	1009100000	5	77	231
A0A3Q1NA44	no	GGLHFSDLVE	13	34	Unmodified	_GGGYEFGFDGDFY_		MULTI-MA	2	715.780407	1429.54626	-1.003	77.886	0.99475	0	NaN	0	NaN	11037000	5	77	237
P02453	yes	GPSGGGYEFGFDGDF	13	34	Deamidation (NQ)	_PQ(de)PPQEKAHDGGR_		MSMS	2	709.344572	1416.67459	NaN	41.303	1	0	1.5795E-52	1	163.66		4	276	792
P02453	yes	GPSGLPGER	13	34	Unmodified	_FLPQPPQEKAHDG_		MSMS	2	732.367515	1462.72048	NaN	12.125	1	0	0.017982	1	85.29		4	43	88
P02453	yes	GTMLMLPFR	13	34	Unmodified	_SFLPQPPQEKAHD_		MULTI-SE(	3	498.584291	1492.73104	-0.35375	20.449	1.555	0	0.041933	1	63.181	17364000	4	291	850
P02453	yes	GVGVPGVGVPGVGVPG	13	34	Deamidation (NQ)	_FLPQ(de)PPQEKAHDG_		MULTI-MS	3	488.908774	1463.70449	0.87994	15.889	1.5403	-1.7764E-15	0.026692	1	93.623	24327000	4	43	89
P02453	yes	GVPGVGVPGVGVPG	13	34	Unmodified	_GPPSGGYDLSFLP_		MSMS	2	653.819335	1305.62412	NaN	70.345	1	0	0.012867	1	89.355		4	118	365
F1N0H9;A0A3Q1MFJ9;P04985	no	IAFVITGGKSVEDAQEASMA	13	34	Unmodified	_GVVPGVGVVPGVG_		MULTI-SE(	2	546.824224	1091.63389	-0.58014	58.564	0.67071	-7.1054E-15	0.044421	1	62.659	15233000	21;20;6	176	634
F1N0H9;A0A3Q1MFJ9;P04985	no	IDGSQSAGPEFQYIRT	13	34	Unmodified	_VYPGGVLPGAGAR_		MULTI-MS	2	607.33803	1212.66151	0.12794	25.153	1.7047	-3.5527E-15	0.022228	1	96.331	606720000	21;20;6	384	1051

F1N0H9;A0A3Q1MFJ9;P04985	no	IIDAINKVVY	13	34	Unmodified	_GVGVPGVGVPGVG_	MULTI-MS	2	525.800749	1049.58694	-0.5409	51.447	0.88545	7.1054E-15	0.010536	1	110.63	12734000	21;20;6	158	603
F1N0H9;A0A3Q1MFJ9;P04985	no	IIDAINKVVYKGGR	13	34	Unmodified	_VPGAVGLGGVSPA_	MULTI-SE(	2	540.806031	1079.59751	-0.7505	39.324	0.50542	0	0.032059	1	65.252	7850200	21;20;6	366	1014
F1N0H9;A0A3Q1MFJ9;P04985	no	ISVPGPMGPS	13	34	Unmodified	_VYPGGVLPGAGAR_	MULTI-MA	2	607.33803	1212.66151	1.0385	27.687	0.13801	0	NaN	0	NaN	2277200	21;20;6	384	1054
E1BB91	yes	LIVLTADRSGDDVR	13	34	Unmodified	_VLTADTLKLYQNK_	MULTI-MS	2	753.929948	1505.84534	2.0626	28.258	0.44678	0	0.0096934	1	120.06	5011700	26	363	1005
E1BB91	yes	LLGGPAPNTGAALE	13	34	Unmodified	_VREFRDLPSIEER_	MULTI-MS	3	549.293399	1644.85837	2.8987	22.316	0.61164	0	0.008777	1	125.28	16097000	26	370	1020
E1BB91	yes	LPQPPQEKAHDGGR	13	34	Unmodified	_VVTYNNEVTTEIR_	MULTI-MS	2	769.39647	1536.77839	-0.0044893	25.788	0.46059	0	0.027294	1	93.258	9318600	26	382	1048
E1BB91	yes	LPQPPQEKAHDGGRYY	13	34	Unmodified	_LVDYLDVGFDTTR_	MULTI-SE(	2	757.380288	1512.74602	2.6833	59.073	0.54739	-7.1054E-15	0.040848	1	63.408	8759000	26	254	751
E1BB91	yes	LSYGYDEKSTGISVP	13	34	Unmodified	_VLTADTLKLYQNK_	MSMS	3	502.955724	1505.84534	NaN	28.518	1	0	0.014437	1	81.565		26	363	1006
E1BB91	yes	LSYGYDEKSTGISVPGPM	13	34	Unmodified	_ILVGLERVANLEQ_	MULTI-MS	2	727.422291	1452.83003	0.66767	50.92	0.64069	0	0.010342	1	129	41739000	26	203	671
E1BI98	yes	PSGGYDLSFLPQPPQE	13	34	Unmodified	_GLEDAVNEAKHLG_	MSMS	2	676.843873	1351.67319	NaN	32.987	1	0	0.0096675	1	91.549		28	95	307
F1MKG2	yes	SFLPQPPQE	13	34	Deamidation (NQ)	_IVQ(de)AINQVVQGAR_	MULTI-MS	2	698.898983	1395.78341	-1.36	34.803	0.37291	0	0.0103	1	115.71	4987500	29	218	690
F1MKG2	no	SFQEVLRF	13	34	Unmodified	_IQLDDERIDSLSS_	MULTI-MS	2	745.870285	1489.72602	-0.81238	39.384	0.46965	0	0.016451	1	101.3	21176000	29	208	678
F1MKG2	yes	SGGGYEFGFDGDFY	13	34	Deamidation (NQ)	_IVQAIN(de)QVVQGAR_	MULTI-MS	2	698.898983	1395.78341	0.80883	22.989	0.23918	-3.5527E-15	0.02493	1	94.692	2952900	29	218	689
F1N0H9;A0A3Q1MFJ9;P04985	no	VAQKGLEPKVDTILRR	13	34	Unmodified	_GVVPGVGVVPGVG_	MULTI-SE(	2	546.824224	1091.63389	-0.58014	58.564	0.67071	-7.1054E-15	0.044421	1	62.659	15233000	21;20;6	176	634
F1N0H9;A0A3Q1MFJ9;P04985	no	VFTATEFRPAPLQG	13	34	Unmodified	_VPGAVGLGGVSPA_	MULTI-SE(	2	540.806031	1079.59751	-0.7505	39.324	0.50542	0	0.032059	1	65.252	7850200	21;20;6	366	1014
A4IFU5	no	VIQFSDDPRVEF	13	34	Unmodified	_IRNDEELNKLLGR_	MULTI-MS	4	393.22314	1568.86345	-0.43907	25.179	0.76013	0	0.0064134	1	141.58	23320000	18	209	679
F1N0H9;A0A3Q1MFJ9;P04985	no	VPGAVGLGGVSPA	13	34	Unmodified	_GVVPGVGVVPGVG_	MULTI-SE(	2	546.824224	1091.63389	-0.58014	58.564	0.67071	-7.1054E-15	0.044421	1	62.659	15233000	21;20;6	176	634
F1N0H9;A0A3Q1MFJ9;P04985	no	VPGGVPGGVFFPGAG	13	34	Unmodified	_VYPGGVLPGAGAR_	MULTI-MS	2	607.33803	1212.66151	0.12794	25.153	1.7047	-3.5527E-15	0.022228	1	96.331	606720000	21;20;6	384	1051
F1N0H9;A0A3Q1MFJ9;P04985	no	VREFRDLPSIEER	13	34	Unmodified	_GVGVPGVGVPGVG_	MULTI-MS	2	525.800749	1049.58694	-0.5409	51.447	0.88545	7.1054E-15	0.010536	1	110.63	12734000	21;20;6	158	603
F1N0H9;A0A3Q1MFJ9;P04985	no	VSAQESQAQAILQQAR	13	34	Unmodified	_VPGAVGLGGVSPA_	MULTI-SE(	2	540.806031	1079.59751	-0.7505	39.324	0.50542	0	0.032059	1	65.252	7850200	21;20;6	366	1014
P21793	yes	VVESLDVGPDR	13	34	Deamidation (NQ)	_HLNN(de)NKLVKVPGG_	MULTI-SE(	3	464.264892	1389.77285	0.37958	11.446	0.63822	0	0.023486	1	87.184	22415000	9	183	648
A0A3Q1MNL9	no	VYPGGVLPGAGAR	13	34	Unmodified	_ADSDVSTAIDLFR_	MULTI-MS	2	705.348988	1408.68342	-0.96673	56.677	0.52657	0	0.031534	1	90.685	3889900	10	4	4
E1B9F6	no	YDLSFLPQPPQE	13	34	Oxidation (M)	_VETGVLKPGM(ox)VVT_	MULTI-MS	2	673.37342	1344.73229	0.49019	26.562	0.48386	0	0.015293	1	102.52	16853000	12	329	958
A0A3Q1NA44	no	FAVGNKVADQAELEEIA	14	37	Unmodified	_GGGYEFGFDGDFYR_	MULTI-MS	2	793.830963	1585.64737	0.12512	57.443	0.63914	-7.1054E-15	2.2046E-29	2	188.91	179760000	5	78	240
A0A3Q1NA44	no	GDPGKAGEKGHAGLA	14	37	Unmodified	_SGGGYEFGFDGDFY_	MULTI-MS	2	759.296421	1516.57829	0.30615	76.739	0.91114	0	0.011186	1	123.21	38250000	5	298	867
A0A3Q1NA44	no	GDQGPVGRSGETGAS	14	37	Unmodified	_GGGYEFGFDGDFYR_	MULTI-MS	2	793.830963	1585.64737	-0.17494	58.038	0.68658	7.1054E-15	5.8637E-53	1	210.08	130630000	5	78	241
A0A3Q1NA44	no	GGVLGAGQPFPIGG	14	37	Unmodified	_SGGGYEFGFDGDFY_	MULTI-MA	2	759.296421	1516.57829	-0.28326	77.613	0.32949	0	NaN	0	NaN	5646400	5	298	873
A0A3Q1NA44	no	GGVLGAGQPFPIGGGA	14	37	Unmodified	_GGGYEFGFDGDFYR_	MULTI-MA	2	793.830963	1585.64737	-1.3821	66.537	1.0633	0	NaN	0	NaN	3563200	5	78	260
P02453	yes	GPPSGGYDLSFLPQ	14	37	Unmodified	_GPPSGGYDLSFLPQ_	MULTI-MS	2	717.848624	1433.6827	0.15873	64.424	2.7606	1.4211E-14	2.051E-40	3	200.54	42740000	4	119	370
P02453	yes	GPSGEPGKQGPS	14	37	Unmodified	_FSGLDGAKGDAGPA_	MULTI-MS	2	631.804217	1261.59388	0.087989	20.261	0.9418	0	0.00014833	1	149.33	183730000	4	52	128
P02453	yes	GPSGGGYEFG	14	37	Unmodified	_SYGYDEKSTGISVP_	MSMS	2	751.854103	1501.69365	NaN	34.617	1	0	1.354E-39	1	152.54		4	311	922
P02453	yes	GPSGGGYEFGFDG	14	37	Unmodified	_FSGLDGAKGDAGPA_	MULTI-MS	2	631.804217	1261.59388	1.1244	17.279	0.46745	3.5527E-15	0.015577	1	134.75	88566000	4	52	126
F1N0H9;A0A3Q1MFJ9;P04985	no	GYEFGFDGDFYRA	14	37	Unmodified	_GVPGVGVPGVGVPG_	MULTI-MS	2	574.327131	1146.63971	0.35282	52.625	0.738	0	0.013307	2	114.6	20753000	21;20;6	172	623
F1N0H9;A0A3Q1MFJ9;P04985	no	HANTKVGLEHLR	14	37	Unmodified	_GGVLGAGQPFPIGG_	MULTI-MS	2	613.830037	1225.64552	0.19667	60.206	0.84044	0	0.013439	2	106.04	90121000	21;20;6	83	286
F1N0H9;A0A3Q1MFJ9;P04985	no	HLNNNKLVKVPGG	14	37	Unmodified	_PGGVPGGVFFPGAG_	MULTI-MS	2	608.31148	1214.60841	-0.6032	61.953	0.68252	0	0.024939	1	89.142	8436400	21;20;6	267	774
F1N0H9;A0A3Q1MFJ9;P04985	no	HYSDEVEIIR	14	37	Unmodified	_GVYPGGVLPGAGAR_	MULTI-MS	2	635.848761	1269.68297	0.55252	29.51	0.50131	0	0.021234	1	91.867	14498000	21;20;6	177	637
F1N0H9;A0A3Q1MFJ9;P04985	no	ILVGLERVAN	14	37	Unmodified	_GGVLGAGQPFPIGG_	MULTI-SE(	1	1226.6528	1225.64552	-0.27295	60.25	0.48317	0	0.0090172	1	87.639	5337500	21;20;6	83	287
F1N0H9;A0A3Q1MFJ9;P04985	no	IPPGVFSKLEN	14	37	Unmodified	_VGVPGVGVPGVGVP_	MULTI-MS	2	595.350606	1188.68666	1.0178	63.024	1.1656	0	0.020799	1	92.187	25896000	21;20;6	347	985
E1BB91	yes	LLPPTPNNR	14	37	Unmodified	_FKGGSPLNTGKALE_	MULTI-MS	2	709.885541	1417.75653	-0.278	13.968	0.33695	0	0.015096	1	133.98	16149000	26	39	80
E1BB91	yes	LNAFNTKDEVIDAVR	14	37	Unmodified	_VLFLGGKSQDDISR_	MULTI-MS	3	512.278979	1533.81511	-1.9327	27.07	0.34619	0	0.014615	1	104.17	15563000	26	359	1001
E1BB91	yes	LSFLPQPPQEKAHDG	14	37	Unmodified	_LIVLTADRSGDDVR_	MULTI-MS	3	510.61425	1528.82092	1.4089	22.504	0.83124	0	0.025741	1	88.552	38255000	26	237	715
E1BB91	yes	NNYATMRPDSTEIDQDTINR	14	37	Deamidation (NQ)	_IIDAIN(de)KVVYKGGR_	MULTI-MS	3	516.303236	1545.88788	0.71624	17.497	0.42181	0	0.013715	1	100.55	12640000	26	199	667
E1BB91	yes	NTGNATCFVFNDGF	14	37	Unmodified	_LLGGPAPNTGAALE_	MULTI-MS	2	640.845884	1279.67722	1.4797	40.455	0.39889	0	0.014759	1	109.66	8133800	26	239	717
E1BI98	yes	PSGGYDLSFLPQPPQEKAHDGGR	14	37	Unmodified	_VVQYSGTGQQRPER_	MULTI-MS	3	535.609499	1603.80667	-1.363	6.5607	0.33248	0	0.010745	1	126.95	282890000	28	380	1045
F1MKG2	yes	SDLVEVFSPPGSDR	14	37	Unmodified	_VGSDVDTDVLSKIS_	MULTI-MS	2	717.869753	1433.72495	0.24267	43.066	0.60032	0	4.7561E-29	1	184.03	48978000	29	344	982
F1MKG2	no	SFLPQPPQEKAHDGGR	14	37	Unmodified	_VGVVQYSHEGTFEA_	MULTI-MS	2	761.862263	1521.70997	-0.098282	29.559	0.77506	3.5527E-15	9.1643E-40	1	193.21	25470000	29	350	990
G3MZI7	yes	SGLDGAKGDAGPA	14	37	Unmodified	_AQESQAQAILQQAR_	MULTI-MS	2	771.40516	1540.79577	-0.99069	24.988	0.36824	0	0.013149	1	97.813	8003300	31	17	25
F1N0H9;A0A3Q1MFJ9;P04985	no	TYKSQASVLDAIR	14	37	Unmodified	_GVPGVGVPGVGVPG_	MULTI-MS	2	574.327131	1146.63971	0.35282	52.625	0.738	0	0.013307	2	114.6	20753000	21;20;6	172	623
F1N0H9;A0A3Q1MFJ9;P04985	no	VADADYSELVSIGSKPSAR	14	37	Unmodified	_GGVLGAGQPFPIGG_	MULTI-MS	2	613.830037	1225.64552	0.19667	60.206	0.84044	0	0.013439	2	106.04	90121000	21;20;6	83	286
F1N0H9;A0A3Q1MFJ9;P04985	no	VAITPDHLEPRLS	14	37	Unmodified	_PGGVPGGVFFPGAG_	MULTI-MS	2	608.31148	1214.60841	-0.6032	61.953	0.68252	0	0.024939	1	89.142	8436400	21;20;6	267	774
F1N0H9;A0A3Q1MFJ9;P04985	no	VAQKGLEPKVDTILR	14	37	Unmodified	_GVYPGGVLPGAGAR_	MULTI-MS	2	635.848761	1269.68297	0.55252	29.51	0.50131	0	0.021234	1	91.867	14498000	21;20;6	177	637
F1N0H9;A0A3Q1MFJ9;P04985	no	VGNVQELSELSEQVLET	14	37	Unmodified	_VGVPGVGVPGVGVP_	MULTI-MS	2	595.350606	1188.68666	1.0178	63.024	1.1656	0	0.020799	1	92.187	25896000	21;20;6	347	985
F1N0H9;A0A3Q1MFJ9;P04985	no	VGPFGGQQPGVPLGYP	14	37	Unmodified	_GGVLGAGQPFPIGG_	MULTI-SE(	1	1226.6528	1225.64552	-0.27295	60.25	0.48317	0	0.0090172	1	87.639	5337500	21;20;6	83	287
A4IFU5	no	VIDELDVKPEGTRVA	14	37	Unmodified	_VTIAQGGVLPNIQA_	MULTI-MS	2	690.895909	1379.77726	-1.1723	58.448	0.53225	7.1054E-15	0.013921	1	101.38	8146200	18	374	1035
A5D9E8	no	VIVVITDGR	14	37	Unmodified	_LIEDIEDGTFSKLS_	MULTI-MS	2	783.898511	1565.78247	1.249	53.2	0.57183	0	0.015505	1	136.48	17137000	8	235	712
A5D9E8	no	VLFLGGKSQDDISR	14	37	Unmodified	_LIEDIEDGTFSKLS_	MULTI-MS	2	783.898511	1565.78247	-0.99063	53.949	0.54381	0	0.014411	1	103.35	70557000	8	235	713
F1N0H9;A0A3Q1MFJ9;P04985	no	VLVTGKAAEYDVVFGER	14	37	Unmodified	_GGVLGAGQPFPIGG_	MULTI-MS	2	613.830037	1225.64552	0.19667	60.206	0.84044	0	0.013439	2	106.04	90121000	21;20;6	83	286
F1N0H9;A0A3Q1MFJ9;P04985	no	VPGAVGLGGVSP	14	37	Unmodified	_GVYPGGVLPGAGAR_	MULTI-MS	2	635.848761	1269.68297	0.55252	29.51	0.50131	0	0.021234	1	91.867	14498000	21;20;6	177	637
F1MNF8	no	YGYDEKSTGISVPGPM	14	37	Unmodified	_VFVDLEPTVIDEVR_	MULTI-MS	2	815.93797	1629.86139	-0.95692	64.436	0.79975	0	2.2829E-13	1	170.52	15910000	14	335	966
E1BGY1	yes	YRPGTVALREIR	14	37	2 Deamidation (NQ)	_N(de)TGNATCFVFN(de)DGF_	MULTI-MS	2	754.803556	1507.59256	-0.94339	71.217	2.2336	0	0.013142	1	98.249	86693000	27	266	773
A0A3Q1NA44	no	AGLQFPVGR	15	49	Unmodified	_GGGYEFGFDGDFYRA_	MULTI-MS	2	829.34952	1656.68449	-0.62444	61.265	3.8449	0	1.6153E-08	7	158.47	555900000	5	79	264

A0A3Q1NA44	no	AYNKLIKESR	15	49	Unmodified	_SGGGYEFGFDGDFYR_	MULTI-MS	2	837.346977	1672.6794	0.32519	58.884	1.8481	0	2.1887E-82	3	231.07	47612000	5	299	879
A0A3Q1NA44	no	FGFDGDFYR	15	49	Unmodified	_SGGGYEFGFDGDFYR_	MULTI-MS	2	837.346977	1672.6794	0.55871	57.062	0.70477	-7.1054E-15	0.0067049	1	134.38	18585000	5	299	876
A0A3Q1NA44	no	FLENLVTA	15	49	Deamidation (NQ)	_GPAGKDGRIGQ(de)PGAV_	MULTI-MS	2	690.86514	1379.71573	-0.082839	8.8756	0.17541	1.7764E-15	0.02248	1	88.596	4397800	5	104	325
A0A3Q1NA44	no	FLGGKSQDDISR	15	49	Unmodified	_GGGYEFGFDGDFYRA_	MULTI-MS	2	829.34952	1656.68449	-0.49483	64.489	1.0663	0	0.000051469	1	149.33	46069000	5	79	265
A0A3Q1NA44	no	FLPQPPQE	15	49	Unmodified	_PSGGGYEFGFDGDFY_	MULTI-MS	2	807.822803	1613.63105	0.41779	76.025	0.61375	0	0.019936	1	109.02	59330000	5	277	797
A0A3Q1NA44	no	FLPQPPQEKAHDGGRY	15	49	Deamidation (NQ)	_GPAGAN(de)GDRGEAGPA_	MSMS	2	649.292005	1296.56946	NaN	6.6272	1	0	0.00040115	1	94.465		5	100	318
A0A3Q1NA44	no	FLPQPPQEKAHDGGRYY	15	49	Unmodified	_GGGYEFGFDGDFYRA_	MULTI-SEC	2	829.34952	1656.68449	0.4842	67.529	1.7125	0	0.039188	1	76.655	9590700	5	79	268
A0A3Q1NA44	no	FVAQKGLEPKVDTILR	15	49	Deamidation (NQ)	_GLQGVQ(de)GGKGEQGPA_	MULTI-MS	2	692.34678	1382.67901	1.0819	9.9962	0.48193	-1.7764E-15	0.0000211	1	152.7	66626000	5	99	316
A0A3Q1NA44	no	GAVGAKGEGGPQ	15	49	Deamidation (NQ)	_GLQGVQ(de)GGKGEQGPA_	MULTI-MS	2	692.34678	1382.67901	-1.009	9.5959	0.19688	0	0.018099	1	110.38	13407000	5	99	315
A0A3Q1NA44	no	GFSGLDGAKGDAGPA	15	49	Unmodified	_GGGYEFGFDGDFYRA_	MULTI-MA	2	829.34952	1656.68449	-1.2629	63.467	0.761	7.1054E-15	NaN	0	NaN	14869000	5	79	280
A0A3Q1NA44	no	GGGYEFGFDGDFY	15	49	Unmodified	_SGGGYEFGFDGDFYR_	MULTI-MA	2	837.346977	1672.6794	0.037769	66.038	0.14012	0	NaN	0	NaN	395290	5	299	893
P02453	yes	GPPGEVIQPLPIQASRT	15	49	Unmodified	_LSFLPQPPQEKAHDG_	MULTI-MS	3	555.286133	1662.83657	0.64071	33.371	3.2406	0	0.017712	3	91.812	121010000	4	248	731
P02453	yes	GPPSGGYDLS	15	49	Unmodified	_FLPQPPQEKAHDGGR_	MULTI-MS	4	419.96804	1675.84305	-0.95824	10.602	1.889	0	0.010952	3	99.481	2735700000	4	44	93
P02453	yes	GPPSGGYDLSFLPQP	15	49	Unmodified	_FLPQPPQEKAHDGGR_	MULTI-MS	4	419.96804	1675.84305	-0.45152	9.6186	1.6473	0	0.0083744	3	120.53	3585400000	4	44	90
P02453	yes	GPPSGGYDLSFLPQPPQE	15	49	Unmodified	_GFSGLDGAKGDAGPA_	MULTI-MS	2	660.314948	1318.61534	-0.85765	23.281	1.0552	-3.5527E-15	2.117E-08	2	156.58	1191300000	4	74	214
P02453	yes	GPPSGGYDLSFLPQPPQEKAHDGGR	15	49	Unmodified	_LSFLPQPPQEKAHDG_	MULTI-MS	3	555.286133	1662.83657	0.72586	29.539	2.5995	-3.5527E-15	0.01974	2	90.444	185010000	4	248	730
P02453	yes	GPSGEEGKRGSTGEIGPA	15	49	2 Deamidation (NQ)	_FLPQ(de)PPQ(de)EKAHDGGR_	MULTI-SEC	4	420.460047	1677.81108	0.37602	12.742	0.51816	0	0.0087382	1	89.541	10526000	4	44	107
P02453	yes	GPSGGGYEFGFDGD	15	49	Unmodified	_LSFLPQPPQEKAHDG_	MULTI-MS	2	832.425562	1662.83657	2.1415	33.383	1.1409	0	0.034195	1	83.182	13161000	4	248	732
P02453	yes	GPSGGGYEFGFDGDFYR	15	49	Deamidation (NQ)	_FLPQ(de)PPQEKAHDGGR_	MULTI-MS	3	559.949632	1676.82707	1.07	11.272	1.075	0	0.02821	1	85.909	350370000	4	44	103
P02453	yes	GPSGGGYEFGFDGDFYRA	15	49	Unmodified	_GFSGLDGAKGDAGPA_	MULTI-MS	2	660.314948	1318.61534	-1.7359	24.261	1.2806	0	0.000066894	1	147.62	40121000	4	74	215
P02453	yes	GPSVVLRR	15	49	Oxidation (M)	_GYDEKSTGISVPGPM(ox)_	MULTI-MS	2	777.361238	1552.70792	-0.68984	28.257	1.1531	0	0.000035081	1	151.15	167530000	4	178	639
P02453	yes	GVPGVGVPGVGVPGVGVP	15	49	Deamidation (NQ)	_FLPQ(de)PPQEKAHDGGR_	MULTI-MS	2	839.42081	1676.82707	0.85952	11.308	0.85809	0	0.024351	1	87.667	20703000	4	44	101
P02453	yes	GVVPGVGVVPGVG	15	49	Unmodified	_GFSGLDGAKGDAGPA_	MULTI-MA	2	660.314948	1318.61534	6.7652	27.443	0.13589	0	NaN	0	NaN	5342800	4	74	225
F1N0H9;A0A3Q1MFJ9;P04985	no	GYEFGFDGDFYR	15	49	Unmodified	_VPGGVPGGVFFPGAG_	MULTI-MS	2	657.845687	1313.67682	-0.081128	66.565	1.8237	0	0.012198	2	95.531	125110000	21;20;6	367	1016
F1N0H9;A0A3Q1MFJ9;P04985	no	IAEAKLTSIPKELPST	15	49	Unmodified	_GVGVPGVGVPGVGVP_	MSMS	2	623.861338	1245.70812	NaN	64.468	1	0	2.6039E-29	1	150.17		21;20;6	159	605
F1N0H9;A0A3Q1MFJ9;P04985	no	IAGGTWTPSALK	15	49	Unmodified	_LGGVLGAGQPFPIGG_	MULTI-MS	2	670.372069	1338.72959	-0.076645	68.831	0.81812	0	0.011707	1	100.02	17377000	21;20;6	230	707
F1N0H9;A0A3Q1MFJ9	no	ILVGSAGSR	15	49	Unmodified	_GVGVPGLGVGAGVPG_	MULTI-MS	2	596.837862	1191.66117	0.52688	55.428	0.78343	7.1054E-15	0.031154	1	84.568	43298000	21;20	154	595
F1N0H9;A0A3Q1MFJ9;P04985	no	INQVVQGAR	15	49	Unmodified	_GVGVPGVGVPGVGVP_	MSMS	2	623.861338	1245.70812	NaN	64.024	1	0	0.020157	1	72.547		21;20;6	159	604
F1N0H9;A0A3Q1MFJ9;P04985	no	IQLDDERIDSLSS	15	49	Unmodified	_GVGVPGVGVPGVGVP_	MSMS	2	623.861338	1245.70812	NaN	64.907	1	0	5.3123E-14	1	130.34		21;20;6	159	606
F1N0H9;A0A3Q1MFJ9	no	IRNDEELNKLLGR	15	49	Unmodified	_GPFGGQQPGVPLGYP_	MULTI-MS	2	735.872434	1469.73031	1.9895	60.199	1.6434	0	0.0095277	1	116.73	22590000	21;20	105	326
E1BB91	yes	LDAVSQPAQELKR	15	49	Unmodified	_IDGSQSAGPEFQYIR_	MULTI-MS	2	834.404826	1666.7951	0.59799	36.125	0.66111	0	0.0068801	1	128.08	14202000	26	191	658
E1BB91	yes	LIEDIEDGTFSK	15	49	Deamidation (NQ)	_VAVVTYN(de)NEVTTEIR_	MULTI-MS	2	854.941241	1707.86793	1.2667	39.428	0.40793	0	0.012463	1	100.55	8016400	26	326	948
E1BB91	yes	LLNVHSSKDEVQNAVR	15	49	Unmodified	_FVSEIVDTVYEGGDS_	MULTI-MS	2	808.86995	1615.72535	1.6198	64.067	1.1091	0	0.012851	1	95.09	9315600	26	56	137
E1BB91	yes	LTELHLDGNKITKVDAAS	15	49	Unmodified	_LVLFLGGKSQDDISR_	MULTI-MS	3	549.973667	1646.89917	-0.018669	37.464	0.70126	0	0.016264	1	111.74	32172000	26	256	756
E1BB91	yes	LYRNNYATMRPDSTEIDQDTINR	15	49	Unmodified	_VAVVTYNNEVTTEIR_	MULTI-MS	2	854.449234	1706.88391	1.0951	35.645	0.77174	0	6.4189E-14	1	170.52	17315000	26	326	946
F1MKG2	no	RLTLARKDDDPLNAR	15	49	Unmodified	_LGAIAKDPKSETGTR_	MULTI-MS	4	386.716419	1542.83657	-0.74441	6.5835	0.28355	-8.8818E-16	0.0088676	1	118.51	59250000	29	228	705
F1MKG2	yes	SFLPQPPQ	15	49	Unmodified	_RLTLARKDDDPLNAR_	MULTI-MS	4	439.247146	1752.95948	-0.45715	10.335	0.59171	-1.7764E-15	0.000018432	1	152.99	103460000	29	285	838
E1BA17	no	STGISVPGPM	15	49	Deamidation (NQ)	_LN(de)AFNTKDEVIDAVR_	MULTI-MS	3	569.296698	1704.86826	-1.0698	40.711	0.59591	0	0.033205	1	83.633	7738500	25	243	721
F1N0H9;A0A3Q1MFJ9;P04985	no	TKVGLEHLR	15	49	Unmodified	_VPGGVPGGVFFPGAG_	MULTI-MS	2	657.845687	1313.67682	-0.081128	66.565	1.8237	0	0.012198	2	95.531	125110000	21;20;6	367	1016
F1N0H9;A0A3Q1MFJ9;P04985	no	VETGVLKPGMVVT	15	49	Unmodified	_LGGVLGAGQPFPIGG_	MULTI-MS	2	670.372069	1338.72959	-0.076645	68.831	0.81812	0	0.011707	1	100.02	17377000	21;20;6	230	707
F1N0H9;A0A3Q1MFJ9;P04985	no	VGPFGGQQPGVP	15	49	Unmodified	_GVGVPGVGVPGVGVP_	MSMS	2	623.861338	1245.70812	NaN	64.907	1	0	5.3123E-14	1	130.34		21;20;6	159	606
F1N0H9;A0A3Q1MFJ9	no	VGSDVDTDVLSKIS	15	49	Unmodified	_GVGVPGLGVGAGVPG_	MULTI-MS	2	596.837862	1191.66117	0.52688	55.428	0.78343	7.1054E-15	0.031154	1	84.568	43298000	21;20	154	595
F1N0H9;A0A3Q1MFJ9;P04985	no	VGSHNFDITKR	15	49	Unmodified	_GVGVPGVGVPGVGVP_	MSMS	2	623.861338	1245.70812	NaN	64.024	1	0	0.020157	1	72.547		21;20;6	159	604
A4IFU5	no	VINVVNRLG	15	49	Unmodified	_VLLPKKTESHHKAKG_	MULTI-MS	4	419.001882	1671.97842	-1.0376	6.3371	0.21878	8.8818E-16	0.008149	1	121.45	54924000	18	362	1004
F1N0H9;A0A3Q1MFJ9;P04985	no	VLTADTLKLYQNK	15	49	Unmodified	_VPGGVPGGVFFPGAG_	MULTI-MS	2	657.845687	1313.67682	-0.081128	66.565	1.8237	0	0.012198	2	95.531	125110000	21;20;6	367	1016
F1N0H9;A0A3Q1MFJ9;P04985	no	VPIYEGYALPHA	15	49	Unmodified	_LGGVLGAGQPFPIGG_	MULTI-MS	2	670.372069	1338.72959	-0.076645	68.831	0.81812	0	0.011707	1	100.02	17377000	21;20;6	230	707
F1N0H9;A0A3Q1MFJ9;P04985	no	VTIAQGGVLPNIQA	15	49	Unmodified	_GVGVPGVGVPGVGVP_	MSMS	2	623.861338	1245.70812	NaN	64.024	1	0	0.020157	1	72.547		21;20;6	159	604
P21793	yes	VVTYNNEVTTEIR	15	49	Deamidation (NQ)	_LILINN(de)KISKISPGA_	MULTI-MS	3	527.990658	1580.95014	-0.12438	41.703	0.43497	-7.1054E-15	0.034797	1	82.908	12047000	9	236	714
A0A3Q1NA44	no	APLVKLER	16	26	Unmodified	_GPSGGGYEFGFDGDFY_	MULTI-MS	2	836.333535	1670.65252	0.46631	73.341	1.8336	0	1.7216E-31	3	183.81	43390000	5	139	499
A0A3Q1NA44	no	AQESQAQAILQQAR	16	26	Unmodified	_PSGGGYEFGFDGDFYR_	MULTI-MS	2	885.873359	1769.73216	-0.36858	58.536	1.9284	-7.1054E-15	9.5403E-208	3	287.45	102510000	5	278	806
A0A3Q1NA44	no	AVVTYNNEVTTEIR	16	26	Unmodified	_GPSGGGYEFGFDGDFY_	MULTI-MS	2	836.333535	1670.65252	0.23121	75.54	1.1531	0	2.6334E-69	3	217.12	5195900000	5	139	503
A0A3Q1NA44	no	FAEYQPELFEKFQNMR	16	26	Unmodified	_SGGGYEFGFDGDFYRA_	MULTI-MS	2	872.865534	1743.71651	0.52873	60.165	1.5375	0	0.0038342	2	130.44	29930000	5	300	897
A0A3Q1NA44	no	FDGDFYRA	16	26	Unmodified	_GPSGGGYEFGFDGDFY_	MULTI-MS	2	836.333535	1670.65252	0.45748	77.398	1.9071	0	3.0439E-118	2	244.28	137170000	5	139	506
A0A3Q1NA44	no	FLPQPPQEKAHDG	16	26	Unmodified	_SGGGYEFGFDGDFYRA_	MULTI-MS	2	872.865534	1743.71651	-0.80622	60.862	1.9888	0	1.2078E-55	1	208.17	117580000	5	300	898
A0A3Q1NA44	no	FLPQPPQEKAHDGGR	16	26	Unmodified	_PSGGGYEFGFDGDFYR_	MULTI-MS	2	885.873359	1769.73216	-1.7216	57.98	0.70938	0	5.1644E-84	1	228.78	23445000	5	278	805
A0A3Q1NA44	no	GFDGDFYR	16	26	Unmodified	_GPSGGGYEFGFDGDFY_	MULTI-MS	1	1671.65979	1670.65252	-0.98421	74.919	0.50158	0	3.8732E-207	1	284.62	67315000	5	139	502
A0A3Q1NA44	no	GFSGLDGAK	16	26	Unmodified	_GPSGGGYEFGFDGDFY_	MULTI-MS	2	836.333535	1670.65252	0.35153	74.914	0.50158	0	3.1202E-22	1	174.86	1268200000	5	139	501
P02453	yes	GPPSGGYDLSFLPQPPQEKAHDGG	16	26	Oxidation (M)	_YGYDEKSTGISVPGPM(ox)_	MULTI-MS	2	858.892903	1715.77125	-0.21851	35.969	0.97752	0	0.0016592	2	138.22	92755000	4	387	1058
P02453	yes	GPSGGGYEFGF	16	26	Unmodified	_LPQPPQEKAHDGGRYY_	MULTI-MS	3	619.307708	1854.9013	0.33552	13.874	1.7893	1.7764E-15	0.018103	1	95.428	62473000	4	245	724
F1N0H9;P04985	no	HYSDEVEIIRG	16	26	Unmodified	_GGVLGAGQPFPIGGGA_	MULTI-MS	2	677.859326	1353.7041	-0.57757	60.166	0.88231	-7.1054E-15	0.017578	1	96.331	16083000	21;6	84	289
F1N0H9;A0A3Q1MFJ9	no	IDGSQSAGPEFQYIR	16	26	Unmodified	_VGPFGGQQPGVPLGYP_	MULTI-MS	2	785.40664	1568.79873	0.42967	67.25	1.5963	-1.4211E-14	0.000016927	1	146.27	25778000	21;20	343	979
F1N0H9;A0A3Q1MFJ9;P04985	no	IFHTVGGFDEISPAG	16	26	Unmodified	_GVGVPGVGVPGVGVPG_	MULTI-MS	2	652.372069	1302.72959	-0.61694	61.39	0.56805	-7.1054E-15	0.0041264	1	129.47	25845000	21;20;6	160	608
F1N0H9;A0A3Q1MFJ9;P04985	no	ITMQSPTDSLLYH	16	26	Unmodified	_GVGGIGGVGGLGVSTG_	MULTI-MA	2	622.335684	1242.65681	0.13862	52.855	0.1476	0	NaN	0	NaN	1249300	21;20;6	149	589
E1BB91	yes	LIEDIEDGTFSKLS	16	26	Unmodified	_FDQHQNKPEILNLVKR_	MULTI-MS	4	495.525987	1978.07484	0.44834	22.915	0.61795	0	0.000018336	1	145.55	46600000	26	35	75
E1BB91	yes	LSKDELVKFEPGQSHAG	16	26	Unmodified	_VAQKGLEPKVDTILRR_	MULTI-MS	4	456.526993	1822.07887	-0.5446	16.819	0.76801	0	0.000018054	1	145.69	47247000	26	323	943

E1BB91	yes	NILVGSAGSRIAEGVPQ	16	26	Unmodified	_FVAQKGLEPKVDTILR_	MULTI-MS	4	454.268819	1813.04617	-0.95591	30.158	0.48689	3.5527E-15	0.0050248	1	126.48	17395000	26	54	135
E1BB91	yes	PPSGGYDLSFLPQPPQE	16	26	Unmodified	_IDGSQSAGPEFQYIRT_	MULTI-MS	2	884.928665	1767.84278	0.70866	39.572	0.77799	0	0.024255	1	106.26	23933000	26	192	660
E1BB91	yes	PQPPQEKAHDGGR	16	26	Unmodified	_LLNVHSSKDEVQNAVR_	MULTI-MS	3	603.65863	1807.95406	0.63821	13.79	0.56021	0	0.01753	1	96.414	6366600	26	240	718
G3MZI7	yes	SGGGYEFGFDGDFYR	16	26	Unmodified	_VSAQESQAQAILQQAR_	MULTI-MS	2	864.455382	1726.89621	1.0725	32.609	0.75174	0	2.2817E-259	1	300.78	121460000	31	371	1023
G3MZI7	yes	SPIFPGGAGGLG	16	26	Unmodified	_VSAQESQAQAILQQAR_	MULTI-MA	2	864.455382	1726.89621	-2.8056	31.257	0.17899	0	NaN	0	NaN	5197600	31	371	1026
F1N0H9;A0A3Q1MFJ9	no	VAVVQYSGTGQQRPERAA	16	26	Unmodified	_VGPFGGQQPGVPLGYP_	MULTI-MS	2	785.40664	1568.79873	0.42967	67.25	1.5963	-1.4211E-14	0.000016927	1	146.27	25778000	21;20	343	979
F1N0H9;A0A3Q1MFJ9;P04985	no	VFVAQKGLEPKVDTILR	16	26	Unmodified	_GVGVPGVGVPGVGVPG_	MULTI-MS	2	652.372069	1302.72959	-0.61694	61.39	0.56805	-7.1054E-15	0.0041264	1	129.47	25845000	21;20;6	160	608
F1N0H9;A0A3Q1MFJ9;P04985	no	VGVPGVGVPGVGVPGVGVPG	16	26	Unmodified	_GVGGIGGVGGLGVSTG_	MULTI-MA	2	622.335684	1242.65681	0.13862	52.855	0.1476	0	NaN	0	NaN	1249300	21;20;6	149	589
F1N0H9;A0A3Q1MFJ9;P04985	no	VTQLSREELSR	16	26	Unmodified	_GVGGIGGVGGLGVSTG_	MULTI-MA	2	622.335684	1242.65681	0.13862	52.855	0.1476	0	NaN	0	NaN	1249300	21;20;6	149	589
A0A3Q1NA44	no	AGPEFQYIRT	17	30	Unmodified	_GPSGGGYEFGFDGDFYR_	MULTI-MS	2	914.384091	1826.75363	-0.055365	58.587	2.455	-7.1054E-15	0	6	368.3	2379400000	5	140	527
A0A3Q1NA44	no	ATPAAIEKAVQEAQR	17	30	Unmodified	_GPSGGGYEFGFDGDFYR_	MULTI-MS	2	914.384091	1826.75363	-1.0781	63.32	1.1271	0	7.3095E-23	3	177.16	12216000	5	140	531
A0A3Q1NA44	no	DATDVMDALGYVTR	17	30	Unmodified	_PSGGGYEFGFDGDFYRA_	MULTI-MS	2	921.391916	1840.76928	-0.923	61.033	1.3283	7.1054E-15	4.0008E-289	3	308.74	61043000	5	279	821
A0A3Q1NA44	no	DFIAKVIQR	17	30	Unmodified	_GPSGGGYEFGFDGDFYR_	MULTI-MS	2	914.384091	1826.75363	0.32355	56.759	1.0506	-7.1054E-15	0	3	337.01	245620000	5	140	524
A0A3Q1NA44	no	DYLDVGFDTTR	17	30	Unmodified	_GPSGGGYEFGFDGDFYR_	MULTI-MS	2	914.384091	1826.75363	-0.59198	64.407	1.7485	0	0	2	329.49	45980000	5	140	532
A0A3Q1NA44	no	FKGGSPLNTGKALE	17	30	Unmodified	_GPSGGGYEFGFDGDFYR_	MULTI-MS	2	914.384091	1826.75363	-1.1687	54.869	0.46375	0	0.029166	1	95.264	9841500	5	140	522
A0A3Q1NA44	no	FRELPSLEQKLLTP	17	30	Unmodified	_PSGGGYEFGFDGDFYRA_	MULTI-MS	2	921.391916	1840.76928	-1.0989	60.219	1.0944	0	3.2367E-06	1	149.83	10545000	5	279	820
A0A3Q1NA44	no	FVSEIVDTVYEGGDS	17	30	Unmodified	_GPSGGGYEFGFDGDFYR_	MULTI-SE(	2	914.384091	1826.75363	-0.523	66.033	2.5984	0	3.0072E-07	1	126.51	8463700	5	140	533
A0A3Q1NA44	no	GERGVVGPQ	17	30	Unmodified	_PSGGGYEFGFDGDFYRA_	MULTI-MS	2	921.391916	1840.76928	-1.4299	61.931	0.66293	0	0.030065	1	100.55	8474300	5	279	822
A0A3Q1NA44	no	GGAVPIGIGIGNADITEMQT	17	30	Unmodified	_GPSGGGYEFGFDGDFYR_	MULTI-MA	2	914.384091	1826.75363	-0.37229	69.14	1.4252	0	NaN	0	NaN	1949000	5	140	552
P02453	yes	GGYEFGFDGDFYR	17	30	Unmodified	_GPPSGGYDLSFLPQPPQ_	MULTI-MS	2	878.930677	1755.8468	0.35959	69.477	4.7618	0	1.279E-118	8	242.35	83909000	4	122	387
P02453	yes	GHRGFSGLQ	17	30	Unmodified	_GPPSGGYDLSFLPQPPQ_	MULTI-MS	2	878.930677	1755.8468	0.17161	72.923	3.2477	0	4.2884E-70	7	219.35	36822000	4	122	388
P02453	yes	GPAGEKGAP	17	30	Unmodified	_LSFLPQPPQEKAHDGGR_	MULTI-MS	4	469.997063	1875.95914	-0.4774	23.286	2.5571	0	0.023124	4	99.451	1483500000	4	249	734
P02453	yes	GPAGEKGAPGADGPA	17	30	Unmodified	_LSFLPQPPQEKAHDGGR_	MULTI-MS	2	938.986849	1875.95914	-0.1707	25.849	2.2323	-3.5527E-15	2.6994E-06	4	150.69	21463000	4	249	737
P02453	yes	GPPGFVGEKGPS	17	30	Unmodified	_FLPQPPQEKAHDGGRYY_	MULTI-MS	4	501.499704	2001.96971	-0.25155	21.408	2.6573	0	0.026204	3	109.79	501010000	4	46	109
P02453	yes	GPPSGGYDLSFLPQPPQ	17	30	Unmodified	_LSFLPQPPQEKAHDGGR_	MULTI-MS	2	938.986849	1875.95914	-0.094197	23.284	1.5531	0	3.4622E-10	2	161.21	60995000	4	249	736
P02453	yes	GPPSGGYDLSFLPQPPQEK	17	30	Unmodified	_LSFLPQPPQEKAHDGGR_	MULTI-MS	3	626.326991	1875.95914	-0.55989	23.292	2.349	-3.5527E-15	0.028884	2	102.78	953720000	4	249	735
P02453	yes	GPPSGGYDLSFLPQPPQEKA	17	30	2 Deamidation (NQ)	_LSFLPQ(de)PPQ(de)EKAHDGGR_	MULTI-MS	4	470.48907	1877.92718	2.4527	28.802	1.1816	3.5527E-15	0.023087	2	100.41	11860000	4	249	743
P02453	yes	GPPSGGYDLSFLPQPPQEKAHDG	17	30	Unmodified	_FLPQPPQEKAHDGGRYY_	MULTI-MS	4	501.499704	2001.96971	-0.65009	23.971	2.475	3.5527E-15	0.019674	2	119.54	333770000	4	46	113
P02453	yes	GPSGGGYEFGFD	17	30	Deamidation (NQ)	_LSFLPQPPQ(de)EKAHDGGR_	MULTI-MS	2	939.478857	1876.94316	-1.4728	27.218	0.67871	0	0.026552	1	107.17	20643000	4	249	742
P02453	yes	GVGVPGVGVPGALSP	17	30	Unmodified	_FLPQPPQEKAHDGGRYY_	MULTI-MS	2	1001.99213	2001.96971	-0.33375	21.146	0.98527	0	0.029817	1	86.772	14316000	4	46	111
F1N0H9;A0A3Q1MFJ9;P04985	no	IAKDFIVKVIDR	17	30	Unmodified	_GVGVPGVGVPGVGVPGA_	MULTI-MS	2	687.890626	1373.7667	-0.61587	64.191	0.65189	0	0.0074262	1	124.98	23690000	21;20;6	161	610
E1BB91	yes	KYNKLFNR	17	30	Unmodified	_FAVGNKVADQAELEEIA_	MULTI-MS	2	902.459798	1802.90504	-1.1224	48.813	0.53417	0	0.026107	1	110.86	51414000	26	31	48
E1BB91	yes	LAPSTMKIKIIAPPER	17	30	Unmodified	_VGNVQELSELSEQVLET_	MULTI-MS	2	937.473103	1872.93165	1.1387	66.363	0.60954	0	0.023806	1	88.187	7732200	26	340	973
E1BB91	yes	LILINNKISKISPGA	17	30	Unmodified	_NILVGSAGSRIAEGVPQ_	MULTI-MS	2	834.457393	1666.90023	-0.27913	38.198	0.45282	0	0.034993	1	85.554	8208900	26	263	768
E1BI98	yes	PSGGGYEFGFDGDFY	17	30	Unmodified	_VLVTGKAAEYDVVFGER_	MULTI-MS	3	618.331631	1851.97306	-0.3892	40.737	1.021	0	0.026281	1	108.96	58821000	28	364	1007
E1BI98	yes	QLSYGYDEKSTGISVPGPM	17	30	Unmodified	_VLVTGKAAEYDVVFGER_	MULTI-MA	3	618.331631	1851.97306	2.1499	44.614	0.2792	0	NaN	0	NaN	1367400	28	364	1010
G3MZI7	yes	SGGYDLSFLPQPPQE	17	30	Unmodified	_GPPGEVIQPLPIQASRT_	MULTI-MS	2	880.488693	1758.96283	-0.84813	43.453	0.67383	7.1054E-15	5.158E-32	1	184.34	18809000	31	112	344
F1N0H9;A0A3Q1MFJ9;P04985	no	VFAVVITDGRHDPRDDDLN	17	30	Unmodified	_GVGVPGVGVPGVGVPGA_	MULTI-MS	2	687.890626	1373.7667	-0.61587	64.191	0.65189	0	0.0074262	1	124.98	23690000	21;20;6	161	610
F1N0H9;A0A3Q1MFJ9;P04985	no	VPSYQALLR	17	30	Unmodified	_GVGVPGVGVPGVGVPGA_	MULTI-MS	2	687.890626	1373.7667	-0.61587	64.191	0.65189	0	0.0074262	1	124.98	23690000	21;20;6	161	610
A0A3Q1NA44	no	ADSDVSTAIDLFR	18	16	Unmodified	_GPSGGGYEFGFDGDFYRA_	MULTI-MS	2	949.902648	1897.79074	-0.40579	61.072	4.1535	7.1054E-15	0	10	375.97	1596300000	5	141	558
A0A3Q1NA44	no	APGIGLGPG	18	16	Unmodified	_GPSGGGYEFGFDGDFYRA_	MULTI-MS	2	949.902648	1897.79074	-0.26922	58.9	2.2535	0	0	4	357.95	167660000	5	141	557
A0A3Q1NA44	no	DLSFLPQPPQE	18	16	Unmodified	_GPSGGGYEFGFDGDFYRA_	MULTI-MS	2	949.902648	1897.79074	-0.45734	64.69	2.1205	0	0	3	398.25	50637000	5	141	561
A0A3Q1NA44	no	ELPSLEQKLLTP	18	16	Unmodified	_GPSGGGYEFGFDGDFYRA_	MULTI-MS	2	949.902648	1897.79074	-0.46552	64.045	0.9064	0	0	2	406.94	123090000	5	141	560
A0A3Q1NA44	no	FSGLDGAKGDAGPA	18	16	Unmodified	_GPSGGGYEFGFDGDFYRA_	MULTI-MS	2	949.902648	1897.79074	0.41922	67.6	0.69986	0	2.7548E-189	1	273.27	17348000	5	141	563
A0A3Q1NA44	no	GGGYEFGFDGDFYR	18	16	Unmodified	_GPSGGGYEFGFDGDFYRA_	MULTI-MA	2	949.902648	1897.79074	-0.6851	68.423	1.5	0	NaN	0	NaN	9539100	5	141	577
P02453	yes	GGVLGAGQPFPIGGVA	18	16	Unmodified	_GPPSGGYDLSFLPQPPQE_	MULTI-MS	2	943.451973	1884.88939	0.098096	70.323	4.7137	-1.4211E-14	8.9661E-60	9	203.03	1533900000	4	123	396
P02453	yes	GLEDAVNEAKHLG	18	16	Unmodified	_GPPSGGYDLSFLPQPPQE_	MULTI-MS	2	943.451973	1884.88939	0.13964	73.509	2.7404	0	2.5108E-88	6	224.42	381190000	4	123	398
P02453	yes	GPMVSAQESQAQAILQQAR	18	16	Unmodified	_GPPSGGYDLSFLPQPPQE_	MULTI-MS	3	629.303741	1884.88939	-0.29609	70.223	3.3838	0	0.000017793	4	139.21	97592000	4	123	397
P02453	yes	GPPSGGYDLSFL	18	16	Unmodified	_GPPSGGYDLSFLPQPPQE_	MULTI-MS	3	629.303741	1884.88939	-0.24624	73.497	2.7655	0	0.0001512	3	135.61	30009000	4	123	399
F1N0H9;A0A3Q1MFJ9;P04985	no	IEDIEDGTFSKLS	18	16	Unmodified	_GVPGVGVPGVGVPGVGVP_	MULTI-MS	2	750.432658	1498.85076	0.084564	73.117	0.84276	0	0.03543	1	90.629	2646100	21;20;6	174	627
F1N0H9;A0A3Q1MFJ9;P04985	no	ITSDPRLVFT	18	16	Unmodified	_GVPGVGVPGVGVPGVGVP_	MULTI-MA	2	750.432658	1498.85076	-0.53727	76.065	0.39829	0	NaN	0	NaN	2604800	21;20;6	174	629
F1N0H9;A0A3Q1MFJ9;P04985	no	VDYLDVGFDTTR	18	16	Unmodified	_GVPGVGVPGVGVPGVGVP_	MULTI-MS	2	750.432658	1498.85076	0.084564	73.117	0.84276	0	0.03543	1	90.629	2646100	21;20;6	174	627
F1N0H9;A0A3Q1MFJ9;P04985	no	VVELEDFKR	18	16	Unmodified	_GVPGVGVPGVGVPGVGVP_	MULTI-MA	2	750.432658	1498.85076	-0.53727	76.065	0.39829	0	NaN	0	NaN	2604800	21;20;6	174	629
P21793	yes	VVQYSGTGQQRPER	18	16	Unmodified	_LTELHLDGNKITKVDAAS_	MULTI-MS	3	642.349462	1924.02656	-0.37161	26.887	0.32213	0	5.2934E-08	1	149.47	141100000	9	253	749
P21793	yes	VVQYSHEGTFEAIQLDDERIDS	18	16	Unmodified	_LTELHLDGNKITKVDAAS_	MULTI-MS	2	963.020554	1924.02656	-0.96181	26.897	0.11259	-3.5527E-15	4.5386E-08	1	150.26	9312800	9	253	750
P02453	yes	GVYPGGVLPGAGAR	19	4	Unmodified	_GPPSGGYDLSFLPQPPQEK_	MULTI-MA	2	1007.49945	2012.98436	-1.4512	59.436	0.39694	0	NaN	0	NaN	4639000	4	124	412
G3MZI7	yes	SGGGYEFGFDGDFYRA	19	4	Oxidation (M)	_GPM(ox)VSAQESQAQAILQQAR_	MULTI-MS	3	677.009222	2028.00584	0.48978	40.555	0.87399	7.1054E-15	0.023316	1	115.73	11465000	31	109	339
G3MZI7	yes	SGGYDLSFLPQPPQEKAHDGGR	19	4	Oxidation (M)	_GPM(ox)VSAQESQAQAILQQAR_	MULTI-MS	2	1015.01019	2028.00584	-3.9296	37.431	0.79519	0	1.3795E-88	1	222.99	17567000	31	109	336
E1BA17	no	SYGYDEKSTGISVP	19	4	Unmodified	_VADADYSELVSIGSKPSAR_	MULTI-MS	3	655.668971	1963.98508	-1.159	34.784	0.99447	0	0.032765	1	103.17	27955000	25	319	936
P02453	yes	GPAGKDGRIGQPGAV	20	6	Unmodified	_GPPSGGYDLSFLPQPPQEKA_	MULTI-MS	2	1043.01801	2084.02147	1.4876	55.325	1.7168	0	1.3629E-94	4	226.23	33192000	4	125	415
P02453	yes	GVGVPGVGVPGVGVP	20	6	Deamidation (NQ)	_GPPSGGYDLSFLPQPPQ(de)EKA_	MULTI-SE(	2	1043.51002	2085.00549	1.0462	59.207	0.62853	-7.1054E-15	0.015787	1	111.94	6670700	4	125	418
F1N0H9;A0A3Q1MFJ9;P04985	no	IVQAINQVVQGAR	20	6	Unmodified	_VGVPGVGVPGVGVPGVGVPG_	MULTI-MA	2	828.477597	1654.94064	1.6427	76.179	0.41846	0	NaN	0	NaN	6177900	21;20;6	349	989
E1BB91	yes	LVLISSGKSDDEVEDSAIELKQ	20	6	Oxidation (M)	_GGAVPIGIGIGNADITEM(ox)QT_	MULTI-MS	2	965.482947	1928.95134	0.0020997	63.552	0.76356	0	0.019225	1	116.03	67671000	26	75	228
F1MKG2	no	SFLPQPPQEKAHDG	20	6	Oxidation (M),Deamic	_N(de)NYATM(ox)RPDSTEIDQDTINR_	MULTI-MS	3	791.020404	2370.03938	-0.18866	26.549	0.84971	0	0.020334	1	114.88	21221000	29	265	772
E1BA17	no	STVGALDKIGADGTQVAMVQ	20	6	Oxidation (M)	_STVGALDKIGADGTQVAM(ox)VQ_	MULTI-MS	2	989.001504	1975.98846	0.85361	42.064	0.38358	-7.1054E-15	0.0016098	1	126.95	16940000	25	310	921
F1N0H9;A0A3Q1MFJ9;P04985	no	IALVITDGRSDTQR	21	2	Unmodified	_GVGVPGVGVPGVGVPGVGVPG_	MULTI-MS	2	856.988329	1711.9621	-0.19254	75.211	0.24657	0	0.032679	1	94.309	6348300	21;20;6	163	612
F1N0H9;A0A3Q1MFJ9;P04985	no	VAVVQYSGTGQQRPER	21	2	Unmodified	_GVGVPGVGVPGVGVPGVGVPG_	MULTI-MS	2	856.988329	1711.9621	-0.19254	75.211	0.24657	0	0.032679	1	94.309	6348300	21;20;6	163	612
P02453	yes	GVGGIGGVGGLGVSTG	22	4	Unmodified	_SGGYDLSFLPQPPQEKAHDGGR_	MULTI-MS	3	786.048734	2355.12437	0.0067849	35.79	1.455	0	0.00041355	1	127.01	31910000	4	302	908

F1N0H9;A0A3Q1MFJ9	no	IFKRPLGSR	22	4	Unmodified	_GAPGAIPGVPGVGGVPGVGIPA_	MULTI-MS	2	898.506886	1794.99922	-1.3838	75.715	0.29546	0	0.030449	1	105.86	16958000	21;20	62	147
E1BB91	yes	LSFLPQPPQE	22	4	Unmodified	_LVLISSGKSDDEVEDSAIELKQ_	MULTI-MS	3	792.411115	2374.21152	-0.67386	46.327	0.77474	-7.1054E-15	0.0036688	1	124.31	116370000	26	257	757
F1N0H9;A0A3Q1MFJ9	no	VFVDLEPTVIDEVR	22	4	Unmodified	_GAPGAIPGVPGVGGVPGVGIPA_	MULTI-MS	2	898.506886	1794.99922	-1.3838	75.715	0.29546	0	0.030449	1	105.86	16958000	21;20	62	147
P02453	yes	GGYEFGFDGDFY	23	8	Unmodified	_GPPSGGYDLSFLPQPPQEKAHDG_	MULTI-MS	3	798.716873	2393.12879	-2.0439	51.785	5.4918	-7.1054E-15	7.7449E-23	8	163.95	495840000	4	127	426
P02453	yes	GLQFPVGR	23	8	Unmodified	_GPPSGGYDLSFLPQPPQEKAHDG_	MULTI-MS	2	1197.57167	2393.12879	-0.89934	48.724	2.1488	0	7.6945E-161	5	259.08	46078000	4	127	425
P02453	yes	GPPSGGYDLSFLPQPPQEKAHD	23	8	Unmodified	_PSGGYDLSFLPQPPQEKAHDGGR_	MULTI-MS	4	614.05156	2452.17714	-0.38202	36.247	2.1397	7.1054E-15	0.011874	2	101.6	48263000	4	281	830
P02453	yes	GSAGSPGKDGLNGLP	23	8	Deamidation (NQ)	_GPPSGGYDLSFLPQPPQ(de)EKAHDG_	MULTI-SE(	2	1198.06368	2394.1128	3.5059	50.704	0.508	0	0.015519	1	73.887	5263200	4	127	432
P02453	yes	GVGVPGVGVPGVGVPGA	23	8	Unmodified	_PSGGYDLSFLPQPPQEKAHDGGR_	MULTI-SE(	3	818.399655	2452.17714	0.049013	39.06	1.2257	0	0.037303	1	62.847	11980000	4	281	833
F1MKG2	no	RDSFQEVLR	23	8	Oxidation (M),Deamic	_LYRN(de)NYATM(ox)RPDSTEIDQDTINR_	MULTI-MS	4	701.579248	2802.28789	0.25009	26.007	0.9686	0	3.5097E-22	2	161.8	78850000	29	260	762
F1MKG2	no	RELPSLEQKL	23	8	Oxidation (M),Deamic	_LYRN(de)NYATM(ox)RPDSTEIDQDTINR_	MULTI-MS	4	701.579248	2802.28789	1.0498	25.369	1.1075	0	3.9822E-39	2	183.45	104590000	29	260	761
F1MKG2	no	SFLPQPPQEKAHDGGRYY	23	8	Oxidation (M),2 Dean	_LYRN(de)N(de)YATM(ox)RPDSTEIDQDTINR_	MULTI-MS	4	701.825252	2803.2719	0.71769	29.141	0.88004	-3.5527E-15	0.020481	1	85.945	21943000	29	260	764
P02453	yes	GPPSGGYDLSFLP	24	4	Unmodified	_GPPSGGYDLSFLPQPPQEKAHDGG_	MULTI-MS	3	817.724027	2450.15025	-0.025038	45.999	3.0916	0	2.2106E-15	3	152.48	80310000	4	128	436
P02453	yes	GPPSGGYDLSFLPQPP	24	4	Unmodified	_PPSGGYDLSFLPQPPQEKAHDGGR_	MULTI-MS	4	638.314751	2549.2299	0.6251	40.561	1.8216	0	0.005346	2	92.8	20255000	4	275	784
P02453	yes	GPQGIAGQRGVV	24	4	Unmodified	_PPSGGYDLSFLPQPPQEKAHDGGR_	MULTI-MS	4	638.314751	2549.2299	0.26505	37.686	1.8333	0	0.0064268	1	91.238	20488000	4	275	783
P02453	yes	GVGVPGVGVPGVG	24	4	Unmodified	_PPSGGYDLSFLPQPPQEKAHDGGR_	MULTI-MS	4	638.314751	2549.2299	2.6283	43.741	0.81555	0	0.017386	1	85.248	8057900	4	275	785
P02453	yes	GHNGLDGLK	25	8	Unmodified	_GPPSGGYDLSFLPQPPQEKAHDGGR_	MULTI-MS	3	869.757731	2606.25136	-1.5175	41.39	4.3849	0	5.3983E-88	7	216.67	1642600000	4	129	444
P02453	yes	GIRGHNGLDGLK	25	8	Unmodified	_GPPSGGYDLSFLPQPPQEKAHDGGR_	MULTI-MS	4	652.570117	2606.25136	-0.26894	38.551	3.6589	0	1.6188E-15	7	150.36	4185800000	4	129	441
P02453	yes	GLAGHHGDQGAP	25	8	Unmodified	_GPPSGGYDLSFLPQPPQEKAHDGGR_	MULTI-MS	3	869.757731	2606.25136	-0.33148	38.54	3.5316	0	3.5572E-103	6	221.83	1678000000	4	129	442
P02453	yes	GLQGVQGGKGEQGPA	25	8	Unmodified	_GPPSGGYDLSFLPQPPQEKAHDGGR_	MULTI-MS	4	652.570117	2606.25136	-0.058695	35.296	2.2951	0	0.000694	5	124.81	137450000	4	129	439
P02453	yes	GPAGANGDRGEAGPA	25	8	Unmodified	_GPPSGGYDLSFLPQPPQEKAHDGGR_	MULTI-MS	4	652.570117	2606.25136	-0.050994	44.634	2.795	0	1.4935E-15	5	150.72	1542600000	4	129	447
P02453	yes	GPFGGQQPGVPLGYP	25	8	Unmodified	_GPPSGGYDLSFLPQPPQEKAHDGGR_	MULTI-MS	2	1304.13296	2606.25136	-0.39102	38.418	1.8874	0	3.3305E-183	4	260.25	40355000	4	129	443
P02453	yes	GVGVPGLGVGAGVPG	25	8	Unmodified	_GPPSGGYDLSFLPQPPQEKAHDGGR_	MULTI-SE(	4	652.570117	2606.25136	-2.033	62.671	1.8508	7.1054E-15	0.015255	1	71.756	3817700	4	129	449
E1BB91	yes	LLQFGGPREQQ	25	8	Unmodified	_FDGSGDQNVFVAQKGLEPKVDTILR_	MULTI-MS	4	684.110612	2732.41334	-0.38668	51.781	1.2673	0	5.3421E-23	1	162.82	67895000	26	34	74
