# Supplementary material for: Physical, mechanical, and biological properties of collagen membranes for guided bone regeneration: a comparative in vitro study
Source: BMC Oral Health. 2023 Jul 22;23:510. doi: 10.1186/s12903-023-03223-4 (PMC10362553; doi:10.1186/s12903-023-03223-4)
Supplement: Supplementary file 3 — Additional file 3: Figure 3. Original figure of Figure 3d in the manuscript. (A) Original figure. (B) The picture after exposure. The red boxes represent the regions of the original blots used in main figures. The label (M,1,2,3,4,5,7,6) matched to the cropped versions in the manuscript figures. [file 12903_2023_3223_MOESM3_ESM.pdf]

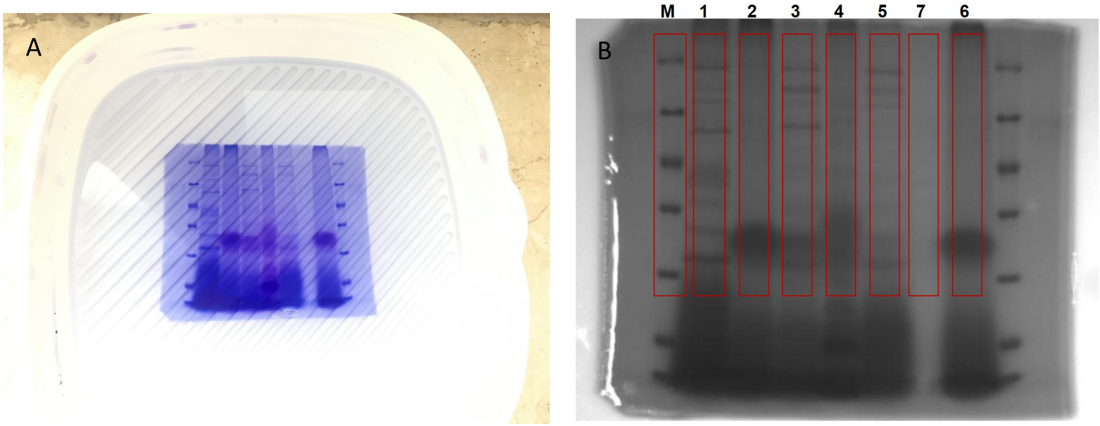

Figure 3. Original figure of Figure 3d in the manuscript. (A) Original figure. (B) The picture after exposure. The red boxes represent the regions of the original blots used in main figures. The label (M,1,2,3,4,5,7,6) matched to the cropped versions in the manuscript figures.
